# Optimization of Selective and CNS Penetrant Alkyne-Based TREK Inhibitors: The Discovery and Characterization of ONO-9517601 (VU6022856) and ONO-7927846 (VU6024391)

**DOI:** 10.1021/acs.jmedchem.5c02535

**Published:** 2025-10-17

**Authors:** Motoyuki Tanaka, Yoko Sekioka, Gakuji Hashimoto, Takahiro Mori, Tomoyuki Shono, Yuuki Isaji, Katsuya Hisaichi, Elizabeth S. Childress, Sean Bollinger, Joza A. Schmitt, Trevor C. Chopko, Aaron T. Garrison, Charles K. Perry, Keagan Chronister, Meghan Kramer, Sichen Chang, Katherine J. Watson, Jonathan W. Dickerson, Michael Bubser, Jerri M. Rook, Carrie K. Jones, Olivier Boutaud, Thomas M. Bridges, Jerod S. Denton, Darren W. Engers, Haruto Kurata, Craig W. Lindsley

**Affiliations:** † Drug Discovery Chemistry, 13369 Ono Pharmaceutical Co., Ltd., 3-1-1 Sakurai, Shimamoto, Mishima, Osaka 618-8585, Japan; ‡ Research Center of Neurology, Ono Pharmaceutical Co., Ltd., 3-1-1 Sakurai, Shimamoto, Mishima, Osaka 618-8585, Japan; § Pharmacokinetic Research, Ono Pharmaceutical Co., Ltd., 3-1-1 Sakurai, Shimamoto, Mishima, Osaka 618-8585, Japan; ∥ Safety Research, Ono Pharmaceutical Co., Ltd., 3-1-1 Sakurai, Shimamoto, Mishima, Osaka 618-8585, Japan; ⊥ Warren Center for Neuroscience Drug Discovery, 5718 Vanderbilt University, Nashville, Tennessee 37232, United States; # Department of Pharmacology, 12327 Vanderbilt University School of Medicine, Nashville, Tennessee 37232, United States; ∇ Department of Anesthesiology, 12328 Vanderbilt University Medical Center, Nashville, Tennessee 37232, United States

## Abstract

Herein we describe the chemical optimization of a selective and CNS penetrant series of TREK inhibitors (the K_2_P family of potassium ion channels), culminating in the discovery of ONO-9517601 (VU6022856) and ONO-7927846 (VU6024391). Optimization of ONO-TR-772 focused on replacements for the *N*-Boc aniline moiety and identified *N*-acyl piperidine pyrazoles as attractive surrogates, affording excellent potency, PK profiles, CNS penetration and ion channel selectivity. ONO-9517601 and ONO-7927846 displayed robust efficacy in an MK-801 challenge rat NOR paradigm, with MEDs of 1 mg/kg and 0.3 mg/kg, respectively. These ligands represent valuable preclinical research tools for exploring selective TREK inhibition *in vitro* and *in vivo*.

## Graphical abstract

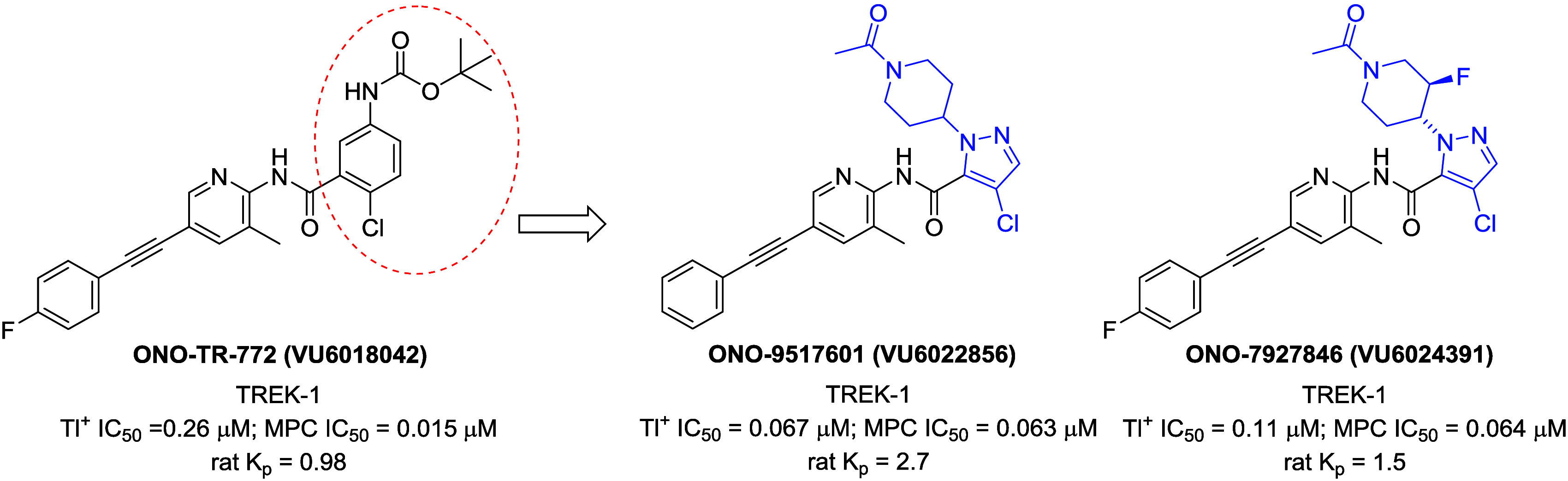

## Introduction

The industrial-academic collaboration between Ono Pharmaceuticals and the Warren Center for Neuroscience Drug Discovery at Vanderbilt focused on a platform of developing activators and inhibitors of various potassium ion channels for the treatment of CNS disorders. The K_2_P family contains 15 K_2_P subtypes, within six distinct subfamilies: tandem of P domains in a weakly inward-rectifying potassium channel (TWIK), TWIK-related acid-sensitive K+ channels (TASK), TWIK-related K+ channels (TREK), TWIK-related alkaline pH-activated K+ channels (TALK), TWIK-related spinal cord K+ channel (TRESK) and tandem pore domain halothane-inhibited K+ channels (THIK).^1–15^ Our first collaborative target was TREK, a channel highly expressed in the peripheral and central nervous systems, and either inhibition or activation could have therapeutic potential in pain, cognition, and other peripheral and CNS disorders.^16^ However, the potential of TREK modulation has long been hindered by the absence of selective small molecule tool compounds.

From this collaboration, we recently disclosed ONO-2920632 (VU6011887) a selective and CNS penetrant TREK-2 preferring activator that displayed nonopiate analgesia.^17^ The TREK inhibitor field had only a few reported small molecules, such as **1** ^18^ and **2** ^19^ (Figure 1) and was largely driven by human genetic data, peptides (such as spadin^20^) and off-target TREK activity by antidepressants,^21^ antipsychotics,^22^ and antihypertensives.^23^ Earlier this year, we disclosed our hit-to-lead campaign for the discovery of TREK inhibitors, launching from the weak HTS hit **3** (TREK-1 Tl^+^ IC_50_ = 7.7 μM).^24^ Initial SAR led to the identification of *N*-Boc benzyl ether **4** (TREK-1 Tl^+^ IC_50_ = 0.46 μM); however, poor PK and a latent quinone-like substructure led to a scaffold-hop to the heterobiaryl acetylene **5** (TREK-1 Tl^+^ IC_50_ = 0.26 μM). ONO-TR-772 (**5**) proved to be a highly selective and CNS penetrant dual TREK-1 and TREK-2 inhibitor with robust efficacy in MK-801 mouse NOR, with a MED of 10 mg/kg by IP dosing. Despite this notable advance, the team was concerned with the aniline core and the *N*-Boc moiety, high lipophilicity (ChromatoLogD = 5.16), high plasma protein binding across species (h,r,m: 99.4%, 99.9% and 98.4%), high brain homogenate binding (r,m: >99.9%, 99.4%) and low aqueous solubility (<5 μM). Here, we will disclose the lead optimization campaign of **5** toward a valuable preclinical research tool for exploring selective TREK inhibition *in vitro* and *in vivo* that addresses these limitations.

**Figure 1:**
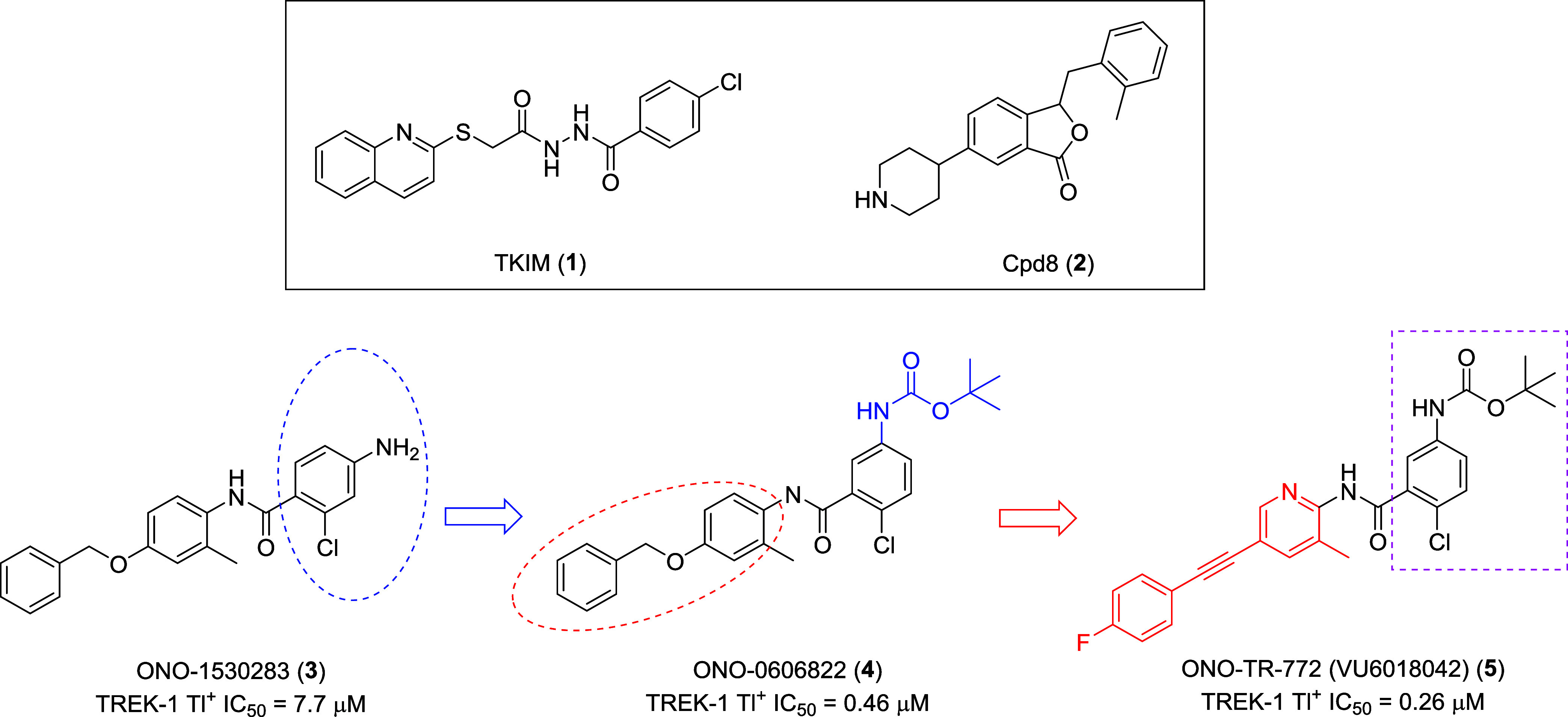
Structures of exemplar TREK inhibitors **1** and **2**, and our recent hit-to-lead effort from HTS hit **3** to *in vivo* tool compound, ONO-TR-772 (**5**) with an MED of 10 mg/kg (IP) in mouse MK-801 NOR assay.

## Results and Discussion

### Chemical Lead Optimization Overview

For the lead optimization of **5**, we undertook a multidimensional strategy aimed at the *N*-Boc anilino moiety of the molecule (Figure 2), wherein we evaluated heteroatom incorporation (**6**), alternate linkers for the anilino amide (**7**), heterocyclic core replacements (**8** and **9**), and, finally, sp^3^-enriched substituents (**10** and **11**) to improve physiochemical properties. Interestingly, a regioisomeric pyrazole **9** indicated to induce a mode switch from TREK inhibitor to TREK activator. Overall, ONO-TR2–772 (**5**) with a human TREK-1 Tl^+^ IC_50_ of 0.26 μM, and a CLogP of 5.53 was optimized to provide ONO-9517601 (**10**) with a human TREK-1 Tl^+^ IC_50_ of 0.067 μM, and a CLogP of 1.35 and ONO-7927846 (**11**) with a human TREK-1 Tl^+^ IC_50_ of 0.11 μM, and a CLogP of 1.79. Now, we will walk through the step-by-step lead optimization and compound profiling that led to the discovery of valuable preclinical research tools **10** and **11**.

**Figure 2:**
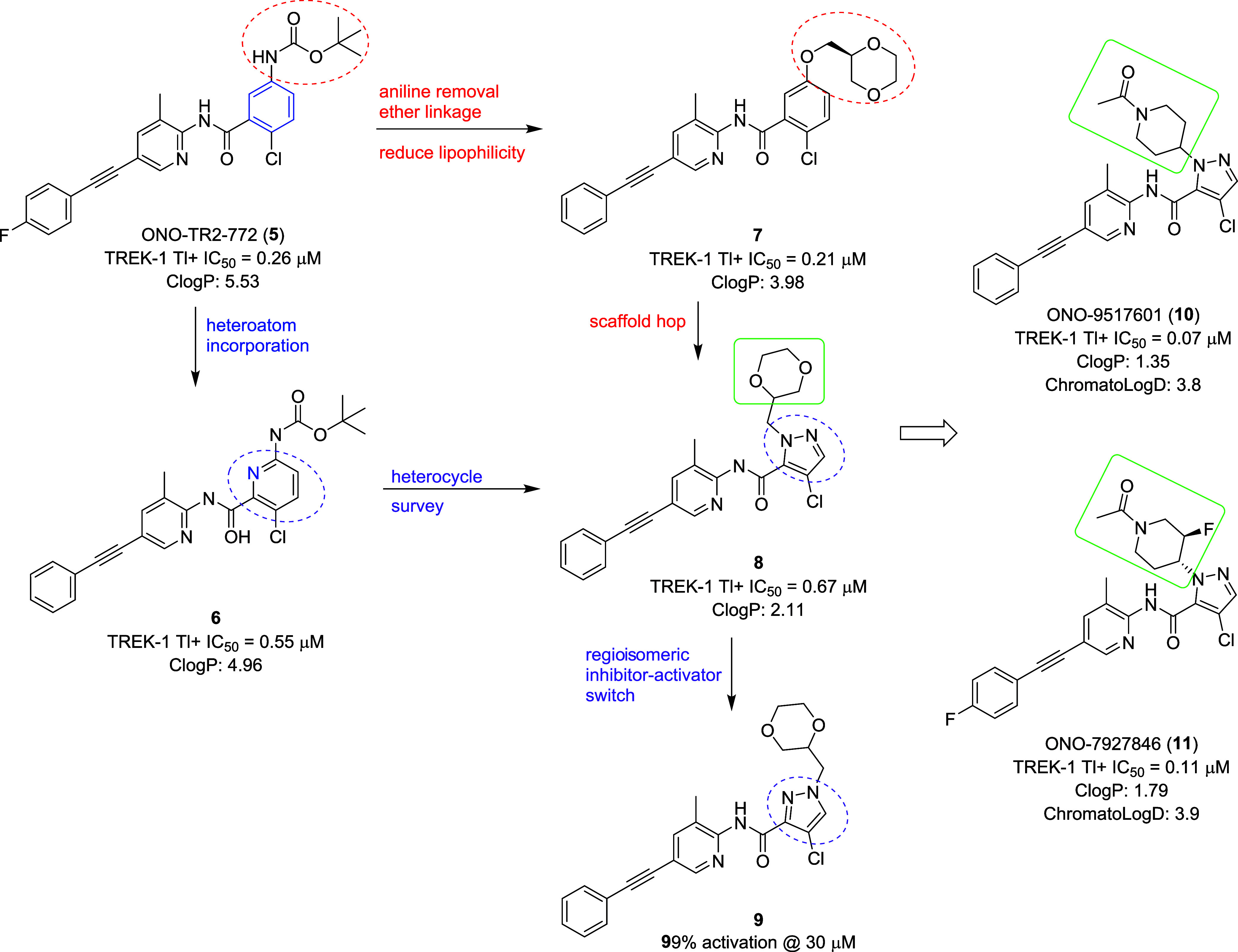
Lead optimization overview from **5**, leading to the discovery of ONO-9517601 (**10**) and ONO-7927846 (**11**) with improved potency and reduced lipophilicity.

The team first focused on the incorporation of nitrogen atoms into the anilino core of **5** to remove the aniline mutagenicity liability and to increase polarity/reduce lipophilicity. As shown in Figure 3, we prepared and evaluated analogs **12**, consisting of regioisomeric pyridine congeners, pyrimidines, pyrazine and pyridazines. However, all proved to be weak or inactive (TREK-1 Tl^+^ IC_50_s > 2 μM) against TREK-1 except for the 2-pyrdiyl congener **6** (TREK-1 Tl^+^ IC_50_ = 0.55 μM), which also showed reduced lipophilicity (CLogP of 4.96). In parallel to this exercise, the team was also exploring alternatives for the aniline linkage as a complementary strategy to remove the aniline mutagenicity liability and to increase polarity/reduce lipophilicity.

**Figure 3:**
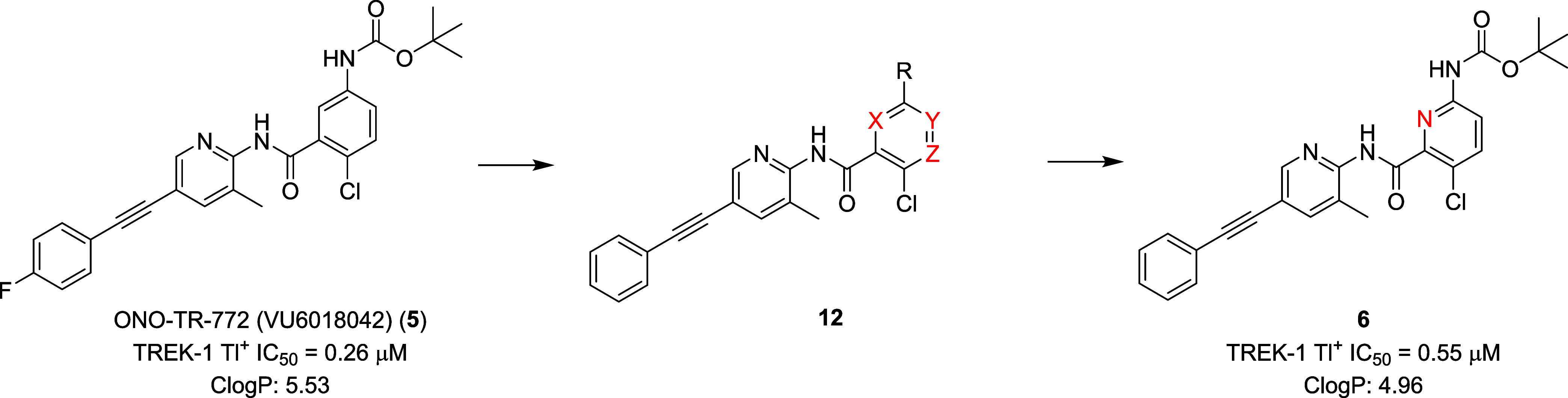
Lead optimization of **5** surveying 6-membered heterocycles to remove the aniline mutagenicity liability and reduce lipophilicity. The 2-pyridine congener **6** was the only heterocyclic replacement that retained appreciable TREK-1 activity.

Here as well, SAR was steep, with few reverse amide analogs possessing TREK inhibitory activity (Table 1). A representative example, **13a**, a 3,3-difluoro azetidinyl amide, displayed ∼2-fold less potency (TREK-1 Tl^+^ IC_50_ = 0.32 μM) than **13e**, reduced lipophilicity *(* CLogP of 4.33), poor rat PK (superhepatic clearance, CL_p_ = 328 mL/min/kg) yet good CNS penetration (rat *K*_p_ = 0.84). An oxadiazole amide bioisostere **13b** proved more potent (TREK-1 Tl^+^ IC_50_ = 0.048 μM) than **13e**, and with reduced lipophilicity (CLogP of 3.61); however, *in vitro* predicted intrinsic clearance was extremely high. Progression to an ether linkage, as in racemic analog **13c**, resolved multiple issues. Ether **13c** was equipotent to **13e** at TREK-1 (TREK-1 Tl^+^ IC_50_ = 0.13 μM), displayed lower lipophilicity (CLogP of 3.98), and while *in vitro* intrinsic clearance was high in mouse, *in vivo* rat PK was attractive (CL_p_ = 16.2 mL/min/kg) with good CNS penetration (rat *K*_p_ = 0.89). Synthesis and screening of the two enantiopure analogs demonstrated that both the (*R*)-congener **13d** and the (*S*)-congener **7** had comparable TREK-1 potencies in the human thallium flux assay (Tl^+^ IC_50_s of 0.11 μM and 0.21 μM, respectively). However, the enantiomers did diverge when assessed in human and mouse TREK-1 manual patch clamp assays. **13d** was very potent on human TREK-1 (MPC IC_50_ = 0.011 μM), but weak against mouse TREK-1 (MPC IC_50_ = 1.2 μM), whereas **7** displayed a more balanced profile on human TREK-1 (MPC IC_50_ = 0.079 μM), and mouse TREK-1 (MPC IC_50_ = 0.17 μM). There was a poor *in vitro*:*in vivo* correlation (IVIVC) for DMPK in rat, with **7** showing high predicted intrinsic clearance, but low *in vivo* clearance in rat (CL_p_ = 9.43 mL/min/kg) and good CNS penetration (rat *K*_p_ = 0.86). While this was a notable advance, a further reduction in lipophilicity was sought, and the team felt that 5-membered heterocycles could be the answer.

**Table 1:**
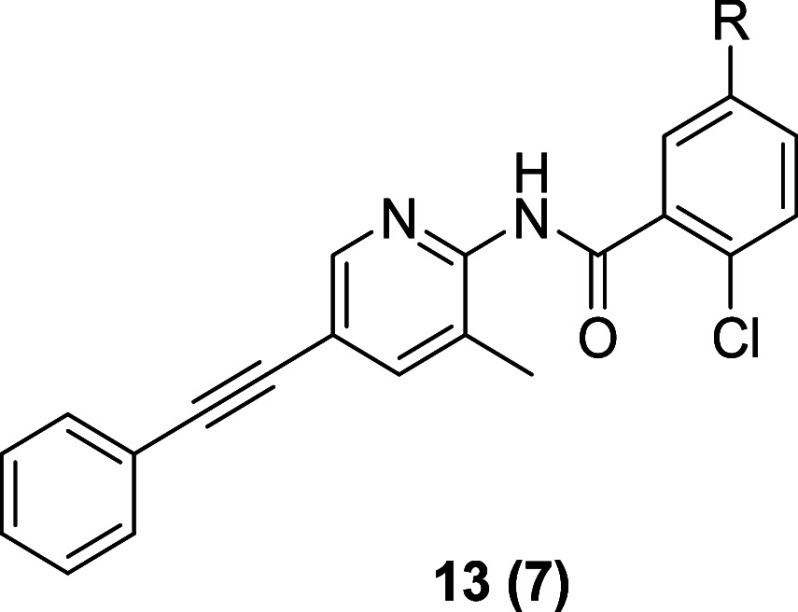

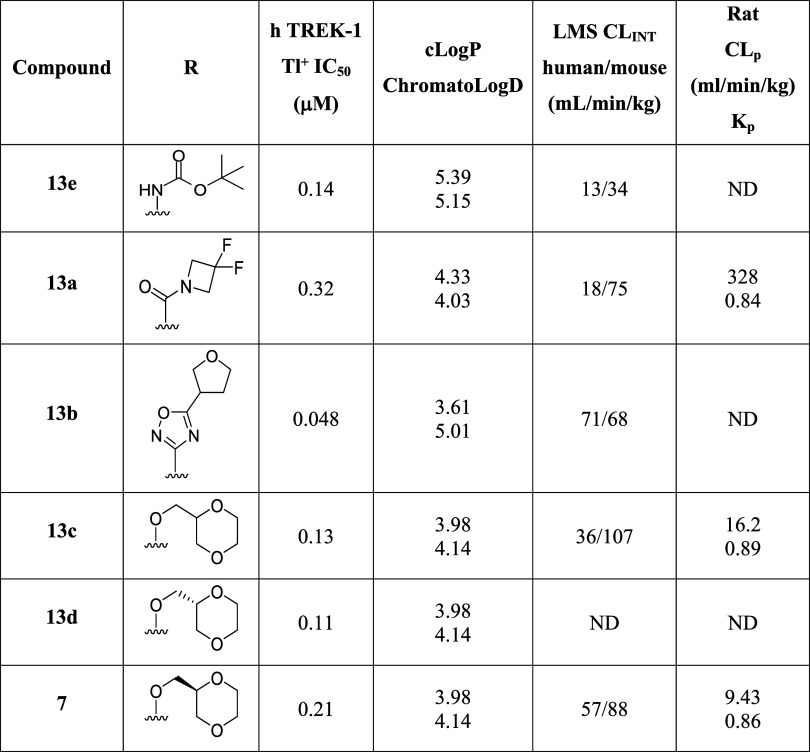
TREK-1 Inhibitory Activity and *In Vitro* and *In Vivo* DMPK Profiles of Analogs **7** and **13** ^a^

a ND = not determined.

A heterocycle survey from the 2-pyridyl analog **6** to regioisomeric pyrazoles and pyrrole **14**, **8**, and **9** proved highly intriguing (Table 2). Maintaining the dioxane of **13c** in the context of a 3-chloro-1-(1,4-dioxan-2-yl)­methyl-1*H*-pyrazolo-4-yl core, afforded **14a**, a weak TREK-1 activator (Tl^+^ −28% at 30 μM). Conversion of **14a** into **9**, a 4-chloro-1-(1,4-dioxan-2-yl)­methyl-1*H*-pyrazolo-3-yl analog, produced a more potent TREK-1 activator (Tl^+^ −99% at 30 μM). Evaluation of the remaining regioisomeric congener **8**, a 4-chloro-1-methyl-1*H*-pyrazolo-5-yl, led to TREK-1 inhibition (TREK-1 Tl^+^ IC_50_ = 0.67 μM). The orientation and projection of the dioxolane moiety had a profound, and unexpected, effect on the mode of TREK-1 activity. Replacement of the dioxolane of **8** with a pyran, as in derivative **14b**, afforded a more potent TREK-1 inhibitor (TREK-1 Tl^+^ IC_50_ = 0.20 μM). Finally, a pyrrole analog of **14c** lost considerable TREK-1 potency (TREK-1 Tl^+^ IC_50_ = 1.15 μM), but remained an inhibitor. From this SAR, the team wanted to explore what other moieties would be tolerated as *N*-substituents on the pyrazole ring.

**Table 2:**
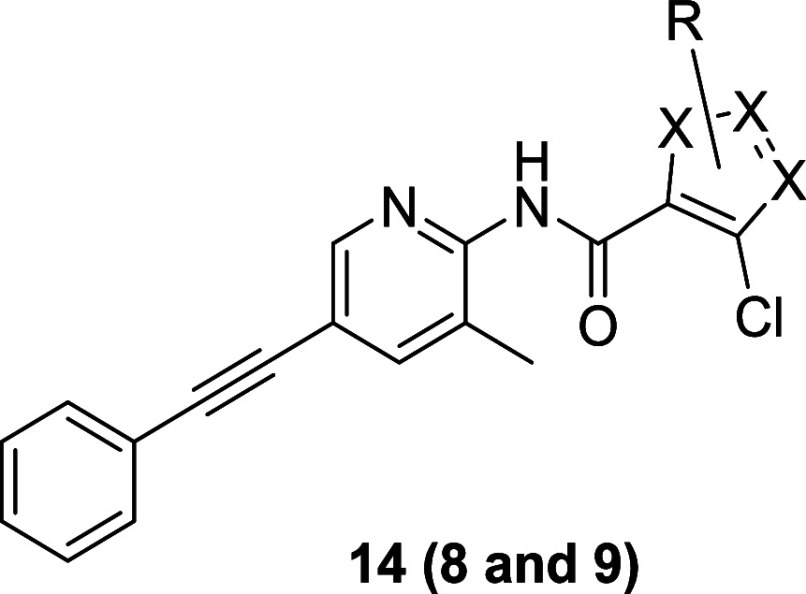

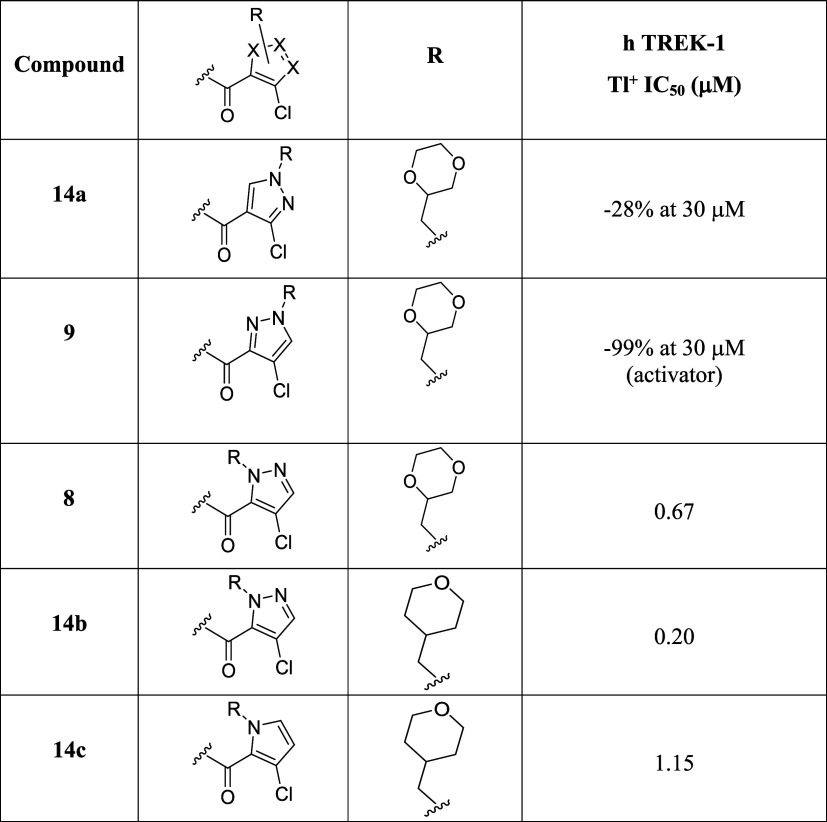
TREK-1 Inhibitory Activity Profile of Analogs **8**, **9**, and **14**

Ring contraction to a furan led to the pair of enantiomers **15a** and **15b** (Table 3). Here, the (*S*)-enantiomer **15a** was 2-fold more potent (TREK-1 Tl^+^ IC_50_ = 0.36 μM) than the (*R*)-enantiomer **15b** (TREK-1 Tl^+^ IC_50_ = 0.75 μM). Moreover, **15a** displayed low predicted intrinsic clearance, modest protein binding (∼98% in rat and mouse) and showed low clearance *in vivo* (rat CL_p_ = 8.9 mL/min/kg) with good CNS penetration (rat *K*_p_ = 2.8). Transitioning from sp^3^ to sp^2^ chemical space with the *N*-methyl indazole derivative **15c** increased potency (TREK-1 Tl^+^ IC_50_ = 0.084 μM) but at the cost of high plasma protein binding (rat, 99.8%). An aliphatic cyano analog **15d** was potent (TREK-1 Tl^+^ IC_50_ = 0.27 μM), displayed low predicted intrinsic clearance in human and rat, but was highly protein bound (mouse, 99.9%). A fluorinated azetidinyl acetamide **15e** was potent (TREK-1 Tl^+^ IC_50_ = 0.33 μM) but demonstrated high predicted hepatic clearance yet favorable protein binding (human, mouse: 96.1%, 97.8%, respectively). Ring expansion of **15e** to the piperidinyl acetamide **10** afforded an attractive compound. TREK-1 inhibitor **10** was potent (TREK-1 Tl^+^ IC_50_ = 0.067 μM), with low predicted intrinsic clearance in human and mouse, modest protein binding, and favorable *in vivo* rat PK (CL_p_ = 27.7 mL/min/kg) and CNS penetration (rat *K*_p_ = 2.7). An exocyclic, *cis*-cyclohexylcongener **15f** was also potent (TREK-1 Tl^+^ IC_50_ = 0.13 μM) but possessed high predicted intrinsic clearance, acceptable protein binding, and favorable *in vivo* rat PK (CL_p_ = 27.8 mL/min/kg) and CNS penetration (rat *K*_p_ = 0.97). Finally, an octahydrocyclopenta­[*c*]­pyrrole isostere **15g** of **10** was ∼4-fold less potent (TREK-1 Tl^+^ IC_50_ = 0.26 μM) and showed higher protein binding. From this SAR exploration, **10** emerged as a compound worthy of further profiling, as well as additional, targeted SAR around **10**.

**Table 3:**
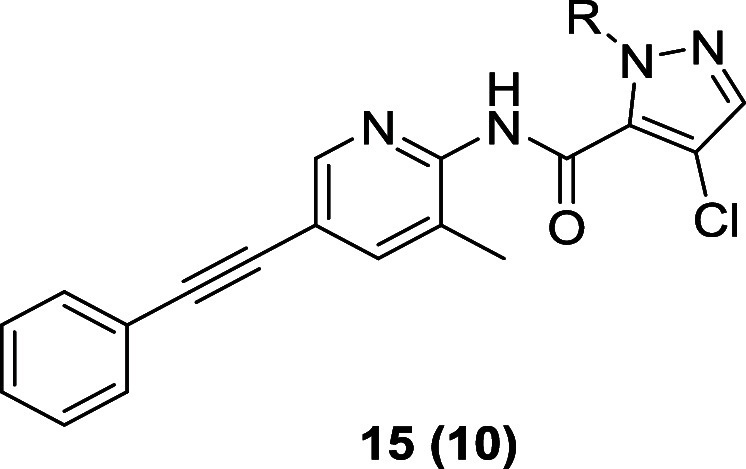

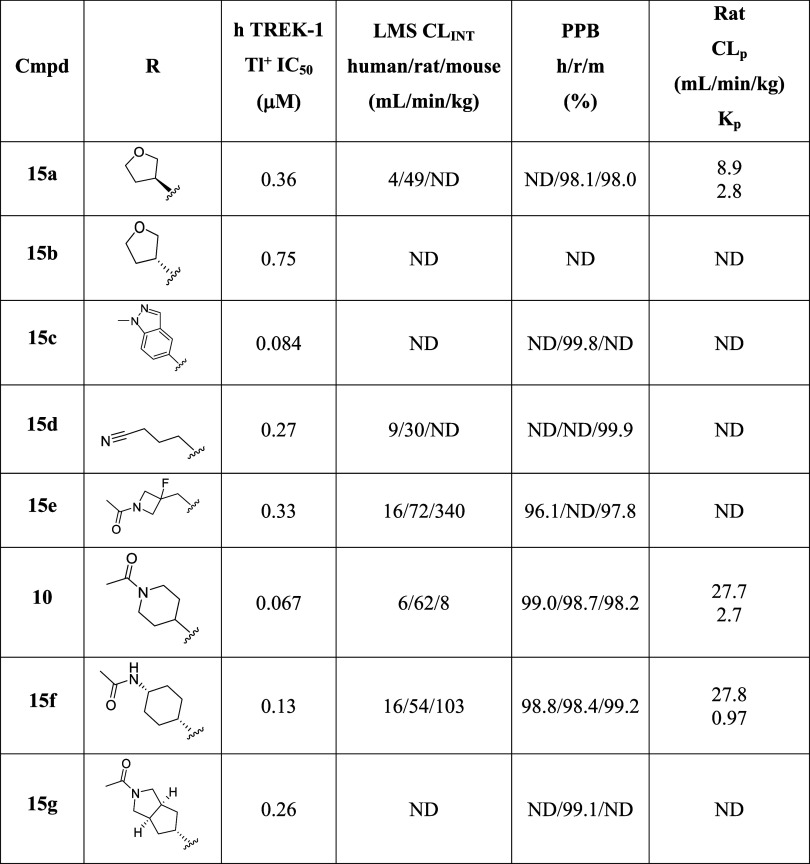
TREK-1 Inhibitory Activity and *In Vitro* and *In Vivo* DMPK Profiles of Analogs **10** and **15** ^a^

a ND = not determined.

For the SAR of **10** (Table 4), we introduced a fluorine atom into the 3-position of the piperidine ring and evaluated substituents on both the pyridine core (R^1^) and the distal phenyl ring (R^2^) of the acetylene, as we previously demonstrated that a fluorine atom at R^2^ can improve disposition. The direct 3-fluoro congener of **10**, **16a** (a mixture of four diastereomers), was a potent inhibitor of both TREK-1 (TREK-1 Tl^+^ IC_50_ = 0.098 μM) and TREK-2 (TREK-2 Tl^+^ IC_50_ = 0.28 μM); thus, like **5**, compounds here are dual TREK-1/2 inhibitors. Resolution of the diastereomers and assessing three of the four possible diastereomers **16b**–**16d**, indicated that (3*R*,4*R*) diastereomer **16c** was ∼2-fold more potent than (3*S*,4*R*) and (3*R*,4*S*) diastereomers at both TREK-1 and TREK-2. Replacement of the methyl at R^1^ with a fluorine afforded enantiomers **16e** and **16f**, wherein the (3*R*,4*R*) enantiomer **16e** was more potent (TREK-1 Tl^+^ IC_50_ = 0.07 μM, TREK-2 Tl^+^ IC_50_ = 0.33 μM) than the (3*S*,4*S*) enantiomer **16f**. Finally, the addition of a fluorine atom to the 4-position of the distal phenyl ring led to **11**, the (3*R*,4*R*) diastereomer. Compound **11** was a potent TREK-1 (TREK-1 Tl^+^ IC_50_ = 0.11 μM) and TREK-2 (TREK-2 Tl^+^ IC_50_ = 0.29 μM) inhibitor with low predicted intrinsic clearance for both human and rat. With **10** and **11** in hand, all efforts centered on deep profiling of these two TREK inhibitors toward the identification and selection of a preclinical research tool for exploring selective TREK inhibition *in vitro* and *in vivo*.

**Table 4:**
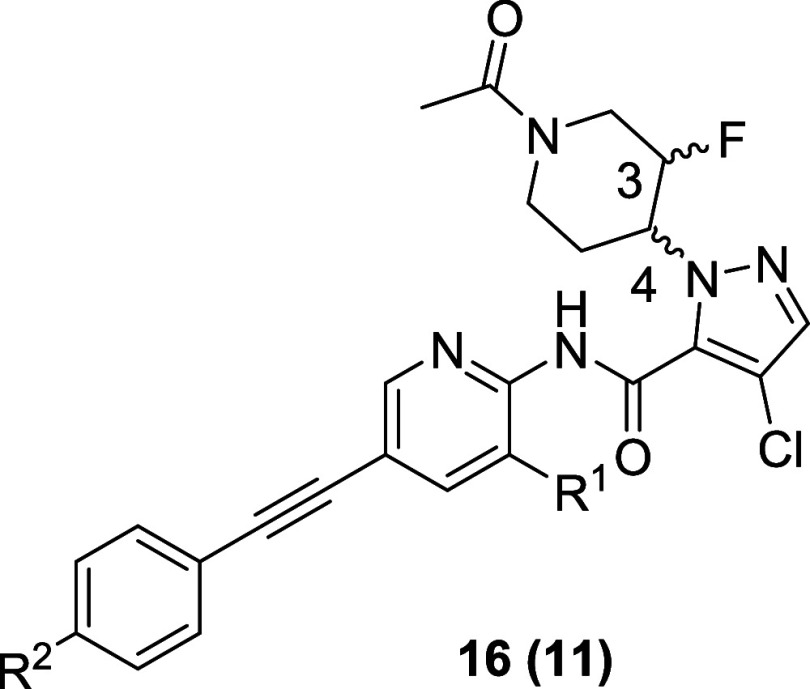
TREK-1/2 Inhibitory Activity and *In Vitro* DMPK Profiles of Analogs **11** and **16** ^a^

cmpd	R^1^	R2	stereochemistry	h TREK-1 Tl^+^ IC_50_ (μM)	h TREK-2 Tl^+^ IC_50_ (μM)	LMS CL_INT_ human/rat/mouse (mL/min/kg)
**16a**	Me	H	mixture of 4 diastereomers	0.098	0.28	ND
**16b**	Me	H	(3*R*,4*R*)	0.096	0.29	13/94/ND
**16c**	Me	H	(3*S*,4*R*)	0.16	0.53	ND
**16d**	Me	H	(3*R*,4*S*)	0.18	0.53	ND
**16e**	F	H	(3*R*,4*R*)	0.07	0.33	ND
**16f**	F	H	(3*S*,4*S*)	0.24	0.47	ND
**11**	Me	F	(3*R*,4*R*)	0.11	0.29	10/40/134

a ND = not determined.

### Detailed Profiling of **10** and **11**

The detailed pharmacology and *in vitro* DMPK profiles for **10** (ONO-9517601/VU6022856) and **11** (ONO-7927846/VU6024391) are shown in Table 5. Both compounds are under 500 molecular weight, ChromatoLogD values below 4, tPSAs ∼80 and acceptable aqueous solubility (31 μM and 14 μM for **10** and **11**, respectively). Compound **10** is a potent TREK-1 inhibitor in both the human thallium flux assay (TREK-1 IC_50_ = 0.067 μM) and human manual patch clamp (TREK-1 IC_50_ = 0.063 μM), as well as a TREK-2 inhibitor (human thallium TREK-2 IC_50_ = 0.23 μM, human manual patch clamp TREK-2 IC_50_ = 0.081 μM). TREK-1 activity is also maintained across rodent species for **10** (rat TREK-1 thallium IC_50_ = 0.28 μM, rat MPC IC_50_ = 0.088 μM; mouse TREK-1 thallium IC_50_ = 0.23 μM, mouse MPC IC_50_ = 0.067 μM). Compound **10** displayed low predicted hepatic clearance for both human (4.9 mL/min/kg) and rat (32.9 mL/min/kg) and relatively high plasma protein binding in human (99.0%) and rat (98.7%) as well as in brain homogenate binding for both rat (99.5%) and mouse (99.5%). The CYP_450_ profile was acceptable IC_50_s for 3A4 (10.8 μM), 2C9 (6.1 μM), 2D6 (10.3 μM), and 1A2 (23.6 μM) and **10** was negative in the AMES assay (5-strains, with and without S9). CACO-2 ER was 0.70 and rat *in vivo* CNS penetration was excellent (rat *K*_p_ = 2.7; *K*_p,uu_ = 1.0).

**Table 5:**
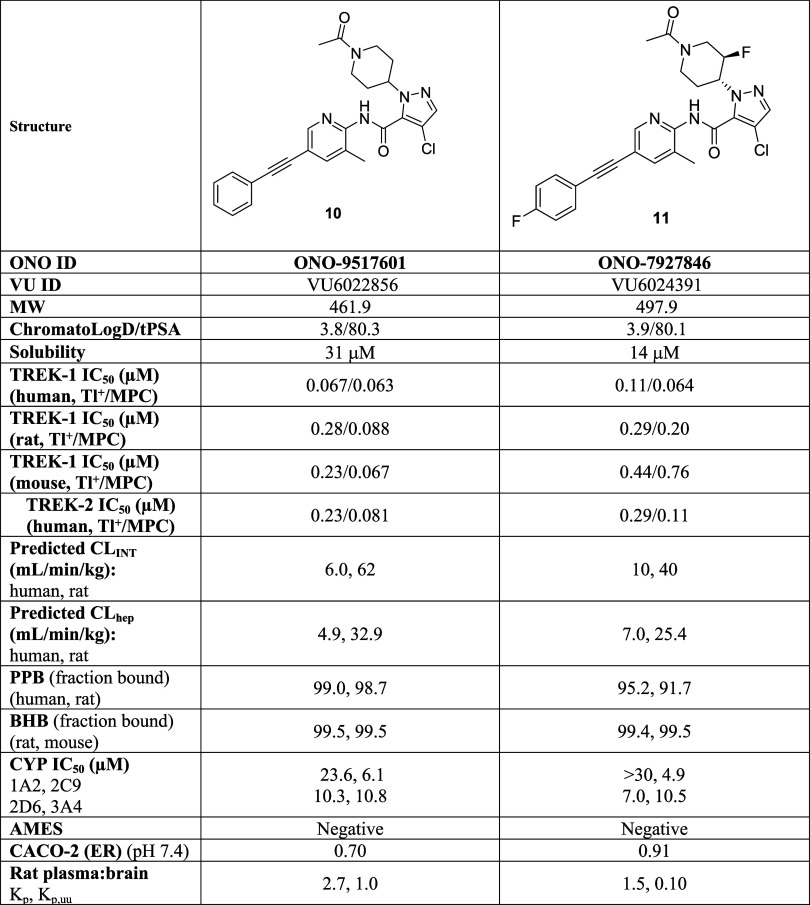
Detailed Profiles of TREK Inhibitors **10** and **11**

Compound **11** is also a potent TREK-1 inhibitor in both the human thallium flux assay (TREK-1 IC_50_ = 0.11 μM) and human manual patch clamp (TREK-1 IC_50_ = 0.064 μM), as well as comparably potent TREK-2 inhibitor (human thallium TREK-2 IC_50_ = 0.29 μM, human manual patch clamp TREK-2 IC_50_ = 0.11 μM). TREK-1 activity is also maintained across rat for **11** (rat TREK-1 thallium IC_50_ = 0.29 μM, rat MPC IC_50_ = 0.20 μM), but weaker at mouse TREK-1 (mouse thallium IC_50_ = 0.44 μM, mouse MPC IC_50_ = 0.76 μM). Compound **11** displayed low predicted hepatic clearance for both human (7.0 mL/min/kg) and rat (25.4 mL/min/kg) and relatively moderate plasma protein binding in human (95.2%) and rat (91.7%), but higher brain homogenate binding for both rat (99.4%) and mouse (99.5%). The CYP_450_ profile was acceptable IC_50_s for 3A4 (10.5 μM), 2C9 (4.9 μM), 2D6 (7.0 μM), and 1A2 (>30 μM), and **11** was negative in the AMES assay (5-strains, with and without S9). CACO-2 ER was 0.91 and rat *in vivo* CNS penetration was excellent (rat *K*_p_ = 1.5; *K*_p,uu_ = 0.1, driven by difference in plasma PPB and BHB).

Both **10** and **11** were evaluated in multispecies IV/PO pharmacokinetic studies (Table 6). Both compounds are generally characterized as low clearance compounds with volume of distributions of 2.1–7.1 L/kg, good half-lives, and excellent oral bioavailability. For **10**, rat PK is good (CL_p_ = 30.0 mL/min/kg, *V*_ss_ = 2.2 L/kg, *t*_1/2_ = 1.0 h, %*F* = 40), NHP PK is attractive (CL_p_ = 13.3 mL/min/kg, *V*_ss_ = 2.1 L/kg, *t*_1/2_ = 3.3 h, %*F* = 34) while dog PK is extended (CL_p_ = 1.9 mL/min/kg, *V*_ss_ = 5.9 L/kg, *t*_1/2_ = 56 h, %*F* = 97). For **11**, rat PK is good (CL_p_ = 6.1 mL/min/kg, *V*_ss_ = 2.7 L/kg, *t*_1/2_ = 5.5 h, %*F* = 57), NHP PK is attractive (CL_p_ = 6.5 mL/min/kg, *V*_ss_ = 4.4 L/kg, *t*_1/2_ = 9.3 h, %*F* = 89) while dog PK is extended (CL_p_ = 1.5 mL/min/kg, *V*_ss_ = 7.1 L/kg, *t*_1/2_ = 76 h, %*F* = 100). For both **10** and **11**, dog is an outlier with exceptionally low clearance (<2 mL/min/kg) and long half-lives (*t*_1/2_ s of 56 to 76 h). Metabolite identification studies are underway to attempt to understand the discrepancies in dog, as well as exploring the potential of metabolic enzymes acting on **10** and **11** not present in dog, e.g, AO/XO. Nevertheless, the overall PK profiles for **11** are superior to **10**.

**Table 6:** *In Vivo* Pharmacokinetic Profiles of TREK Inhibitors **10** and **11**

compound		**10**	**10**	**10**	**11**	**11**	**11**
parameter		rat (SD)	dog (beagle)	NHP (cyno)	rat (SD)	dog (beagle)	NHP (cyno)
dose (mg/kg) iv/po		1/1	1/1	1/1	1/1	0.5/1	1/1
iv	CL_p_ (mL/min/kg)	30.0	1.9	13.3	6.1	1.5	6.5
	*V*_ss_ (L/kg)	2.2	5.9	2.1	2.7	7.1	4.4
	elimination *t*_1/2_ (h)	1.0	56	3.3	5.5	76	9.3
po	*C*_max_ (ng/mL)	80	315	152	162	263	260
	*F* (%) po	40	97	34	57	100	89

### Ancillary Pharmacology

Prior to conducting *in vivo* efficacy studies, we assessed the ancillary pharmacology profiles of **10** and **11** in both a Eurofins Lead Profiling Panel of 68 GPCRs, ion channels, and transporters in radioligand binding displacement assays at 10 μM drug concentration, as well as against a panel of human ion channel using manual patch clamp at a 10 μM drug concentration. For **10**, only two targets displayed >50% inhibition at 10 μM, adenosine A3 (98%) and dopamine transporter DAT (78%). Follow-up work demonstrated binding IC_50_s of 0.38 μM and 2.86 μM for A3 and DAT, respectively; however, in functional assays, the IC_50_ for A3 was 5.37 μM and for DAT was 1.21 μM. In a 10 μM MPC ion channel panel, **10** was inactive or did not display >50% inhibition at 10 μM on Ca_v_1.2, HCN4, K_ir_2.1, K_v_1.5, K_v_4.3, KCNQ1/E1 and Na_v_1.5. The only hit was Kir2.2 at 51% at 10 μM. For **11**, only two targets displayed >50% inhibition at 10 μM, adenosine A3 (91%) and sodium channel site 2 (56%). Follow-up work demonstrated binding IC_50_s of 0.77 μM and 9.13 μM for A3 and sodium channel site 2, respectively; however, in functional assay, the IC_50_ for A3 was 19.8 μM. In a 10 μM MPC ion channel panel, **11** was inactive or did not display >50% inhibition at 10 μM on Ca_v_1.2, HCN4, K_ir_2.1, K_v_1.5, K_v_4.3, Kir2.2 and Na_v_1.5. The only hit was KCNQ1/E1 at 60% at 10 μM. Within the K_2_P family, selectivity of **10** was similar to **5**, with inhibition noted for TRAAK (84% at 10 μM) and TASK-3 (78% at 10 μM), and nothing significant for TASK-1, TASK-2, THIK-1 and TRESK. Both **10** and **11** displayed functional hERG inhibition, with IC_50_s of 6.7 μM and 4.3 μM, respectively. Here, further advancement would depend on safety margins.

### Novel Object Recognition

The primary pharmacodynamic (PD) assay for this program was the novel object recognition (NOR) cognition assay in rat.^25^ Previously, our team showed that **5** was efficacious in the MK-801 NOR challenge assay with a minimum effective dose (MED) of 10 mg/kg IP to mouse. In this paradigm, a 0.2 mg/kg IP injection of MK-801 induces a profound deficit, and both **10** and **11**, dosed orally, significantly reversed the MK-801 deficit at 1, 3 mg/kg and 0.3, 1 mg/kg respectively (Figure 4). The doses were based on rat PK, rat PPB and CNS exposure differences for the two compounds. For **10**, the MED was 1 mg/kg and the MED for **11** was 0.3 mg/kg. Also, when TREK inhibitors **10** and **11** were dosed orally to the MK-801 challenged mouse, the MED in the NOR cognition assay was 1 and 3 mg/kg, respectively (data not shown). Thus, selective inhibition of TREK-1/2 has been demonstrated with three compounds to be efficacious in the NOR paradigm and suggests therapeutic relevance for TREK-1 and or TREK-1/2 inhibition to treat cognitive disorders. *C*_max_ [brain] at the MED for **10** was 218 nM at 1 mg/kg, and for **11**, the *C*_max_ [brain] 58 nM at 0.3 mg/kg.

**Figure 4:**
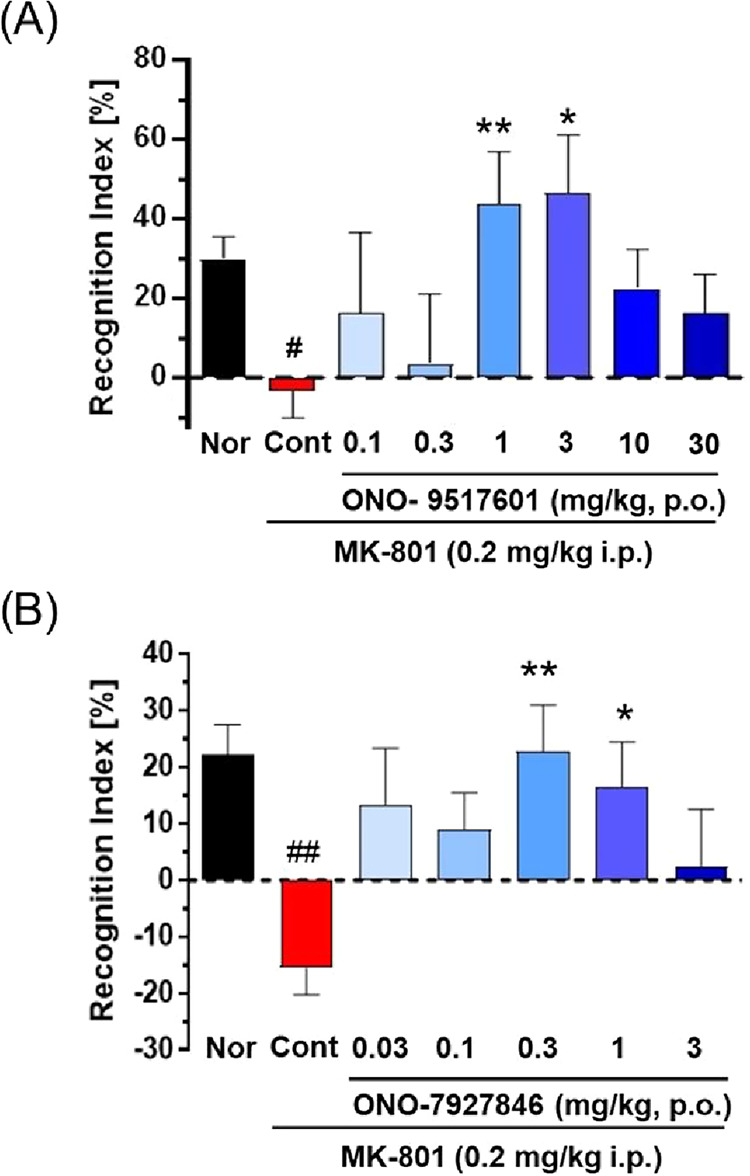
Effect of **10** (ONO-9517601) and **11** (ONO-7927846) in the MK-801 challenged novel object recognition task. Both **10** and **11** enhanced recognition memory in male rats after challenged with MK-801. (A) Pretreatment with 0.1, 0.3, 1, 3, 10, and 30 mg/kg **10** PO 3.5 h prior to MK-801 treatment and exposure to identical objects significantly enhanced recognition memory assessed 90 min later. Minimum effective dose (MED) is 1 mg/kg. (B) Pretreatment with 0.03, 0.1, 0.3, 1, and 3 mg/kg **11** PO 3.5 h prior to MK-801 treatment and exposure to identical objects significantly enhanced recognition memory assessed 90 min later. Minimum effective dose (MED) is 0.3 mg/kg. Mean ± SEM, *n* = 8–14, #*p* < 0.05, ##*p* < 0.01 vs normal (*t* test), *p < 0.05, **p < 0.01 vs MK-801 (Dunnett’s test).

### Toxicology

To assess the potential for further development of **10** and **11**, we performed a battery of preclinical toxicology studies with scaled up material. First, we performed a modified Irwin neurological test battery in rats at 56.6 mg/kg PO (10% Tween 80 in water) and found that there were no significant alterations in any autonomic or somatomotor nervous system functions. Next, we assessed the general toxicity in a rat 4-day dosing study. The NOAEL for **10** was found to be 10 mg/kg PO, whereas the NOAEL for **11** was found to be 6 mg/kg PO. The safety margin of **10** and **11** was both more than 10-fold based on the AUC at those doses. The last checkpoint to evaluate was embryo fetal developmental toxicity *in vivo*. Unfortunately, **10** and **11** tested positive in this assay, and the safety margins proved too narrow to further advance.

### Chemistry

The general synthetic route to access compounds **5**-**16** is depicted in Scheme 1. Starting from commercially available trisubstituted pyridine **17**, a Sonogashira coupling with the appropriately functionalized phenyl acetylene provides **18** in yields ranging from 68 to 97%. An amide coupling reaction with either PyClU or TCFH and the appropriate aryl or heteroarylcarboxylic acid provides analogs **5**-**16** in yields ranging from 6–75%. The optimized synthesis of **11** (Scheme 2) required only 1 + 5 steps and **11** was obtained as a white powder (16.7 g, 44% yield over 5 steps from **20**). A Sonogashira coupling between iodo-pyridine **17a** and 4-fluorophenyl acetylene provides **19** in 92% yield. Commercial pyrazole ester **20** is subjected to a Mitsunobu reaction with chiral fluoro alcohol **21** facilitated by cyanomethylenetributylphosphorane (CMBP) or the Tsunoda reagent, and after saponification of the ester delivers carboxylic acid **22** in 78% yield for the two step sequence. Then, a TCFH (*N*,*N*,*N*′,*N*′-tetramethylchloroformamidinium hexafluorophosphate) mediated amide coupling between **19** and **22** affords **23** in 68% yield. The *N*-Boc protecting group is then removed with methane sulfonic acid in 95% yield. Acylation of the secondary amine to **11** followed by recrystallization from methanol:water provides **11** in 87% yield.

**Scheme 1:**
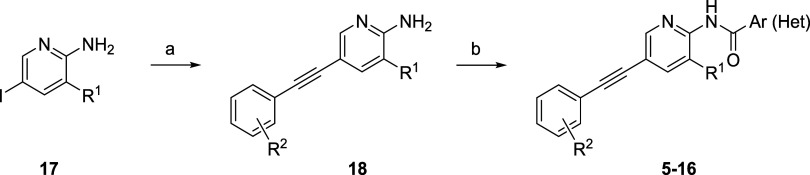
Synthesis of Compounds **5**–**16** ^a^

a Reagents and conditions: (a) PdCl_2_(PPh_3_)_2_ (10 mol %), CuI (10 mol %), DIEA (7 equiv), DMF, 60 °C, 2 h, 68 to 97%; (b) PYCLU (2 equiv), Ar­(Het)-CO_2_H (1.5 equiv), pyridine or DIEA, DCE, rt -50 °C or TCFH (1.5 equiv), NMM (2.0 equiv), NMP (10 v/w), rt, 2h, 6–75%.

**Scheme 2:**
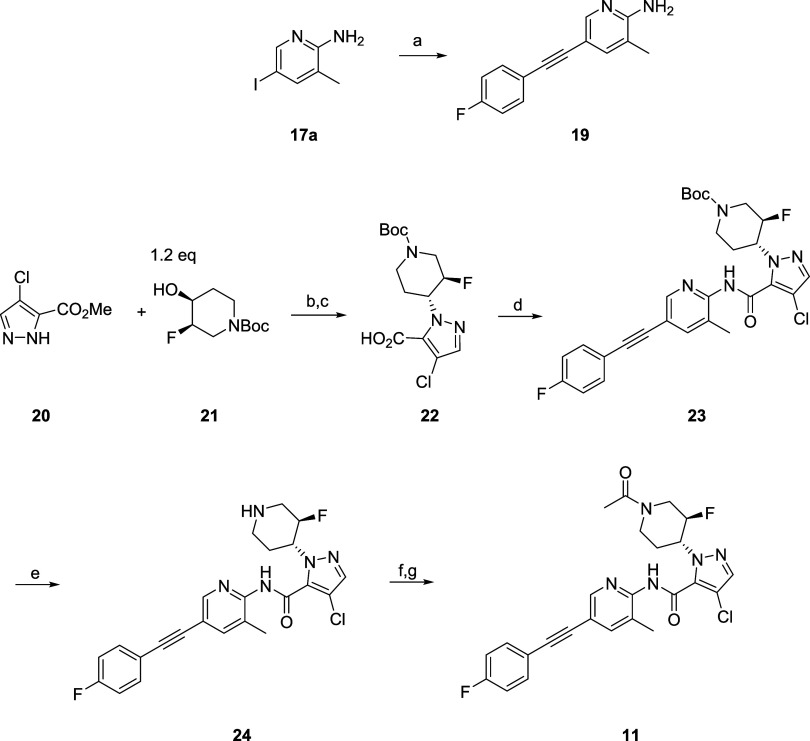
Synthesis of Compounds **11** ^a^

a Reagents and conditions: (a) 4-fluorophenylacetylene (1.5 equiv), PdCl_2_(PPh_3_)_2_ (3 mol %), CuI (1.5 mol %), TEA (2 equiv), DMA (4.3 v/w), 50 °C, 1 h, 92%; (b) CMBP (1.5 equiv), toluene (10 v/w), 90 °C, 7.5 h; (c) NaOH aq. (2.5 equiv), THF/MeOH (7.3 v/w), rt, 4 h, 78% in 2 steps; (d) **19** (1.1 equiv), TCFH (1.5 equiv), NMM (2.0 equiv) NMP (10 v/w), rt, 2.5 h, 68%; (e) MsOH (4 equiv), MeOH (7.5 v/w), 55 °C, 2h, 95%; (f) Ac_2_O (1.2 equiv), pyridine (1.5 equiv), MeCN (10 v/w), 15 °C to rt, 1 h; (g) recrystallization MeOH/H_2_O (2/1, 50 v/w), 87%.

## Conclusions

In summary, we disclosed the chemical optimization of a novel series of TREK inhibitors based on our first-generation CNS tool compound ONO-TR-772. Optimization of ONO-TR-772 focused on replacements for the *N*-Boc aniline moiety and identified *N*-acyl piperidine pyrazoles as attractive surrogates, affording excellent potency, PK profiles, CNS penetration and ion channel selectivity. This effort resulted in the identification of two valuable preclinical research tools, ONO-9517601 (VU6022856) and ONO-7927846 (VU6024391) for exploring selective TREK inhibition *in vitro* and *in vivo*. In an MK-801 NOR challenge assay, both displayed efficacy with MEDs of 1 mg/kg (ONO-9517601) and 0.3 mg/kg (ONO-7927846). Safety margins from four-day toxicology studies in rats supported the further development of ONO-9517601 and ONO-7927846; however, both afforded a positive signal for embryo fetal developmental toxicity, and further development was halted. Nevertheless, ONO-7927846 can serve as a best-in-class *in vivo* rodent tool compound to study selective TREK inhibition.

## Experimental Section

### General Chemistry

All reactions were carried out employing standard chemical techniques under an inert atmosphere. Solvents used for extraction, washing, and chromatography were HPLC grade. All reagents were purchased from commercial sources and were used without further purification. All microwave reactions were carried out in sealed tubes in a Biotage Initiator microwave synthesis reactor. Temperature control was automated via IR sensor and all indicated temperatures correspond to the maximal temperature reached during each experiment. Analytical HPLC method (i) was performed on an Agilent 1200LCMS with UV detection at 215 and 254 nm along with ELSD detection and electrospray ionization, with all final compounds showing >95% purity and a parent mass ion consistent with the desired structure. Low resolution mass spectra were obtained on an Agilent 6120 or 6150 with ESI source. Reversed-phase LCMS analysis (method (ii)) was obtained on a SHIMADZU LCMS-2020 with ESI source. MS parameters were as follows: Mobile Phase: 0.1% TFA in water (solvent A) and 0.1% TFA in acetonitrile (solvent B), using the elution holding at 5% (solvent B) for 0.1 min, gradient 5%–95% (solvent B) over 1.1 min and holding at 95% for 0.4 min at a flow rate of 1.0 mL/min; Column: YMC Triart C18 Φ2.0 mm × L30 mm; Wavelength: UV 220 nm, 254 nm; Column temperature: 30 °C; detector MS, ELSD; MS ionization: ESI. Method (i) was used unless specified. All NMR spectra were recorded on a 400 MHz Brüker AV-400 instrument or a 600 MHz VNS600 Agilent NMR spectrometer. ^1^H chemical shifts are reported as δ values in ppm relative to the residual solvent peak (CDCl_3_ = 7.26). Data are reported as follows: chemical shift, multiplicity (br. = broad, s = singlet, d = doublet, t = triplet, q = quartet, dd = doublet of doublets, m = multiplet), coupling constant (Hz), and integration. ^13^C chemical shifts are reported as δ values in ppm relative to the residual solvent peak (CDCl_3_ = 77.16). High resolution mass spectra were obtained on a Waters Corporation SYNAPT G2-Si with ESI source. MS parameters were as follows: Capillary: 2.0 kV, Sampling Cone: 10, Source Offset: 50, Source: 120 °C, Desolvation: 400 °C, Gas Flow Cone Gas: 50 L/h, Desolvation Gas: 1000 L/h, Nebuliser Gas: 6.5 bar. Samples were introduced via an Waters ACQUITY UPLC I-Class. UV absorption was observed at DAD (210–400 nm). Column: YMC-Triart C18, S-1.9 μm, 12 nm, 30 mm × 2.0 mm I.D. Gradient conditions: 5% to 95% CH_3_CN (0.1% formic acid) over 10 min, 0.3 mL/min, 40 °C. Automated flash column chromatography was performed on a Teledyne ISCO Combiflash Rf system. For compounds that were purified on a Gilson preparative reversed-phase HPLC, the system comprised of a 333 aqueous pump with solvent-selection valve, 334 organic pump, GX-271 or GX-281 liquid hander, two column switching valves, and a 155 UV detector. UV wavelength for fraction collection was user-defined, with absorbance at 254 nm always monitored. Method: Phenomenex Axia-packed Luna C18, 30 mm × 50 mm, 5 μm column. Mobile phase: CH_3_CN in H_2_O (0.1% TFA). Gradient conditions: 0.75 min equilibration, followed by user-defined gradient (starting organic percentage, ending organic percentage, duration), hold at 95% CH_3_CN in H_2_O (0.1%TFA) for 1 min, 50 mL/min, 23 °C. Melting points were recorded on an OptiMelt automated melting point system by Stanford Research Systems. cLogP, MW, and TPSA were calculated using PerkinElmer ChemDraw professional version 20.1.0.110. All final compounds were purified to >95% as determined by analytical LCMS (214 nm, 254 nm, and ELSD), ^1^H and/or ^13^C NMR, and high-resolution MS. Synthesis and/or characterization of intermediates as well as remaining final compounds are in the Supporting Information (SI).

#### 2-Chloro-5-(3,3-difluoroazetidine-1-carbonyl)-*N*-[3-methyl-5-(2-phenylethynyl)-2-pyridyl]­benzamide (**13a**)

A solution of HATU (49 mg, 0.13 mmol), 3,3-difluoroazetidine hydrochloride (8 mg, 0.06 mmol), **13g** (25 mg, 0.06 mmol), and DIEA (0.03 mL, 0.19 mmol) in DMF (1.9 mL) was heated to 50 °C. After 12 h, the reaction mixture was purified by reverse phase HPLC (gradient: 20–60% MeCN in water (0.1% TFA in water)). Fractions that contained the desired product were combined and diluted with water and NaHCO_3_ solution. The reaction mixture was extracted with EtOAc (2 × 10 mL). The combined organic layers were concentrated to yield **compound 16** (10 mg, 34% yield), ES-MS [M + H]^+^: 466.0; LCMS Retention time: 0.96 min; ^1^H NMR (400 MHz, CDCl_3_) δ 8.94 (br s, 1H), 8.32 (s, 1H), 8.02 (d, *J* = 1.9 Hz, 1H), 7.79 (d, *J* = 1.3 Hz, 1H), 7.73 (dd, *J* = 8.3, 2.1 Hz, 1H), 7.56–7.53 (m, 3H), 7.40–7.36 (m, 3H), 4.56 (t, *J* = 11.7 Hz, 4H), 2.41 (s, 3H).

#### 2-Chloro-*N*-(3-methyl-5-(phenylethynyl)­pyridin-2-yl)-5-(5-(tetrahydrofuran-3-yl)-1,2,4-oxadiazol-3-yl)­benzamide (**13b**)

A mixture of Intermediate **13i** (5 mg, 0.01 mmol), tetrahydro-3-furoic acid (0.002 mL, 0.022 mmol), HCTU (9 mg, 0.022 mmol), and DIEA (0.004 mL, 0.004 mmol) in DMF (0.5 mL) was heated at 100 °C. After 17 h, the reaction mixture was filtered, concentrated in vacuo, and purified by reverse phase HPLC (gradient: 50–80% MeCN in water (w/0.05% NH_4_OH)) to give **13b** as an off-white solid (3 mg, 50% yield). ES-MS [M + H]^+^: 485.2; LCMS Retention time: 1.14 min; ^1^H NMR (400 MHz, DMSO-*d*_6_) δ 10.95 (s, 1H), 8.45 (s, 1H), 8.15–8.07 (m, 2H), 7.96 (s, 1H), 7.77 (d, *J* = 8.3 Hz, 1H), 7.63–7.55 (m, 2H), 7.48–7.43 (m, 3H), 5.33 (dd, *J* = 8.0, 5.1 Hz, 1H), 4.10–3.80 (m, 2H), 2.45–2.36 (m, 1H), 2.35–2.33 (m, 3H), 2.33–2.22 (m, 1H), 2.11–1.96 (m, 2H).

#### 2-Chloro-5-(1,4-dioxan-2-ylmethoxy)-*N*-[3-methyl-5-(2-phenylethynyl)-2-pyridyl]­benzamide (**13c**)

A solution of intermediate **13j** (50 mg, 0.14 mmol), 2-(bromomethyl)­dioxane (30 mg, 0.16 mmol), and K_2_CO_3_ (48 mg, 0.34 mmol) in DMF (2.5 mL) was heated to 110 °C in the microwave synthesizer for 10 min. The reaction mixture was purified by reverse phase HPLC (gradient: 30–100% MeCN in water (w/0.05% NH_4_OH)). Fractions that contain product were combined and concentrated to yield **13c** (15 mg, 23% yield), ES-MS [M + H]^+^: 463.0; LCMS Retention time: 0.96 min; ^1^H NMR (400 MHz, CD_3_OD) δ 8.43 (s, 1H), 7.91 (d, *J* = 1.4 Hz, 1H), 7.58–7.54 (m, 2H), 7.43–7.39 (m, 4H), 7.25 (d, *J* = 3.0 Hz, 1H), 7.08 (dd, *J* = 8.9, 3.0 Hz, 1H), 4.09–4.01 (m, 2H), 3.99–3.94 (m, 1H), 3.89 (dd, *J* = 11.5, 2.6 Hz, 1H), 3.85–3.81 (m, 1H), 3.79–3.71 (m, 2H), 3.63 (ddd, *J* = 14.0, 11.0, 3.3 Hz, 1H), 3.54 (dd, *J* = 11.5, 9.9 Hz, 1H), 2.41 (s, 3H).

#### 5-Bromo-2-chloro-*N*-(3-methyl-5-(phenylethynyl)­pyridin-2-yl)­benzamide (**13f**)

To a solution of 5-bromo-2-chlorobenzoic acid (1357 mg, 5.76 mmol), 3-methyl-5-(phenylethynyl)­pyridin-2-amine **18a** (1000 mg, 4.80 mmol), pyridine (1.17 mL, 14.41 mmol) in DCE (9 mL) was added PyClU (2396 mg, 7.20 mmol). The reaction mixture was heated to 50 °C. After 30 min, LCMS indicated product formation. The mixture was poured into NaHCO_3_-*aq* and THF was added to dissolve desired product. The mixture was extracted with ethyl acetate twice. The combined organic layer was washed with water, subsequently NH_4_Cl-*aq*, water, brine and dried over MgSO_4_. The filtrate was concentrated under reduced pressure to give a crude product, which was triturated with mixture of DCM and hexane to yield **13f** as an off-white solid (567 mg, 28% yield). ES-MS [M + H]^+^: 427.0; ^1^H NMR (400 MHz, DMSO-*d*_6_) δ 10.84 (s, 1H), 8.45 (s, 1H), 7.94 (d, *J* = 1.3 Hz, 1H), 7.81 (d, *J* = 2.4 Hz, 1H), 7.59–7.58 (m, 3H), 7.52 (d, *J* = 8.6 Hz, 1H), 7.49–7.42 (m, 3H), 2.32 (s, 3H).

#### 4-Chloro-3-[[3-methyl-5-(2-phenylethynyl)-2-pyridyl]­carbamoyl]­benzoic Acid (**13g**)

To a vessel was added potassium acetate (104 mg, 1.1 mmol), intermediate **13f** (150 mg, 0.35 mmol), and dichloro­[1,1′-bis­(diphenylphosphino)­ferrocene]­palladium­(II) dichloromethane adduct (58 mg, 0.07 mmol). The vessel was sealed and purged with N_2_. To the mixture was added methanol (1.8 mL) and DMF (1.8 mL). An atmosphere of carbon monoxide was applied to the reaction vessel via a balloon. After 16 h at 50 °C, the reaction mixture was purified by reverse phase HPLC (gradient: 10–50% MeCN in water (0.1% TFA in water)). The fractions that contained desired product were combined and diluted with water and NaHCO_3_ aqueous solution. The mixture was extracted with EtOAc (3 × 10 mL) and the combined organic layers were washed with water and concentrated. To the residue was added THF (1.0 mL) and LiOH (1 mol/L, 1 mL, 1 mmol). After 1 h at 40 °C, 2 N HCl was added dropwise to the solution until the pH was ∼7. The mixture was concentrated and the resulting solids were washed with water and collected via vacuum filtration to yield **13g** (67 mg, 49% yield over two steps), ES-MS [M + H]^+^: 391.2; ^1^H NMR (400 MHz, CD_3_OD) δ 8.44 (s, 1H), 8.25 (d, *J* = 1.2 Hz, 1H), 8.05 (dd, *J* = 8.7, 1.5 Hz, 1H), 7.92 (s, 1H), 7.61–7.56 (m, 2H), 7.54 (d, *J* = 8.3 Hz, 1H), 7.45–7.39 (m, 3H), 2.44 (s, 3H).

#### 2-Chloro-5-cyano-*N*-(3-methyl-5-(phenylethynyl)­pyridin-2-yl)­benzamide (**13h**)

Compound **13h** was synthesized using a similar condition described for the synthesis of **13f** as an off-white solid (15 mg, 11% yield). ES-MS [M + H]^+^: 372.2; LCMS Retention time: 1.06 min; ^1^H NMR (400 MHz, DMSO-*d*_6_) δ 10.91 (s, 1H), 8.44 (s, 1H), 8.19 (d, *J* = 2.0 Hz, 1H), 7.99 (dd, *J* = 8.4, 2.1 Hz, 1H), 7.97–7.93 (m, 1H), 7.80 (d, *J* = 8.4 Hz, 1H), 7.62–7.55 (m, 2H), 7.51–7.42 (m, 3H), 2.33 (s, 3H).

#### 2-Chloro-5-(*N*′-hydroxycarbamimidoyl)-*N*-(3-methyl-5-(phenylethynyl)­pyridin-2-yl)­benzamide (**13i**)

A mixture of compound **13h** (20 mg, 0.05 mmol), hydroxylamine hydrochloride (19 mg, 0.27 mmol), and TEA (0.04 mL, 0.27 mmol) in ethanol (0.5 mL) was heated to 150 °C in a microwave synthesizer for 5 min. The sample was cooled to rt, filtered, concentrated *in vacuo*, and purified by reverse phase HPLC (gradient: 25–55% MeCN in water (w/0.1% TFA)) to give **13i** as an off-white solid that was carried forward as a TFA salt (6 mg, 26% yield). ES-MS [M + H]^+^: 405.3; ^1^H NMR (400 MHz, DMSO-*d*_6_) δ 10.98 (s, 1H), 10.87 (s, 1H), 8.46 (s, 1H), 8.82–7.98 (m, 2H), 7.98–7.95 (m, 2H), 7.83 (dd, *J* = 8.5, 2.3 Hz, 1H), 7.75 (d, *J* = 8.5 Hz, 1H), 7.62–7.55 (m, 2H), 7.52–7.41 (m, 3H), 2.34 (s, 3H).

#### 2-Chloro-5-hydroxy-*N*-[3-methyl-5-(2-phenylethynyl)-2-pyridyl]­benzamide (**13j**)

A solution of 2-chloro-5-hydroxybenzoic acid (99 mg, 0.58 mmol) in thionyl chloride (3 mL, 41 mmol) was heated to reflux. After 1 h, the solution was concentrated *in vacuo* and diluted in DCM (4 mL). A separate solution of intermediate **18a** (60 mg, 0.29 mmol) and DIEA (0.05 mL, 0.29 mmol) was added dropwise. After 10 min at rt, to the solution was added a mixture of DIEA (0.15 mL, 0.87 mmol) in DCM dropwise. After 30 min at rt, to the solution was added 2N NaOH solution (2 mL, 4 mmol). After 16 h at rt, the solution was diluted with DCM and water. The pH of the solution was adjusted to pH ∼6 by dropwise addition of 2N HCl solution. The solution was extracted with DCM (3 × 10 mL). The combined organic layers were concentrated *in vacuo*. The residue was purified by reverse phase HPLC (gradient: 25–75% MeCN in water (0.1% TFA in water)). The fractions that contained product were combined and diluted with water and NaHCO_3_ aqueous solution. The mixture was extracted with EtOAc (2×). The organic layers were washed with water (3 × 20 mL) and concentrated *in vacuo* to yield **13j** (50 mg, 47% yield), ES-MS [M + H]^+^: 363.2; ^1^H NMR (400 MHz, CD_3_OD) δ 8.43 (s, 1H), 7.90 (s, 1H), 7.58–7.54 (m, 2H), 7.42–7.39 (m, 3H), 7.31 (dd, *J* = 8.7, 1.9 Hz, 1H), 7.06 (dd, *J* = 2.5, 2.5 Hz, 1H), 6.90 (ddd, *J* = 8.7, 2.6, 2.6 Hz, 1H), 2.41 (s, 3H).

#### (*R*)-5-((1,4-Dioxan-2-yl)­methoxy)-2-chlorobenzoic Acid (**B1**)

A solution of ethyl 2-chloro-5-hydroxybenzoate (884 mg, 4.41 mmol), (*R*)-(1,4-dioxan-2-yl)­methyl 4-methylbenzenesulfonate (1200 mg, 4.41 mmol), and K_2_CO_3_ (2471 mg, 17.6 mmol) in DMF (35 mL) was subjected to microwave irradiation at 115 °C for 45 min. The solution was diluted with water and extracted with EtOAc (1 × 100 mL). The extraction was washed with water (2 × 20 mL) and concentrated *in vacuo*. The residue was dissolved in THF (69 mL), MeOH (27 mL), and NaOH (2900 mg, 70.7 mmol) were added. After 2 h at rt, the solution was diluted with water and EtOAc and treated with 2 N HCl to lower pH to <5. The reaction mixture was extracted with EtOAc (1 × 500 mL). The organic layer was washed with water (3 × 1 L). Each water wash was re-extracted with EtOAc (3 × 250 mL). The combined organic layers were concentrated *in vacuo*. The residue was dissolved in DCM and filtered through a hydrophobic frit to yield the title compound **B1** (1168 mg, 97% yield), which was taken forward without further purification.

#### (*R*)-5-((1,4-Dioxan-2-yl)­methoxy)-2-chloro-*N*-(3-methyl-5-(phenylethynyl)­pyridin-2-yl)­benzamide (**13d**)

Equally divided into 7 vials, was added PyClU (2137 mg, 6.4 mmol), pyridine (1.73 mL, 21.4 mmol), (*R*)-5-((1,4-dioxan-2-yl)­methoxy)-2-chlorobenzoic acid (**B1**) (1168 mg, 4.28 mmol), compound **18a** (1338 mg, 6.4 mmol), and DCE (22 mL). After 1 h at 50 °C, the solution was diluted with water (200 mL), and extracted with DCM (3 × 100 mL). The combined DCM extracts were washed with water (2 × 100 mL), combined, and concentrated *in vacuo*. The residue was purified by reverse phase HPLC (gradient: 30–80% MeCN in water (w/0.1% TFA)). The fractions were partially concentrated *in vacuo* and diluted with NaHCO_3_ aqueous solution (0.5 mL), water, and EtOAc. The sample was extracted with EtOAc (2 × 200 mL). The combined organic layers were washed with water (3 × 100 mL) and concentrated. The resulting solids were sonicated in MeOH, and the resulting slurry was filtered to afford compound **13d** (552 mg, 28% yield). ESI-MS [M + H]^+^: 463.2; LCMS Retention time: 1.15 min (method (ii)); ^1^H NMR (600 MHz, DMSO-*d*_6_) δ 10.72 (s, 1H), 8.46 (br s, 1H), 7.94 (s, 1H), 7.59–7.60 (m, 2H), 7.43–7.49 (m, 4H), 7.16 (d, *J* = 2.8 Hz, 1H), 7.10 (dd, *J* = 8.9, 2.8 Hz, 1H), 4.04 (d, *J* = 5.0 Hz, 2H), 3.81–3.89 (m, 2H), 3.77 (br d, *J* = 12.0 Hz, 1H), 3.61–3.69 (m, 2H), 3.49–3.53 (m, 1H), 3.40–3.43 (m, 1H), 2.33 (s, 3H).

#### (*S*)-5-((1,4-Dioxan-2-yl)­methoxy)-2-chlorobenzoic Acid (**B2**)

A solution of ethyl 2-chloro-5-hydroxybenzoate (2579 mg, 12.9 mmol), (*S*)-(1,4-dioxan-2-yl)­methyl 4-methylbenzenesulfonate (3500 mg, 12.9 mmol), and K_2_CO_3_ (7209 mg, 51.4 mmol) in DMF (103 mL) was subjected to microwave irradiation at 115 °C for 45 min. The solution was diluted with water and extracted with EtOAc (1 × 300 mL). The extraction was washed with water (2 × 50 mL) and concentrated *in vacuo*. The residue was dissolved in THF (200 mL), MeOH (80 mL), and NaOH (8458 mg, 206 mmol) was added. After 2 h at rt, the solution was diluted with water and EtOAc and treated with 2N HCL solution to lower pH to <5. The reaction mixture was extracted with EtOAc (1 × 1 L). The organic layer was washed with water (3 × 1 L). Each water wash was re-extracted with EtOAc (3 × 250 mL). The combined organic layers were concentrated *in vacuo*. The residue was dissolved in DCM and filtered through a hydrophobic frit to yield the title compound **B2** (3500 mg, 99% yield), which was taken forward without further purification.

#### 5-((4-Fluorophenyl)­ethynyl)-3-methylpyridin-2-amine (**19**)

To a mixture of 5-iodo-2-amino-3-picoline **17a** (45 g, 192 mmol) and copper­(I) iodide (549 mg, 2.99 mmol) in DMA (192 mL) was evacuated and purged with N_2_ (×3), then added triethylamine (54.6 mL, 384 mmol), *trans*-dichlorobis­(triphenylphosphine)­palladium­(II) (4.05 g, 5.78 mmol). While adding 4-fluorophenylacetylene (34.6 g, 288 mmol), exothermic reaction was controlled at 50 °C in a water bath, and the mixture was stirred at 50 °C for 1 h. After TLC confirmed the consumption of compound **17a**, the reaction was cooled to rt, poured in a saturated aqueous ammonium chloride solution, extracted with ethyl acetate. The organics were washed with saturated aqueous ammonium chloride solution and brine, dried over sodium sulfate and concentrated *in vacuo* to afford crude product as a brown solid. The sample was purified by normal phase chromatography (gradient: 40–70% EtOAc in hexanes) to yield **19** (40.0 g, 92% yield). ES-MS [M + H]^+^: 227.2; ^1^H NMR (400 MHz, DMSO-*d*_6_) δ 8.00 (d, *J* = 1.2 Hz, 1H), 7.50–7.56 (m, 2H), 7.39 (d, *J* = 1.2 Hz, 1H), 7.24 (m, 2H), 6.21 (s, 2H), 2.05 (s, 3H).

#### 3-Methyl-5-(phenylethynyl)­pyridin-2-amine (**18a**)

Compound **18a** was prepared in a similar manner as Compound **19** but with DIEA (1807 mg, 68% yield). ES-MS [M + H]^+^: 209.4; ^1^H NMR (400 MHz, CDCl_3_) δ 8.15 (s, 1H), 7.51–7.48 (m, 2H), 7.45 (s, 1H), 7.03 (m, 3H), 4.76 (s, 2H), 2.14 (s, 3H).

#### 3-Fluoro-5-(phenylethynyl)­pyridin-2-amine (**18b**)

Compound **18b** is prepared in a similar manner as Compound **19** but with DIEA (26.0 mg, 97% yield). ES-MS [M + H]^+^: 213.2; ^1^H NMR (400 MHz, CDCl_3_) δ 8.07 (s, 1H), 7.51–7.49 (m, 2H), 7.36–7.33 (m, 4H), 4.76 (s, 2H).

#### *tert*-Butyl (5-Chloro-6-((3-methyl-5-(phenylethynyl)­pyridin-2-yl)­carbamoyl)­pyridin-2-yl)­carbamate (**6**)

To a mixture of compound **18a** (46 mg, 0.22 mmol), 6-((*tert*-butoxycarbonyl)­amino)-3-chloropicolinic acid (72 mg, 0.27 mmol) and pyridine (0.05 mL, 0.66 mmol) in DCE (3 mL) was added PyClU (110 mg, 0.33 mmol). After 3 h at 50 °C, the reaction was concentrated. The residue was purified by normal phase column chromatography (gradient: 5–70% EtOAc in Hexanes) to yield **6** (77 mg, 75% yield). ESI-MS [M + H]^+^: 463.2; LCMS Retention time: 1.29 min (method (ii)); ^1^H NMR (600 MHz, DMSO-*d*_6_) δ 10.71 (s, 1H), 10.20 (br s, 1H), 8.45 (br s, 1H), 7.90–8.02 (m, 3H), 7.57–7.61 (m, 2H), 7.44–7.47 (m, 3H), 2.33 (s, 3H), 1.49 (s, 9H).

#### 1-((1,4-Dioxan-2-yl)­methyl)-4-chloro-1*H*-pyrazole-3-carboxylic Acid (**B4**) and 1-((1,4-Dioxan-2-yl)­methyl)-4-chloro-1*H*-pyrazole-5-carboxylic Acid (**B5**)

The mixture of methyl 4-chloro-1*H*-pyrazole-3-carboxylate **20** (50 mg, 0.31 mmol), 2-(bromomethyl)-1,4-dioxane (100 mg, 0.55 mmol), and Cs_2_CO_3_ (200 mg, 0.61 mmol) in DMF (1 mL) was heated to 60 °C. After 5 h, the reaction mixture was filtered and purified by reverse phase HPLC (gradient: 10–70% MeCN in water (w/0.1% TFA)) to afford the methyl ester intermediate. The methyl ester was diluted in MeOH (1 mL) and 2 M NaOH (aq) (0.5 mL) was added. After 2 h at 50 °C, the reaction mixture was removed from the heating source and 6 M HCl (aq) was added to achieve pH ∼3. The reaction mixture was concentrated. The residue was washed with EtOH (3×), passed through a through a filter, and the filtrate was evaporated to afford the desired carboxylic acid **B4** (25 mg, 33% yield) and carboxylic acid **B5** (15 mg, 20% yield). First eluent (**B4**): ESI-MS [M + H]^+^: 247.4; LCMS Retention time: 0.44 min and second eluent (**B5**): ESI-MS [M + H]^+^: 247.2; LCMS Retention time: 0.51 min

#### 1-((1,4-Dioxan-2-yl)­methyl)-4-chloro-*N*-(3-methyl-5-(phenylethynyl)­pyridin-2-yl)-1*H*-pyrazole-3-carboxamide (**9**)

To a mixture of 1-((1,4-dioxan-2-yl)­methyl)-4-chloro-1*H*-pyrazole-3-carboxylic acid **B4** (25 mg, 0.10 mmol), compound **18a** (10 mg, 0.037 mmol) and pyridine (0.03 mL, 0.37 mmol) in DCM (1 mL) was added PYClU (18.3 mg, 0.055 mmol). After 3 h at 45 °C, the reaction mixture was concentrated and purified by reverse phase HPLC (gradient: 20–95% MeCN in water (w/0.1% TFA)). The combined desired fractions were added to EtOAC (15 mL) and washed with NaHCO_3_ (aq) (10 mL), brine, dried over MgSO_4_, filtered and concentrated to provide the title compound **9** (11 mg, 64% yield). ESI-MS [M + H]^+^: 437.2; LCMS Retention time: 1.11 min (method (ii)); ^1^H NMR (600 MHz, DMSO-*d*_6_) δ 10.21 (s, 1H), 8.48 (s, 1H), 8.13 (s, 1H), 7.94 (s, 1H), 7.59–7.61 (m, 2H), 7.44–7.49 (m, 3H), 4.19–4.28 (m, 2H), 3.96–4.00 (m, 1H), 3.75–3.82 (m, 2H), 3.66 (br d, *J* = 11.6 Hz, 1H), 3.56–3.60 (m, 1H), 3.27–3.50 (m, 2H), 2.25 (s, 3H).

#### 1-((1,4-Dioxan-2-yl)­methyl)-4-chloro-*N*-(3-methyl-5-(phenylethynyl)­pyridin-2-yl)-1*H*-pyrazole-5-carboxamide (**8**)

To a mixture of 1-((1,4-dioxan-2-yl)­methyl)-4-chloro-1*H*-pyrazole-5-carboxylic acid **B5** (15 mg, 0.61 mmol), compound **18a** (10 mg, 0.037 mmol) and pyridine (0.03 mL, 0.37 mmol) in DCM (1 mL) was added PYClU (18.3 mg, 0.055 mmol). After 3 h at 45 °C, the reaction mixture was concentrated and purified by reverse phase HPLC (gradient: 20–95% MeCN in water (w/0.1% TFA)). The combined desired fractions were added to EtOAC (15 mL) and washed with NaHCO_3_ (aq) (10 mL), brine, dried over MgSO_4_, filtered and concentrated to provide the title compound **8** (8 mg, 49% yield). ESI-MS [M + H]^+^: 437.2; LCMS Retention time: 1.17 min (method (ii)); ^1^H NMR (600 MHz, DMSO-*d*_6_) δ 10.86 (s, 1H), 8.45 (s, 1H), 7.96 (s, 1H), 7.71 (s, 1H), 7.58–7.61 (m, 2H), 7.45–7.48 (m, 3H), 4.40 (dd, *J* = 14.3, 7.2 Hz, 1H), 4.34 (dd, *J* = 14.3, 4.8 Hz, 1H), 3.85–3.90 (m, 1H), 3.69–3.75 (m, 2H), 3.63 (br d, *J* = 11.2 Hz, 1H), 3.22–3.54 (m, 3H), 2.29 (s, 3H).

#### 4-Chloro-1-((tetrahydro-2*H*-pyran-4-yl)­methyl)-1*H*-pyrazole-5-carboxylic Acid (**B6**)

Compounds **B6** was prepared using a similar procedure described for **B3** but at 80 °C. Compound **B6** was obtained as a clear, colorless amorphous solid that was carried forward as a TFA salt (151 mg, 34% yield over 2 steps). ES-MS [M-^*t*^Bu + 2H]^+^: 245.2; ^1^H NMR (400 MHz, ((CD_3_)_2_SO)) δ 7.72 (s, 1H), 4.38 (d, *J* = 7.2 Hz, 2H), 3.80 (ddd, *J* = 11.4, 4.1, 1.7 Hz, 2H), 3.22 (td, *J* = 11.6, 2.2 Hz, 2H), 2.10–1.97 (m, 1H), 1.37–1.29 (m, 2H), 1.22 (qd, 11.6, 4.5 Hz, 2H).

#### 4-Chloro-*N*-(3-methyl-5-(phenylethynyl)­pyridin-2-yl)-1-((tetrahydro-2*H*-pyran-4-yl)­methyl)-1*H*-pyrazole-5-carboxamide (**14b**)

Compound **14b** was prepared using a similar procedure described for compound **8**. Compound **14b** was obtained as a beige solid (5 mg, 20% yield). ES-MS [M + H]^+^: 435.4; LCMS Retention time: 1.13 min; ^1^H NMR (400 MHz, CDCl_3_) δ 8.66 (br s, 1H), 8.50 (s, 1H), 7.77 (d, *J* = 1.1 Hz, 1H), 7.58–7.51 (m, 3H), 7.41–7.35 (m, 3H), 4.52 (d, *J* = 7.2 Hz, 2H), 3.98–3.90 (m, 2H), 3.33 (ddd, *J* = 11.8, 11.4, 2.9 Hz, 2H), 2.36 (s, 3H), 2.30–2.16 (m, 1H), 1.51–1.37 (m, 4H).

#### 3-Chloro-1-((tetrahydro-2*H*-pyran-4-yl)­methyl)-1*H*-pyrrole-2-carboxylic Acid (**B7**)

A mixture of methyl 3-chloro-1*H*-pyrrole-2-carboxylate (60 mg, 0.38 mmol), 4-(bromomethyl)­tetrahydro-2*H*-pyran (81 mg, 0.45 mmol) and Cs_2_CO_3_ (247 mg, 0.75 mmol) in DMF (2 mL) was subjected to microwave irradiation at 100 °C for 2 h. The reaction mixture was filtered and purified by reverse phase HPLC (gradient: 20–95% MeCN in water (w/0.1% TFA)) to provide the methyl ester intermediate (ESI-MS [M + H]^+^: 258.2; LCMS Retention time: 0.90 min). To the methyl ester intermediate in MeOH (2 mL) was added 2 M NaOH (aq) (1 mL). The mixture was heated to 60 °C. After 3 h, the reaction was cooled to rt and 6 M HCl (aq) was added to achieve pH ∼3. The mixture was concentrated. To the residue was added EtOH (5 mL) and the mixture was filtered. The filtrate was concentrated to afford the title compound **B7** (70 mg, 76% yield over 2 steps). ESI-MS [M + H]^+^: 244.3; LCMS Retention time: 0.77 min.

#### 3-Chloro-*N*-(3-methyl-5-(phenylethynyl)­pyridin-2-yl)-1-((tetrahydro-2*H*-pyran-4-yl)­methyl)-1*H*-pyrrole-2-carboxamide (**14c**)

To a mixture of 3-chloro-1-((tetrahydro-2*H*-pyran-4-yl)­methyl)-1*H*-pyrrole-2-carboxylic acid **B7** (15 mg, 0.061 mmol), compound **18a** (19.2 mg, 0.092 mmol) and pyridine (0.05 mL, 0.62 mmol) in DCE (1 mL) was added PYClU (41 mg, 0.12 mmol). After 1 h at 60 °C, the reaction mixture was concentrated and purified by reverse phase HPLC (gradient: 30–95% MeCN in water (w/0.05% NH_4_OH)) provide the title compound **14c** (7 mg, 26% yield). ESI-MS [M + H]^+^: 434.3; LCMS Retention time (method (ii)): 1.18 min; ^1^H NMR (600 MHz, DMSO-*d*_6_) δ 10.22 (s, 1H), 8.41 (s, 1H), 7.91 (s, 1H), 7.57–7.61 (m, 2H), 7.44–7.48 (m, 3H), 7.07 (d, *J* = 2.6 Hz, 1H), 6.16 (d, *J* = 2.6 Hz, 1H), 4.07–4.08 (m, 2H), 3.81–3.86 (m, 2H), 3.19–3.24 (m, 2H), 2.25 (s, 3H), 1.90–1.97 (m, 1H), 1.34–1.36 (m, 2H), 1.16–1.25 (m, 2H).

#### (*R*)-Tetrahydrofuran-3-yl 4-Methylbenzenesulfonate (**B8**)

Compound **B8** was prepared using a similar procedure described for **B18**. Compounds **B8** was obtained as a clear, colorless oil (198 mg, 72% yield). ES-MS [M + Na]^+^: 265.4; ^1^H NMR (400 MHz, ((CD_3_)_2_SO)) δ 7.81 (d, *J* = 8.2 Hz, 2H), 7.49 (d, *J* = 8.2 Hz, 2H), 5.12–5.09 (m, 1H), 3.79–3.63 (m, 4H), 2.43 (s, 3H), 2.13–2.02 (m, 1H), 1.93–1.84 (m, 1H).

#### (*S*)-4-Chloro-1-(tetrahydrofuran-3-yl)-1*H*-pyrazole-5-carboxylic Acid (**B9**)

Compound **B9** was prepared using a similar procedure described for **B1**. Compound **B9** was obtained as an off-white solid that was carried forward as a TFA salt (188 mg, 12% yield over 2 steps). ES-MS [M + H]^+^: 217.3; ^1^H NMR (400 MHz, ((CD_3_)_2_SO)) δ 7.74 (s, 1H), 5.76–5.68 (m, 1H), 3.99 (dd, J = 9.5, 6.3 Hz, 1H), 3.95 (dd, J = 15.3, 7.6 Hz, 1H), 3.85–3.77 (m, 2H), 2.42–2.25 (m, 2H).

#### (*S*)-4-Chloro-*N*-(3-methyl-5-(phenylethynyl)­pyridin-2-yl)-1-(tetrahydrofuran-3-yl)-1*H*-pyrazole-5-carboxamide (**15a**)

Compound **15a** was prepared using a similar procedure described for **14c**. Compound **15a** was obtained as an off-white solid (17 mg, 62% yield). ES-MS [M + H]^+^: 407.4; LCMS Retention time: 1.09 min; ^1^H NMR (400 MHz, CDCl_3_) δ 8.50 (s, 1H), 7.87 (s, 1H), 7.58 (s, 1H), 7.57–7.53 (m, 2H), 7.61–7.55 (m, 2H), 7.41–7.36 (m, 3H), 5.93–5.85 (m, 1H), 4.23–4.05 (m, 3H), 3.95 (td, *J* = 8.2, 5.1 Hz, 1H), 2.59–2.50 (m, 1H), 2.46–2.40 (m, 1H), 1.38 (s, 3H).

#### (*S*)-Tetrahydrofuran-3-yl 4-Methylbenzenesulfonate (**B10**)

Compound **B10** was prepared using a similar procedure described for **B18**. Compounds **B10** was obtained as an oil (1400 mg, 51% yield). ES-MS [M + Na]^+^: 265.2.

#### (*R*)-4-Chloro-1-(tetrahydrofuran-3-yl)-1*H*-pyrazole-5-carboxylic Acid (**B11**)

Compound **B11** was prepared using a similar procedure described for **B1**. Compound **B11** was obtained as a white powder (168 mg, 38% yield over 2 steps). ES-MS [M + H]^+^: 217.3.

#### (*R*)-4-Chloro-*N*-(3-methyl-5-(phenylethynyl)­pyridin-2-yl)-1-(tetrahydrofuran-3-yl)-1*H*-pyrazole-5-carboxamide (**15b**)

To a mixture of compound **18a** 11.5 mg, 0.06 mmol, pyridine (0.04 mL, 0.46 mmol), (*R*)-4-Chloro-1-(tetrahydrofuran-3-yl)-1*H*-pyrazole-5-carboxylic acid **B11** (10 mg, 0.05 mmol) in DCE (0.5 mL) was added PyClU (23 mg, 0.07 mmol). The reaction mixture was subjected to microwave irradiation at 60 °C for 30 min. The reaction mixture was concentrated and purified by reverse phase HPLC (gradient: 30–95% MeCN in water (w/0.1% TFA)). The combined desired fractions were added to EtOAC (15 mL) and washed with NaHCO_3_ (aq) (10 mL), brine, dried over MgSO_4_, filtered, and concentrated to provide the title compound **15b** (6 mg, 34% yield). ESI-MS [M + H]^+^: 407.2; LCMS Retention time: 1.19 min (method (ii)); ^1^H NMR (600 MHz, DMSO-*d*_6_) δ 10.97 (s, 1H), 8.41 (s, 1H), 7.96 (s, 1H), 7.69 (s, 1H), 7.57–7.61 (m, 2H), 7.45–7.48 (m, 3H), 5.27–5.31 (m, 1H), 3.96–4.04 (m, 2H), 3.89–3.92 (m, 1H), 3.82–3.86 (m, 1H), 2.35–2.40 (m, 2H), 2.30 (s, 3H).

#### 4-Chloro-1-(1-methyl-1*H*-indazol-5-yl)-1*H*-pyrazole-5-carboxylic Acid (**B12**)

(1-methyl-1*H*-indazol-5-yl)­boronic acid (109.6 mg, 0.62 mmol), methyl 4-chloro-1*H*-pyrazole-5-carboxylate **20** (50 mg, 0.31 mmol), copper­(II) acetate (85 mg, 0.47 mmol) and pyridine (0.05 mL, 0.62 mmol) were stirred with 150 mg 4 Å molecular. After 16 h at rt, the reaction was filtered through Celite and washed with MeOH. The filtrate was concentrated and purified on normal phase column chromatography (gradient: 0–40% EtOAc in hexanes) to afford the title compound (51 mg, 56% yield) as a light yellow solid (ESI-MS [M + H]^+^: 291.4; LCMS Retention time: 0.780 min). The material (51 mg, 0.18 mmol) was dissolved in DMF (1.4 mL) and water (1.42 mL) and NaOH (14.4 mg, 0.36 mmol) was added. The reaction mixture was heated to 60 °C for 30 min. After the heat source was removed, the pH was adjusted to 4–5 and the reaction mixture was extracted with DCM (2 × 10 mL). The combined organic layers were filtered through a hydrophobic filter and the filtrate was concentrated to afford the title compound **B12** (49 mg, 0.17 mmol); ESI-MS [M + H]^+^: 277.2; LCMS Retention time: 0.693 and 0.706 min.

#### 4-Chloro-1-(1-methyl-1*H*-indazol-5-yl)-*N*-(3-methyl-5-(phenylethynyl)­pyridin-2-yl)-1*H*-pyrazole-5-carboxamide (**15c**)

Compound **15c** was prepared using a similar procedure described for **15b**. Compound **15c** was obtained as an off-white solid (2 mg, 6% yield). ESI-MS [M + H]^+^: 467.2; LCMS Retention time: 1.06 min (method (ii)); ^1^H NMR (400 MHz, DMSO-*d*_6_) δ 10.56 (s, 1H), 8.96 (s, 1H), 8.52 (dd, *J* = 2.2, 0.6 Hz, 1H), 8.35 (dd, *J* = 2.1, 0.6 Hz, 1H), 8.19 (d, *J* = 0.9 Hz, 1H), 8.09 (dd, *J* = 9.1, 2.1 Hz, 1H), 7.97 (dd, *J* = 2.2, 0.7 Hz, 1H), 7.86 (d, *J* = 9.2 Hz, 1H), 7.57–7.64 (m, 2H), 7.43–7.50 (m, 3H), 4.11 (s, 3H), 2.29 (s, 3H).

#### 4-Chloro-1-(3-cyanopropyl)-*N*-(3-methyl-5-(phenylethynyl)­pyridin-2-yl)-1*H*-pyrazole-5-carboxamide (**15d**)

A mixture of PyClU (31 mg, 0.09 mmol), DIEA (0.04 mL, 0.23 mmol), intermediate **18a** (14.6 mg, 0.07 mmol), 4-chloro-1-(3-cyanopropyl)-1*H*-pyrazole-5-carboxylic acid **B13** (10 mg, 0.05 mmol) in DCE (1 mL) was subjected to microwave irradiation at 80 °C for 30 min. The reaction mixture was concentrated and purified by reverse phase HPLC (gradient: 40–95% MeCN in water (w/0.1% TFA)). The combined desired fractions were added to EtOAC (15 mL) and washed with NaHCO_3_ (aq) (10 mL), brine, dried over MgSO_4_, filtered and concentrated to provide the title compound (7 mg, 37% yield). ESI-MS [M + Na]^+^: 426.2; LCMS Retention time: 1.18 min (method (ii)); ^1^H NMR (600 MHz, DMSO-*d*_6_) δ 10.91 (s, 1H), 8.39 (s, 1H), 7.95 (s, 1H), 7.69 (s, 1H), 7.57–7.61 (m, 2H), 7.44–7.49 (m, 3H), 4.37 (t, *J* = 6.6 Hz, 2H), 2.52–2.56 (m, 2H), 2.30 (s, 3H), 2.14 (quintet, *J* = 6.9 Hz, 2H).

#### *tert*-Butyl 3-((4-Chloro-5-((3-methyl-5-(phenylethynyl)­pyridin-2-yl)­carbamoyl)-1*H*-pyrazol-1-yl)­methyl)-3-fluoroazetidine-1-carboxylate (**B15**)

A mixture of *tert*-butyl 3-(bromomethyl)-3-fluoroazetidine-1-carboxylate (1402 mg, 5.6 mmol), methyl 4-chloropyrazole-3-carboxylate **20** (750 mg, 4.7 mmol), and K_2_CO_3_ (786 mg, 5.6 mmol) in DMF (10 mL) was stirred at 100 °C for 6 h. The reaction mixture was diluted with ethyl acetate, washed with water and brine, then extracted with DCM (2×). The organic layers were dried with sodium sulfate, filtered, then concentrated *in vacuo*. Crude product was purified by normal phase column chromatography (gradient: 0–20% EtOAc in hexanes) to elute the pure ester intermediate as a colorless amorphous solid (912 mg). The intermediate ester was dissolved in THF (2 mL) and 2 M NaOH (2 mL), then stirred for 2 h at 50 °C. Upon completion of the reaction, the solvent was partially removed *in vacuo* and the mixture was diluted with water. The pH of the solution was adjusted to pH = 3 via slow addition of 2 M HCl, then product was extracted with DCM. The organics were concentrated *in vacuo* to afford **B14** (872 mg, 59% yield over 2 steps). ES-MS [M-^*t*^Bu]+: 278.2; ^1^H NMR (400 MHz, *d*_6_-DMSO-*d*_6_) δ 7.81 (s, 1H), 5.07 (d, *J* = 20.5 Hz, 2H), 4.20 (dd, *J* = 19.7, 10.7 Hz, 2H), 3.89 (dd, *J* = 21.5, 10.4 Hz, 2H), 1.39 (s, 9H).

Compound **B15** is prepared in a similar manner as **Compound 15b** to yield the title compound (**B15**) (128 mg, 41% yield). ES-MS [M + 1]^+^: 524.4.

#### 1-((1-Acetyl-3-fluoroazetidin-3-yl)­methyl)-4-chloro-*N*-(3-methyl-5-(phenylethynyl)­pyridin-2-yl)-1*H*-pyrazole-5-carboxamide (**15e**)

Compound **15e** was prepared in a similar manner as Compound **10** to yield the title compound (**15e**) (68 mg, 60% yield). ES-MS [M + H]^+^: 466.3; LCMS Retention time: 1.14 min; ^1^H NMR (400 MHz, CDCl_3_) δ 8.56 (s, 1H), 8.49 (s, 1H), 7.76 (d, *J* = 1.4 Hz, 1H), 7.61 (s, 1H), 7.56–7.54 (m, 2H), 7.38–7.37 (m, 3H), 5.25 (dd, *J* = 14.9, 14.9 Hz, 1H), 5.06 (dd, *J* = 21.3, 14.6 Hz, 1H), 4.48 (dd, *J* = 17.7, 10.1 Hz, 1H), 4.27–4.17 (m, 2H), 4.12 (dd, *J* = 21.4, 11.5 Hz, 1H), 2.35 (s, 3H), 1.90 (s, 3H).

#### 1-(1-(*tert*-Butoxycarbonyl)­piperidin-4-yl)-4-chloro-1*H*-pyrazole-5-carboxylic Acid (**B16**)

To a mixture of *tert*-butyl 4-bromopiperidine-1-carboxylate (2000 mg, 7.57 mmol), methyl 4-chloropyrazole-3-carboxylate **20** (527 mg, 3.3 mmol), and K_2_CO_3_ (885 mg, 6.31 mmol) was added DMF (5 mL) and the mixture was heated to 100 °C. After 6 h, the mixture was filtered, washed with MeOH and evaporated to remove MeOH. To the mixture was added NaOH (667 mg, 16.3 mmol) and water (2.5 mL). After an additional 1 h at 100 °C, the reaction was removed from the heat source. At rt, the mixture was diluted with water and then 2 M HCl was added dropwise until pH = 4–5. After the mixture was extracted with CHCl_3_:IPA (3:1, 3×), the collected organic layers were concentrated *in vacuo* and then purified by reverse phase HPLC (gradient: 25–65% MeCN in water (w/0.1% TFA)). The desired fractions were concentrated to yield **B16** as an off-white solid that was carried forward as a TFA salt (904 mg, 36% yield over 2 steps). ES-MS [M + Na]^+^: 352.3; ^1^H NMR (400 MHz, CDCl_3_) δ 7.54 (s, 1H), 5.26–5.15 (m, 1H), 4.26 (br d, *J* = 12.9 Hz, 2H), 2.97–2.83 (m, 2H), 2.08 (dddd, *J* = 13.1, 12.2, 11.9, 4.2 Hz, 2H), 2.01–1.93 (m, 2H), 1.47 (s, 9H).

#### *tert*-Butyl 4-Chloro-5-((3-methyl-5-(phenylethynyl)­pyridin-2-yl)­carbamoyl)-(1*H*-pyrazol-1-yl)­piperidine-1-carboxylate (**B17**)

To a mixture of **B16** (3500 mg, 10.6 mmol), Intermediate **18a** (3315 mg, 15.9 mmol), PyClU (7061 mg, 21.2 mmol), and pyridine (8.6 mL, 106.1 mmol) at rt was added DCE (21.3 mL). After 1.5 h, the reaction mixture was concentrated *in vacuo* and then purified by reverse phase HPLC (gradient: 45–95% MeCN in water (w/0.1% TFA)) to yield **B17** as a tan solid that was carried forward as a TFA salt (2230 mg, 33% yield). ES-MS [M + H]^+^: 520.0; LCMS Retention time: 1.21 min; ^1^H NMR (400 MHz, CDCl_3_) δ 10.98 (s, 1H), 8.41 (d, *J* = 1.6 Hz, 1H), 7.95 (d, *J* = 1.6 Hz, 1H), 7.68 (s, 1H), 7.60–7.55 (m, 2H), 7.48–7.43 (m, 3H), 4.70–4.59 (m, 1H), 4.04 (d, *J* = 12.0 Hz, 2H), 3.0–2.76 (m, 2H), 2.29 (s, 3H), 2.03–1.80 (m, 4H), 1.41 (s, 9H).

#### 1-(1-Acetylpiperidin-4-yl)-4-chloro-*N*-(3-methyl-5-(phenylethynyl)­pyridin-2-yl)-1*H*-pyrazole-5-carboxamide (**10**; **ONO-9517601**)

To the TFA salt of **B17** (4585 mg, 7.23 mmol) was added DCM:TFA (1:1; 10 mL) at rt. After 1 h, the mixture was concentrated *in vacuo* to give a tan solid. ES-MS [M + 1]^+^: 420.4. The resulting crude amine was dissolved in DMF (36 mL) and DIEA (18.9 mL, 108 mmol) was added. To the mixture was added AcOH (2.1 mL, 36.2 mmol) and HATU (6050 mg, 15.9 mmol). After 30 min, the mixture was concentrated *in vacuo* and then purified by reverse phase HPLC (gradient: 50–90% MeCN in water (w/0.05% NH_4_OH)). The desired fractions were concentrated to give **10** (**ONO-9517601**) as an off-white solid (2787 mg, 91% over 2 steps). ES-MS [M + H]^+^: 462.0; LCMS Retention time: 1.07 min; ^1^H NMR (600 MHz, DMSO-*d*_6_) δ 10.98 (s, 1H), 8.40 (s, 1H), 7.94 (br s, 1H), 7.67 (s, 1H), 7.56–7.59 (m, 2H), 7.43–7.46 (m, 3H), 4.67–4.73 (m, 1H), 4.45 (br d, *J* = 13.4 Hz, 1H), 3.91 (br d, *J* = 13.7 Hz, 1H), 3.16–3.20 (m, 1H), 2.66–2.71 (m, 1H), 2.29 (s, 3H), 1.95–2.02 (m, 6H), 1.77–1.84 (m, 1H); ^13^C NMR (150 MHz, DMSO-*d*_6_) δ 168.11, 158.03, 148.77, 148.03, 141.59, 136.63, 134.04, 131.42, 129.18, 128.84, 128.01, 121.74, 117.34, 108.39, 92.14, 85.87, 57.38, 44.51 and 40.0–39.0 (overlapped with the solvent peak), 32.11 and 31.40, 21.21, 17.29; HRMS: Obs: 462.1699, Calcd: 462.1691 for C_25_H_25_ClN_5_O_2_ [M + H]^+^; mp 172–173 °C.

#### *trans*-4-((*tert*-Butoxycarbonyl)­amino)­cyclohexyl 4-methylbenzenesulfonate (**B18**)

To a solution of *trans*-*tert*-butyl (4-hydroxycyclohexyl)­carbamate (5000 mg, 23.22 mmol) in DCM (40.3 mL) was added 4-methylbenzenesulfonyl chloride (5313 mg, 27.87 mmol) and TEA (6.7 mL, 47.72 mmol) at 0 °C. The ice bath was removed, and after 17 h at rt, the sample was concentrated *in vacuo* and purified by normal phase chromatography (gradient: 0–40% EtOAc in hexanes) to give **B18** as an off-white solid (3523 mg, 41% yield). ES-MS [M + Na]^+^: 392.4; ^1^H NMR (400 MHz, (CD_3_)_2_SO) δ 7.79 (d, *J* = 8.2 Hz, 2H), 7.47 (d, *J* = 8.2 Hz, 2H), 6.72 (d, *J* = 7.4 Hz, 1H), 4.39–4.29 (m, 1H), 3.3.26–3.14 m, 1H, 2.42 (s, 3H), 1.81–1.66 (m, 4H), 1.53–1.39 (m, 2H), 1.35 (s, 9H), 1.25–1.10 (m, 2H).

#### 1-(*cis*-4-((*tert*-Butoxycarbonyl)­amino)­cyclohexyl)-4-chloro-1*H*-pyrazole-3-carboxylic Acid (**B19a**) and 1-(*cis*-4-((*tert*-Butoxycarbonyl)­amino)­cyclohexyl)-4-chloro-1*H*-pyrazole-5-carboxylic Acid (**B19b**)

Compounds **B19a** and **B19b** were prepared using a similar procedure described for **B1**. Compounds **B19a** and **B19b** were separated by reverse phase HPLC (gradient: 30–65% MeCN in water (w/0.1% TFA)). Compound **B19a** was obtained as an off-white solid that was carried forward as a TFA salt (26 mg, 8% yield over 2 steps). ES-MS [M + Na]^+^: 366.4, ^1^H NMR (400 MHz, ((CD_3_)_2_SO)) δ 8.19 (s, 1H), 6.90 (d, *J* = 6.9 Hz, 1H), 4.26–4.16 (m, 1H), 3.60 (br s, 1H), 2.15–2.02 (m, 2H), 1.83–1.73 (m, 2H),1.71–1.54 (m, 4H), 1.39 (s, 9H). Compound **B19b** was obtained as a white solid that was carried forward as a TFA salt (316 mg, 44% yield over 2 steps). ES-MS [M-^*t*^Bu + 2H]^+^: 288.4; ^1^H NMR (400 MHz, ((CD_3_)_2_SO)) δ 7.71 (s, 1H), 6.90 (br s, 1H), 5.01–4.90 (m, 1H), 3.59–3.51 (m, 1H), 2.15–2.00 (m, 2H), 1.89–1.79 (m, 2H),1.72–1.63 (m, 2H), 1.60–1.49 (m, 2H), 1.39 (s, 9H).

#### *tert*-Butyl (*cis*-4-(4-Chloro-5-((3-methyl-5-(phenylethynyl)­pyridin-2-yl)­carbamoyl)-1*H*-pyrazol-1-yl)­cyclohexyl)­carbamate (**B20**)

Compound **B20** was prepared using a similar procedure described for **B17**. Compound **B20** was obtained as a brown solid (654 mg, 50% yield). ES-MS [M + H]^+^: 534.2; LCMS Retention time: 1.27 min; ^1^H NMR (400 MHz, (CD_3_)_2_SO) δ 10.96 (s, 1H), 8.41 (d, J = 1.7 Hz, 1H), 7.95 (dd, *J* = 2.0, 0.7 Hz, 1H), 7.65 (s, 1H), 7.65–7.58 (m, 2H), 7.48–7.43 (m, 3H), 6.91 (br s, 1H), 4.54–4.42 (m, 1H), 3.57–3.49 (m, 1H), 2.28 (s, 3H), 2.22–2.09 (m, 2H), 1.90–1.80 (m, 2H), 1.80–1.70 (m, 2H),1.62–1.50 (m, 2H), 1.40 (s, 9H).

#### 1-(*cis*-4-Acetamidocyclohexyl)-4-chloro-*N*-(3-methyl-5-(phenylethynyl)­pyridin-2-yl)-1*H*-pyrazole-5-carboxamide (**15f**)

To intermediate **B20** (595 mg, 0.918 mmol) was added DCM:TFA (1:1; 4 mL) at rt. After 1 h, the mixture was concentrated *in vacuo* to give a tan solid. ES-MS [M + 1]^+^: 434.3. To the resulting crude amine in DMF (3.6 mL) was added DIEA (2.18 mL, 12.5 mmol). To the mixture was added AcOH (0.22 mL, 3.78 mmol) and HATU (632 mg, 1.66 mmol). After 30 min, NH_4_OH (1 mL) was added to the mixture, and the mixture was stirred for 1 h. The mixture was concentrated *in vacuo* and then purified by reverse phase HPLC (gradient: 40–85% MeCN in water (w/0.05% NH_4_OH)). The desired fractions were concentrated to give Compound **15f** as an off-white solid (349 mg, 98% yield over 2 steps). ES-MS [M + H]^+^: 476.3; LCMS Retention time: 1.08 min; ^1^H NMR (400 MHz, (CD_3_)_2_SO) δ 10.96 (s, 1H), 8.40 (d, *J* = 1.5 Hz, 1H), 7.94 (d, *J* = 1.5 Hz, 1H), 7.92 (d, *J* = 6.8 Hz, 1H), 7.66 (s, 1H), 7.62–7.55 (m, 2H), 7.48–7.42 (m, 3H), 4.54–4.44 (m, 1H), 3.86–3.78 (m, 1H), 2.28 (s, 3H), 2.21–2.08 (m, 2H), 1.85 (s, 3H), 1.83–1.76 (m, 4H), 1.65–1.53 (m, 2H).

#### *trans*-4-((*tert*-Butoxycarbonyl)­amino)­cyclohexyl 4-methylbenzenesulfonate (**B21**)

To a solution of *trans*-*tert*-butyl (4-hydroxycyclohexyl)­carbamate (5000 mg, 23.22 mmol) in DCM (40.3 mL) was added 4-methylbenzenesulfonyl chloride (5313 mg, 27.87 mmol) and TEA (6.7 mL, 47.72 mmol) at 0 °C. The ice bath was removed and after 17 h at rt, the sample was concentrated *in vacuo* and purified by normal phase chromatography (gradient: 0–40% EtOAc in hexanes) to give the title compound as an off-white solid (3523 mg, 41% yield). ES-MS [M + Na]^+^: 404.4; LCMS Retention time: 0.985 min.

#### 1-((3a*R*,5*s*,6a*S*)-2-(*tert*-Butoxycarbonyl)­octahydrocyclopenta­[*c*]­pyrrol-5-yl)-4-chloro-1*H*-pyrazole-5-carboxylic Acid (**B22**)

A mixture of **B21** (2545 mg, 6.67 mmol), methyl 4-chloro-1*H*-pyrazole-3-carboxylate **20** (464 mg, 2.89 mmol), and K_2_CO_3_ (780 mg, 5.56 mmol) in DMF (8.8 mL) was heated to 100 °C for 17 h on benchtop. The reaction was then filtered and NaOH (547 mg, 13.3 mmol) and H_2_O (4.0 mL) were added to the mixture at rt. After 17 h, the mixture was diluted with water and then 2 M HCl was added dropwise until pH = 4–5. After the mixture was extracted with CHCl_3_:IPA (3:1, 3×), the collected organic layers were concentrated *in vacuo* and then purified by reverse phase HPLC (gradient: 35–75% MeCN in water (w/0.1% TFA)). The desired fractions were concentrated to yield compound B22 as a white solid that was carried forward as a TFA salt (324 mg, 15% yield over 2 steps). ES-MS [M + Na]^+^: 378.0; LCMS Retention time: 0.969 min.

#### 1-((3a*R*,5*s*,6a*S*)-2-Acetyloctahydrocyclopenta­[*c*]­pyrrol-5-yl)-4-chloro-*N*-(3-methyl-5-(phenylethynyl)­pyridin-2-yl)-1*H*-pyrazole-5-carboxamide (**15g**)

Compound **15g** was prepared using a similar procedure described for compound **10**. Compound **15g** was obtained as an off-white solid (6 mg, 83% yield over 2 steps). ESI-MS [M + H]^+^: 488.3; LCMS Retention time: 1.14 min (method (ii)); ^1^H NMR (600 MHz, DMSO-*d*_6_) δ 10.96 (s, 1H), 8.42 (s, 1H), 7.95 (s, 1H), 7.67–7.69 (m, 1H), 7.57–7.61 (m, 2H), 7.44–7.48 (m, 3H), 5.00–5.21 (m, 1H), 3.62–3.66 (m, 1H), 3.47–3.52 (m, 1H), 3.25–3.45 (m, 1H), 3.14–3.19 (m, 1H), 2.85–3.00 (m, 2H), 2.25–2.32 (m, 5H), 1.96–2.07 (m, 2H), 1.92 (s, 3H).

#### (*S*)-5-((1,4-Dioxan-2-yl)­methoxy)-2-chloro-*N*-(3-methyl-5-(phenylethynyl)­pyridin-2-yl)­benzamide (**7**)

##### Step A: (**B8**) (*S*)-5-((1,4-Dioxan-2-yl)­methoxy)-2-chlorobenzoic Acid

A solution of ethyl 2-chloro-5-hydroxybenzoate (2579 mg, 12.9 mmol), (*S*)-(1,4-dioxan-2-yl)­methyl 4-methylbenzenesulfonate (3500 mg, 12.9 mmol), and K_2_CO_3_ (7209 mg, 51.4 mmol) in DMF (103 mL) was subjected to microwave irradiation at 115 °C for 45 min. The solution was diluted with water and extracted with EtOAc (1 × 300 mL). The extraction was washed with water (2 × 50 mL) and concentrated *in vacuo*. The residue was dissolved in THF (200 mL), MeOH (80 mL), and NaOH (8458 mg, 206 mmol) was added. After 2 h at rt, the solution was diluted with water and EtOAc and treated with 2N HCL solution to lower pH to <5. The reaction mixture was extracted with EtOAc (1 × 1 L). The organic layer was washed with water (3 × 1 L). Each water wash was re-extracted with EtOAc (3 × 250 mL). The combined organic layers were concentrated *in vacuo*. The residue was dissolved in DCM and filtered through a hydrophobic frit to yield the title compound (3500 mg, 99% yield), which was taken forward without further purification.

##### Step B: (**7**) (*S*)-5-((1,4-Dioxan-2-yl)­methoxy)-2-chloro-*N*-(3-methyl-5-(phenylethynyl)­pyridin-2-yl)­benzamide

Equally divided into 7 vials, was added PyClU (6404 mg, 19.3 mmol), pyridine (5.19 mL, 64.2 mmol), 2-chloro-5-[[(*2S*)-1,4-dioxan-2-yl]­methoxy]­benzoic acid (**B2**) (3500 mg, 12.8 mmol), compound **18a** (4009 mg, 19.3 mmol), and DCE (67 mL). After 1 h at 50 °C, the solution was diluted with water (500 mL), and extracted with DCM (3 × 300 mL). The DCM extracts were washed with water (2 × 300 mL), combined, and concentrated *in vacuo*. The residue was purified by reverse phase HPLC (gradient: 30–80% MeCN in water (w/0.1% TFA)). The fractions were partially concentrated *in vacuo* and diluted with NaHCO_3_ aqueous solution (0.5 mL), water, and EtOAc. The sample was extracted with EtOAc (2 × 500 mL). The combined organic layers were washed with water (3 × 300 mL) and concentrated. The resulting solids were sonicated in MeOH, and the resulting slurry was filtered to afford compound **7** (1196 mg, 20% yield) ES-MS [M + H]^+^: 463.2; LCMS Retention time: 1.05 min; ^1^H NMR (400 MHz, CDCl_3_) δ 9.13 (br s, 1H), 8.40 (s, 1H), 7.91 (s, 1H), 7.57–7.54 (m, 2H), 7.42–7.34 (m, 5H), 7.01 (dd, J = 8.9, 3.0 Hz, 1H), 4.06–4.0 (m, 3H), 3.91–3.84 (m, 2H), 3.81 (ddd, J = 10.4, 2.7, 2.7 Hz, 1H), 3.75 (d, J = 11.3 Hz, 1H), 3.67 (ddd, J = 11.5, 10.5, 3.3 Hz, 1H), 3.55 (dd, J = 11.3, 9.3 Hz, 1H), 2.44 (s, 3H).

#### 1-(1-Acetylpiperidin-4-yl)-4-chloro-*N*-(3-methyl-5-(phenylethynyl)­pyridin-2-yl)-1*H*-pyrazole-5-carboxamide (**10**; **ONO-9517601**)

##### Step A: (**B16**) 1-(1-(*tert*-Butoxycarbonyl)­piperidin-4-yl)-4-chloro-1*H*-pyrazole-5-carboxylic Acid

To a mixture of *tert*-butyl 4-bromopiperidine-1-carboxylate (2000 mg, 7.57 mmol), methyl 4-chloropyrazole-3-carboxylate **20** (527 mg, 3.3 mmol), and K_2_CO_3_ (885 mg, 6.31 mmol) was added DMF (5 mL) and the mixture was heated to 100 °C. After 6 h, the mixture was filtered, washed with MeOH and evaporated to remove MeOH. To the mixture was added NaOH (667 mg, 16.3 mmol) and water (2.5 mL). After an additional 1 h at 100 °C, the reaction was removed from the heat source. At rt, the mixture was diluted with water and then 2 M HCl was added dropwise until pH = 4–5. After the mixture was extracted with CHCl_3_:IPA (3:1, 3×), the collected organic layers were concentrated *in vacuo* and then purified by reverse phase HPLC (gradient: 25–65% MeCN in water (w/0.1% TFA)). The desired fractions were concentrated to yield **B16** as an off-white solid that was carried forward as a TFA salt (904 mg, 36% yield over 2 steps). ES-MS [M + Na]^+^: 352.3; ^1^H NMR (400 MHz, CDCl_3_) δ 7.54 (s, 1H), 5.26–5.15 (m, 1H), 4.26 (br d, *J* = 12.9 Hz, 2H), 2.97–2.83 (m, 2H), 2.08 (dddd, *J* = 13.1, 12.2, 11.9, 4.2 Hz, 2H), 2.01–1.93 (m, 2H), 1.47 (s, 9H).

##### Step B: (**B17**) *tert*-Butyl 4-chloro-5-(((3-methyl-5-(phenylethynyl)­pyridin-2-yl)­carbamoyl)-1*H*-pyrazol-1-yl)­piperidine-1-carboxylate

To a mixture of **B16** (3500 mg, 10.6 mmol), Intermediate **18a** (3315 mg, 15.9 mmol), PyClU (7061 mg, 21.2 mmol), and pyridine (8.6 mL, 106.1 mmol) at rt was added DCE (21.3 mL). After 1.5 h, the reaction mixture was concentrated *in vacuo* and then purified by reverse phase HPLC (gradient: 45–95% MeCN in water (w/0.1% TFA)) to yield **B17** as a tan solid that was carried forward as a TFA salt (2230 mg, 33% yield). ES-MS [M + H]^+^: 520.0; LCMS Retention time: 1.21 min; ^1^H NMR (400 MHz, CDCl_3_) δ 10.98 (s, 1H), 8.41 (d, *J* = 1.6 Hz, 1H), 7.95 (d, *J* = 1.6 Hz, 1H), 7.68 (s, 1H), 7.60–7.55 (m, 2H), 7.48–7.43 (m, 3H), 4.70–4.59 (m, 1H), 4.04 (d, *J* = 12.0 Hz, 2H), 3.0–2.76 (m, 2H), 2.29 (s, 3H), 2.03–1.80 (m, 4H), 1.41 (s, 9H).

##### Step C: (**10**, **ONO-9517601**) 1-(1-Acetylpiperidin-4-yl)-4-chloro-*N*-(3-methyl-5-(phenylethynyl)­pyridin-2-yl)-1*H*-pyrazole-5-carboxamide

To the TFA salt of **B17** (4585 mg, 7.23 mmol) was added DCM:TFA (1:1; 10 mL) at rt. After 1 h, the mixture was concentrated *in vacuo* to give a tan solid. ES-MS [M + 1]^+^: 420.4. The resulting crude amine was dissolved in DMF (36 mL) and DIEA (18.9 mL, 108 mmol) was added. To the mixture was added AcOH (2.1 mL, 36.2 mmol) and HATU (6050 mg, 15.9 mmol). After 30 min, the mixture was concentrated *in vacuo* and then purified by reverse phase HPLC (gradient: 50–90% MeCN in water (w/0.05% NH_4_OH)). The desired fractions were concentrated to give **10** (**ONO-9517601**) as an off-white solid (2787 mg, 91% over 2 steps). ES-MS [M + H]^+^: 462.0; LCMS Retention time: 1.07 min; ^1^H NMR (600 MHz, DMSO-*d*_6_) δ 10.98 (s, 1H), 8.40 (s, 1H), 7.94 (br s, 1H), 7.67 (s, 1H), 7.56–7.59 (m, 2H), 7.43–7.46 (m, 3H), 4.67–4.73 (m, 1H), 4.45 (br d, *J* = 13.4 Hz, 1H), 3.91 (br d, *J* = 13.7 Hz, 1H), 3.16–3.20 (m, 1H), 2.66–2.71 (m, 1H), 2.29 (s, 3H), 1.95–2.02 (m, 6H), 1.77–1.84 (m, 1H); ^13^C NMR (150 MHz, DMSO-*d*_6_) δ 168.11, 158.03, 148.77, 148.03, 141.59, 136.63, 134.04, 131.42, 129.18, 128.84, 128.01, 121.74, 117.34, 108.39, 92.14, 85.87, 57.38, 44.51 and 40.0–39.0 (overlapped with the solvent peak), 32.11 and 31.40, 21.21, 17.29; HRMS: Obs: 462.1699, Calcd: 462.1691 for C_25_H_25_ClN_5_O_2_ [M + H]^+^; mp 172–173 °C.

#### 1-(1-(*tert*-Butoxycarbonyl)-3-fluoropiperidin-4-yl)-4-chloro-1*H*-pyrazole-5-carboxylic Acid (**B24**)

Compound **B24** was prepared using a similar procedure described for **B16**. Compound **B24** was obtained as an off-white solid that was carried forward as a TFA salt (12 mg, 6% yield). ES-MS [M + Na]^+^: 370.3; LCMS Retention time: 0.916 min; 1H NMR (400 MHz, DMSO-*d*_6_) δ 7.85 (s, 1H), 5.51–5.38 (m, 1H), 4.91–4.68 (m, 1H), 4.27 (br s, 1H), 3.96 (br s, 1H), 3.03 (d, *J* = 33.5 Hz, 3H), 2.11–2.02 (m, 1H), 1.96–1.82 (m, 1H), 1.42 (s, 9H).

#### 1-(1-Acetyl-3-fluoropiperidin-4-yl)-4-chloro-*N*-(3-methyl-5-(phenylethynyl)­pyridin-2-yl)-1*H*-pyrazole-5-carboxamide (**16a**)

Compound **16a** was prepared using a similar procedure described for compound **10**. Compound **16a** was obtained as an off-white solid (6 mg, 96% yield over 2 steps). ESI-MS [M + Na]^+^: 502.2; LCMS Retention time: 1.15 min (method (ii)); ^1^H NMR (600 MHz, DMSO-*d*_6_) δ 11.03–11.04 (m, 1H), 8.46 (s, 1H), 7.97–7.98 (m, 1H), 7.82 (s, 1H), 7.59–7.61 (m, 2H), 7.46–7.48 (m, 3H), 3.86–5.06 (m, 4H), 2.39–3.50 (m, 2H), 2.29 (s, 3H), 1.85–2.19 (m, 5H).

#### *tert-*Butyl (3*R*,4*S*)-3-Fluoro-4-(tosyloxy)­piperidine-1-carboxylate (**B26**)

To a solution of *tert*-butyl (3*R*,4*S*)-3-fluoro-4-hydroxypiperidine-1-carboxylate (1000 mg, 4.56 mmol) in DCM (4.0 mL) was added 4-methylbenzenesulfonyl chloride (1043 mg, 5.47 mmol) and TEA (1.27 mL, 9.12 mmol) at 0 °C. The reaction was warmed to rt and stirred for 17 h. The sample was concentrated *in vacuo* and purified by normal phase chromatography (gradient: 0–40% EtOAc in hexanes) to provide **B26** as an off-white solid (1577 mg, 93%). ES-MS [M + Na]^+^: 396.3; LCMS Retention time: 1.008 min; ^1^H NMR (400 MHz, DMSO-*d*_6_) δ 7.86–7.79 (m, 2H), 7.49 (d, *J* = 0.7 Hz, 2H), 4.88–4.77 (m, 1H), 4.78–4.61 (m, 1H), 4.03–3.93 (m, 1H), 3.76 (br s, 1H), 3.28–3.08 (m, 1H), 3.07–2.79 (m, 1H), 2.43 (s, 3H), 1.79–1.66 (m, 1H), 1.64–1.55 (m, 1H), 1.36 (s, 9H).

#### 1-((3*R*,4*R*)-1-(*tert*-Butoxycarbonyl)-3-fluoropiperidin-4-yl)-4-chloro-1*H*-pyrazole-5-carboxylic Acid (**B27**)

A mixture of **B26** (1547 mg, 4.14 mmol), methyl 4-chloro-1*H*-pyrazole-3-carboxylate (665 mg, 4.14 mmol), and K_2_CO_3_ (697 mg, 4.97 mmol) in DMF (8.0 mL) was heated to 100 °C. After 17 h, NaOH (510 mg, 12.43 mmol) and H_2_O (4.0 mL) were added to the mixture. After 1 h at 100 °C, the mixture was cooled to rt, diluted with water and then 2 M HCl was added dropwise until pH = 4–5. After the mixture was extracted with CHCl_3_:IPA (3:1, 3×), the collected organic layers were concentrated *in vacuo* and then purified by reverse phase HPLC (gradient: 30–65% MeCN in water (w/0.1% TFA)). The desired fractions were concentrated to yield Compound **B27** as an off-white solid that was carried forward as a TFA salt (384 mg, 20% yield over 2 steps). ES-MS [M-^*t*^Bu+2H]^+^: 292.1; LCMS Retention time: 0.865 min; ^1^H NMR (400 MHz, DMSO-*d*_6_) δ 7.85 (s, 1H), 5.51–5.38 (m, 1H), 4.90–4.68 (m, 1H), 4.26 (br s, 1H), 3.96 (d, *J* = 12.2 Hz, 1H), 2.98 (br s, 3H), 2.11–2.02 (m, 1H), 1.96–1.82 (m, 1H), 1.42 (s, 9H).

#### 1-((3*R*,4*R*)-1-Acetyl-3-fluoropiperidin-4-yl)-4-chloro-*N*-(3-methyl-5-(phenylethynyl)­pyridin-2-yl)-1*H*-pyrazole-5-carboxamide (**16b**)

Compound **16b** was prepared using a similar procedure described for compound 10. Compound **16b** was obtained as an off-white solid (3 mg, 84% yield over 2 steps). ESI-MS [M + Na]^+^: 502.2; LCMS Retention time: 1.15 min (method (ii)); ^1^H NMR (600 MHz, DMSO-*d*_6_) δ 11.04 (s, 1H), 8.46 (s, 1H), 7.97 (s, 1H), 7.82 (s, 1H), 7.59–7.61 (m, 2H), 7.46–7.48 (m, 3H), 4.68–5.06 (m, 2.5H), 3.86–4.41 (m, 1.5H), 2.72–3.50 (m, 2H), 2.28 (s, 3H), 1.84–2.20 (m, 5H).

#### *tert-*Butyl (3*S*,4*S*)-3-Fluoro-4-(tosyloxy)­piperidine-1-carboxylate (**B29**)

Compound **B29** was prepared using a similar procedure described for **B26**. Compound **B29** was obtained as an off-white amorphous solid (1289 mg, 76%). ES-MS [M + Na]^+^: 396.4; LCMS Retention time: 1.034 min.

#### 1-((3*S*,4*R*)-1-(*tert*-Butoxycarbonyl)-3-fluoropiperidin-4-yl)-4-chloro-1*H*-pyrazole-5-carboxylic Acid (**B30**)

Compound **B30** was prepared using a similar procedure described for **B27**. Compound **B30** was obtained as an off-white solid that was carried forward as a TFA salt (37 mg, 4% yield over 2 steps). ES-MS [M-^*t*^Bu+2H]^+^: 292.2; LCMS Retention time: 0.902 min.

#### *tert-*Butyl (3*S*,4*R*)-4-(4-Chloro-5-((3-methyl-5-(phenylethynyl)­pyridin-2-yl)­carbamoyl)-1*H*-pyrazol-1-yl)-3-fluoropiperidine-1-carboxylate (**B31**)

Compound **B31** was prepared using a similar procedure described for **B17**. Compound **B31** was obtained as a yellow solid (8 mg, 18% yield). ES-MS [M + H]^+^: 538.3; LCMS Retention time: 1.268 min.

#### 1-((3*S*,4*R*)-1-Acetyl-3-fluoropiperidin-4-yl)-4-chloro-*N*-(3-methyl-5-(phenylethynyl)­pyridin-2-yl)-1*H*-pyrazole-5-carboxamide (**16c**)

Compound **16c** was prepared using a similar procedure described for compound **10**. Compound **16c** was obtained as an off-white solid (4 mg, 85% yield over 2 steps). ESI-MS [M + Na]^+^: 502.2; LCMS Retention time: 1.12 min (method (ii)); ^1^H NMR (600 MHz, DMSO-*d*_6_) δ 10.98–10.99 (m, 1H), 8.42 (s, 1H), 7.96 (s, 1H), 7.71 (d, *J* = 2.9 Hz, 1H), 7.56–7.62 (m, 2H), 7.44–7.48 (m, 3H), 4.97–5.12 (m, 2H), 4.52–4.72 (m, 1H), 4.00–4.19 (m, 1H), 3.22–3.55 (m, 1H), 2.82–3.02 (m, 1H), 2.39–2.62 (m, 1H), 2.30 (s, 3H), 2.00–2.08 (m, 3H), 1.86–1.93 (m, 1H).

#### *tert-*Butyl (3*R*,4*R*)-3-Fluoro-4-(tosyloxy)­piperidine-1-carboxylate (**B32**)

Compound **B32** was prepared using a similar procedure described for **B26**. Compound **B32** was obtained as a white solid (1155 mg, 68%). ES-MS [M + Na]^+^: 396.4; LCMS Retention time: 0.997; ^1^H NMR (400 MHz, CDCl_3_) δ 7.84–7.76 (m, 2H), 7.38–7.31 (m, 2H), 4.63–4.54 (m, 1H), 4.50–4.31 (m, 1H), 3.79 (br s, 1H), 3.61–3.19 (m, 3H), 2.45 (s, 3H), 2.13–2.01 (m, 1H), 1.72–1.63 (m, 1H), 1.43 (s, 9H).

#### 1-((3*R*,4*S*)-1-(*tert*-Butoxycarbonyl)-3-fluoropiperidin-4-yl)-4-chloro-1*H*-pyrazole-5-carboxylic Acid (**B33**)

Compound **B33** was prepared using a similar procedure described for **B27**. Compound **B33** was obtained as a white amorphous solid that was carried forward as a TFA salt (13 mg, 1% yield over 2 steps). ES-MS [M-^*t*^Bu+2H]^+^: 292.4; LCMS Retention time: 0.898 min.

#### *tert*-Butyl (3*R*,4*S*)-4-(4-Chloro-5-((3-methyl-5-(phenylethynyl)­pyridin-2-yl)­carbamoyl)-1*H*-pyrazol-1-yl)-3-fluoropiperidine-1-carboxylate (**B34**)

Compound **B34** was prepared using a similar procedure described for **B17**. Compound **B34** was obtained as a yellow solid (9 mg, 36% yield). ES-MS [M + H]^+^: 538.3; LCMS Retention time: 1.276 min.

#### 1-((3*R*,4*S*)-1-Acetyl-3-fluoropiperidin-4-yl)-4-chloro-*N*-(3-methyl-5-(phenylethynyl)­pyridin-2-yl)-1*H*-pyrazole-5-carboxamide (**16d**)

Compound **16d** was prepared using a similar procedure described for compound **10**. Compound **16d** was obtained as an off-white solid (5 mg, 80% yield over 2 steps). ESI-MS [M + Na]^+^: 502.2; LCMS Retention time: 1.12 min (method (ii)); ^1^H NMR (600 MHz, DMSO-*d*_6_) δ 10.98–10.99 (m, 1H), 8.42 (s, 1H), 7.96 (s, 1H), 7.71 (d, *J* = 2.9 Hz, 1H), 7.56–7.62 (m, 2H), 7.44–7.48 (m, 3H), 4.95–5.13 (m, 2H), 4.52–4.72 (m, 1H), 3.98–4.19 (m, 1H), 3.22–3.55 (m, 1H), 2.81–3.04 (m, 1H), 2.39–2.62 (m, 1H), 2.30 (s, 3H), 2.00–2.08 (m, 3H), 1.85–1.93 (m, 1H).

#### *tert*-Butyl (3*R*,4*R*)-4-(4-Chloro-5-((3-fluoro-5-(phenylethynyl)­pyridin-2-yl)­carbamoyl)-1*H*-pyrazol-1-yl)-3-fluoropiperidine-1-carboxylate (**B35**)

Compound **B35** was prepared using a similar procedure described for **B17** but the reaction was run for 2 h. Compound **B35** was obtained as an off-white solid (7 mg, 21% yield). ES-MS [M + H]^+^: 541.3; LCMS Retention time: 1.238 min.

#### 1-((3*R*,4*S*)-1-Acetyl-3-fluoropiperidin-4-yl)-4-chloro-*N*-(3-fluoro-5-(phenylethynyl)­pyridin-2-yl)-1*H*-pyrazole-5-carboxamide (**16e**)

Compound **16e** was prepared using a similar procedure described for compound **10**. Compound **16e** was obtained as an off-white solid (2 mg, 80% yield over 2 steps). ESI-MS [M + Na]^+^: 506.2; LCMS Retention time: 1.15 min (method (ii)); ^1^H NMR (600 MHz, DMSO-*d*_6_) δ 11.38 (br s, 1H), 8.50 (s, 1H), 8.13 (d, *J* = 10.5 Hz, 1H), 7.85 (s, 1H), 7.61–7.64 (m, 2H), 7.47–7.50 (m, 3H), 4.68–5.05 (m, 2.5H), 4.39–4.41 (m, 0.5H), 4.19–4.21 (m, 0.5H), 3.88–3.90 (m, 0.5H), 3.15–3.50 (m, 1H), 2.71–2.87 (m, 1H), 1.84–2.17 (m, 5H).

#### *tert*-Butyl (3*S*,4*R*)-3-Fluoro-4-(tosyloxy)­piperidine-1-carboxylate (**B36**)

Compound **B36** was prepared using a similar procedure described for **B26**. Compound **36** was obtained as a white amorphous solid (561 mg, 66%). ES-MS [M + Na]^+^: 396.3; LCMS Retention time: 1.033 min.

#### 1-((3*S*,4*S*)-1-(*tert*-Butoxycarbonyl)-3-fluoropiperidin-4-yl)-4-chloro-1*H*-pyrazole-5-carboxylic Acid (**B37**)

Compound **B37** was prepared using a similar procedure described for **B37**. Compound **B37** was obtained as an off-white solid that was carried forward as a TFA salt (58 mg, 11% yield over 2 steps). ES-MS [M-^*t*^Bu+2H]^+^: 292.4; LCMS Retention time: 0.858 min; ^1^H NMR (400 MHz, DMSO-*d*_6_) δ 7.84 (s, 1H), 5.53–5.33 (m, 1H), 4.97–4.66 (m, 1H), 4.27 (br s, 1H), 3.96 (d, *J* = 11.4 Hz, 1H), 2.98 (br s, 3H), 2.16–2.01 (m, 1H), 1.97–1.79 (m, 1H), 1.42 (s, 9H).

#### *tert*-Butyl (3*S*,4*S*)-4-(4-Chloro-5-((3-fluoro-5-(phenylethynyl)­pyridin-2-yl)­carbamoyl)-1*H*-pyrazol-1-yl)-3-fluoropiperidine-1-carboxylate (**B38**)

Compound **B38** was prepared using a similar procedure described for **B17** but the reaction was run for 2 h. Compound **B38** was obtained as a yellow solid (7 mg, 31% yield). ES-MS [M + H]^+^: 542.2; LCMS Retention time: 1.163 min.

#### 1-((3*S*,4*S*)-1-Acetyl-3-fluoropiperidin-4-yl)-4-chloro-*N*-(3-fluoro-5-(phenylethynyl)­pyridin-2-yl)-1*H*-pyrazole-5-carboxamide (**16f**)

Compound **16f** was prepared using a similar procedure described for compound **10**. Compound **16f** was obtained as an off-white solid (2 mg, 72% yield over 2 steps). ESI-MS [M + Na]^+^: 506.2; LCMS Retention time: 1.15 min (method (ii)); ^1^H NMR (600 MHz, DMSO-*d*_6_) δ 11.38 (s, 1H), 8.50 (s, 1H), 8.13 (d, *J* = 10.2 Hz, 1H), 7.85 (s, 1H), 7.61–7.64 (m, 2H), 7.47–7.50 (m, 3H), 4.68–5.05 (m, 2.5H), 4.39–4.41 (m, 0.5H), 4.19–4.21 (m, 0.5H), 3.88–3.90 (m, 0.5H), 3.15–3.50 (m, 1H), 2.71–2.87 (m, 1H), 1.84–2.16 (m, 5H).

#### 1-((3*R*,4*R*)-1-(*tert*-Butoxycarbonyl)-3-fluoropiperidin-4-yl)-4-chloro-1*H*-pyrazole-5-carboxylic Acid (**22**)

A mixture of methyl 4-chloro-1*H*-pyrazole-5-carboxylate (15.0 g, 93.4 mmol), *tert*-butyl (3*R*,4*S*)-3-fluoro-4-hydroxy-piperidine-1-carboxylate (24.6 g, 112 mmol) and cyanomethylenetributylphosphorane (CMBP, 33.8 g, 140 mmol) in toluene (120 mL) was stirred at 90 °C. After 7.5 h, the reaction mixture was concentrated in vacuo and crude product was purified via silica gel chromatography using 95:5 to 70:30 hexanes:ethyl acetate to elute the pure ester intermediate, which was obtained as an off-white powder (26.3 g). The intermediate ester was dissolved in THF (50 mL) and MeOH (50 mL), then added 2 mol/L sodium hydroxide (92 mL) and stirred for 4 h at rt. Upon completion of the reaction, the solution was acidified via slow addition of 2 mol/L HCl (132 mL), then product was extracted with ethyl acetate. The organics were washed with brine, dried over sodium sulfate and concentrated in vacuo to afford **22** as an off-white powder (25.1 g, 78% yield over 2 steps). ES-MS [M+H-^*t*^Bu]+: 294.2, 292.2; LCMS Retention time: 1.10 min (method (ii)); ^1^H NMR (400 MHz, DMSO-*d*_6_) δ 7.85 (s, 1H), 5.35–5.50 (m, 1H), 4.70–4.87 (m, 1H), 4.10–4.40 (m, 1H), 3.97 (brd, *J* = 11.6 Hz, 1H), 2.70–3.30 (br, 4H), 2.05–2.07 (m, 1H), 1.80–1.95 (m, 1H), 1.42 (s, 9H).

#### *tert*-Butyl (3*R*,4*R*)-4-(4-Chloro-5-((5-((4-fluorophenyl)­ethynyl)-3-methylpyridin-2-yl)­carbamoyl)-1*H*-pyrazol-1-yl)-3-fluoropiperidine-1-carboxylate (**23**)

A mixture of compound **22** (20.0 g, 57.5 mmol) and **19** (14.31 g, 63.3 mmol) in NMP (200 mL) was added NMM (13 mL, 115 mmol) and Chloro-*N*,*N*,*N*′,*N*′-tetramethylformamidiniumhexafluorophosphate (TCFH, 24.2 g, 86.3 mmol), then stirred at rt for 2.5 h. Ethyl acetate (350 mL), H_2_O (400 mL) and 1 mol/L aqueous HCl (50 mL) were added to the reaction mixture and shaken. The aqueous layer was extracted with additional ethyl acetate (50 mL), and the combined organic layer was washed with water, NaHCO_3_ aq. and brine sequentially. The Organic layer was dried over sodium sulfate and concentrated in vacuo to afford a brown oil. Methanol (90 mL) was added to the crude product, stirred, and the resulting precipitate was collected by filtration. The obtained product was washed with methanol and hexanes, and dried under reduced pressure to give compound **23** as a white powder (21.75 g, 68% yield). ES-MS [M + H]^+^: 556.5; LCMS Retention time: 1.37 min (method (ii)); ^1^H NMR (400 MHz, DMSO-*d*_6_) δ 11.03 (s, 1H), 8.44 (d, *J* = 1.6 Hz, 1H), 7.94 (d, *J* = 1.6 Hz, 1H), 7.80 (s, 1H), 7.63–7.65 (m, 2H), 7.28–7.31 (m, 2H), 4.79–4.90 (m, 2H), 4.26–4.35 (m, 1H), 3.86–4.10 (m, 1H), 2.75–3.20 (m, 2H), 2.27 (s, 3H), 2.10–2.30 (m, 1H), 1.80–2.15 (m, 1H), 1.42 (s, 9H).

#### 4-Chloro-*N*-[5-[2-(4-fluorophenyl)­ethynyl]-3-methyl-2-pyridyl]-1-[(3*R*,4*R*)-3-fluoro-4-piperidyl]­pyrazole-5-carboxamide (**24**)

To a suspension of compound **23** (22.5 g, 40.5 mmol) in MeOH (169 mL) was added methanesulfonic acid (10.5 mL, 162 mmol), and the mixture was stirred at 55 °C for 2 h. After cooled to room temperature, ethyl acetate, water, and saturated aqueous sodium bicarbonate solution were added, and the mixture was stirred. The pH of the aqueous layer was adjusted to 9, and extraction was performed with ethyl acetate. The combined organic layer was washed with brine and dried over anhydrous sodium sulfate. The solvent was evaporated under reduced pressure, and hexane/ethyl acetate (2/1) was added to the obtained oil, and the mixture was stirred for 30 min. The resulting solid was collected by filtration to give compound **24** as a white solid (17.69 g, 95% yield). ES-MS [M + H]^+^: 456.4; LCMS Retention time: 1.03 min (method (ii)); ^1^H NMR (400 MHz, DMSO-*d*_6_) δ 11.00 (s, 1H), 8.45 (d, *J* = 2.0 Hz, 1H), 7.95 (d, *J* = 2.0 Hz, 1H), 7.79 (s, 1H), 7.63–7.68 (m, 2H), 7.28–7.34 (m, 2H), 4.60–4.90 (m, 2H), 3.25–3.40 (m, 2H), 2.75–3.95 (d, J = 12.0 Hz, 1H), 2.30–2.65 (m, 2H), 2.27 (s, 3H), 1.80–2.15 (m, 2H).

#### 1-((3*R*,4*R*)-1-Acetyl-3-fluoropiperidin-4-yl)-4-chloro-*N*-(5-((4-fluorophenyl)­ethynyl)-3-methylpyridin-2-yl)-1*H*-pyrazole-5-carboxamide (**11**, **ONO-7927846**)

##### Step A: (**22**) 1-((*3R*,*4R*)-1-(*tert*-Butoxycarbonyl)-3-fluoropiperidin-4-yl)-4-chloro-1*H*-pyrazole-5-carboxylic Acid

A mixture of methyl 4-chloro-1*H*-pyrazole-5-carboxylate (15.0 g, 93.4 mmol), *tert*-butyl (3*R*,4*S*)-3-fluoro-4-hydroxy-piperidine-1-carboxylate (24.6 g, 112 mmol) and cyanomethylenetributylphosphorane (CMBP, 33.8 g, 140 mmol) in toluene (120 mL) was stirred at 90 °C. After 7.5 h, the reaction mixture was concentrated in vacuo and crude product was purified via silica gel chromatography using 95:5 to 70:30 hexanes:ethyl acetate to elute the pure ester intermediate, which was obtained as an off-white powder (26.3 g). The intermediate ester was dissolved in THF (50 mL) and MeOH (50 mL), then added 2 mol/L sodium hydroxide (92 mL) and stirred for 4 h at rt. Upon completion of the reaction, the solution was acidified via slow addition of 2 mol/L HCl (132 mL), then product was extracted with ethyl acetate. The organics were washed with brine, dried over sodium sulfate and concentrated in vacuo to afford **22** as an off-white powder (25.1 g, 78% yield over 2 steps). ES-MS [M+H-^*t*^Bu]^+^: 294.2, 292.2; LCMS Retention time: 1.10 min (method (ii)); ^1^H NMR (400 MHz, DMSO-*d*_6_) δ 7.85 (s, 1H), 5.35–5.50 (m, 1H), 4.70–4.87 (m, 1H), 4.10–4.40 (m, 1H), 3.97 (brd, *J* = 11.6 Hz, 1H), 2.70–3.30 (br, 4H), 2.05–2.07 (m, 1H), 1.80–1.95 (m, 1H), 1.42 (s, 9H).

##### Step B: (**23**) *tert*-Butyl (*3R*,*4R*)-4-(4-Chloro-5-((5-((4-fluorophenyl)­ethynyl)-3-methylpyridin-2-yl)­carbamoyl)-1*H*-pyrazol-1-yl)-3-fluoropiperidine-1-carboxylate

A mixture of compound **22** (20.0 g, 57.5 mmol) and **19** (14.31 g, 63.3 mmol) in NMP (200 mL) was added NMM (13 mL, 115 mmol) and Chloro-*N*,*N*,*N*′,*N*′-tetramethylformamidiniumhexafluorophosphate (TCFH, 24.2 g, 86.3 mmol), then stirred at rt for 2.5 h. Ethyl acetate (350 mL), H_2_O (400 mL) and 1 mol/L aqueous HCl (50 mL) were added to the reaction mixture and shaken. The aqueous layer was extracted with additional ethyl acetate (50 mL), and the combined organic layer was washed with water, NaHCO_3_ aq. and brine sequentially. The Organic layer was dried over sodium sulfate and concentrated in vacuo to afford a brown oil. Methanol (90 mL) was added to the crude product, stirred, and the resulting precipitate was collected by filtration. The obtained product was washed with methanol and hexanes, and dried under reduced pressure to give compound **23** as a white powder (21.75 g, 68% yield). ES-MS [M + H]^+^: 556.5; LCMS Retention time: 1.37 min (method (ii)); ^1^H NMR (400 MHz, DMSO-*d*_6_) δ 11.03 (s, 1H), 8.44 (d, *J* = 1.6 Hz, 1H), 7.94 (d, *J* = 1.6 Hz, 1H), 7.80 (s, 1H), 7.63–7.65 (m, 2H), 7.28–7.31 (m, 2H), 4.79–4.90 (m, 2H), 4.26–4.35 (m, 1H), 3.86–4.10 (m, 1H), 2.75–3.20 (m, 2H), 2.27 (s, 3H), 2.10–2.30 (m, 1H), 1.80–2.15 (m, 1H), 1.42 (s, 9H).

##### Step C: (**24**) 4-Chloro-*N*-[5-[2-(4-fluorophenyl)­ethynyl]-3-methyl-2-pyridyl]-1-[(3*R*,4*R*)-3-fluoro-4-piperidyl]­pyrazole-5-carboxamide

To a suspension of compound **23** (22.5 g, 40.5 mmol) in MeOH (169 mL) was added methanesulfonic acid (10.5 mL, 162 mmol), and the mixture was stirred at 55 °C for 2 h. After cooled to room temperature, ethyl acetate, water, and saturated aqueous sodium bicarbonate solution were added, and the mixture was stirred. The pH of the aqueous layer was adjusted to 9, and extraction was performed with ethyl acetate. The combined organic layer was washed with brine and dried over anhydrous sodium sulfate. The solvent was evaporated under reduced pressure, and hexane/ethyl acetate (2/1) was added to the obtained oil, and the mixture was stirred for 30 min. The resulting solid was collected by filtration to give compound **24** as a white solid (17.69 g, 95% yield). ES-MS [M + H]^+^: 456.4; LCMS Retention time: 1.03 min (method (ii)); ^1^H NMR (400 MHz, DMSO-*d*_6_) δ 11.00 (s, 1H), 8.45 (d, *J* = 2.0 Hz, 1H), 7.95 (d, *J* = 2.0 Hz, 1H), 7.79 (s, 1H), 7.63–7.68 (m, 2H), 7.28–7.34 (m, 2H), 4.60–4.90 (m, 2H), 3.25–3.40 (m, 2H), 2.75–3.95 (d, J = 12.0 Hz, 1H), 2.30–2.65 (m, 2H), 2.27 (s, 3H), 1.80–2.15 (m, 2H).

##### Step D: (**11**, **ONO-7927846**) 1-((3*R*,4*R*)-1-Acetyl-3-fluoropiperidin-4-yl)-4-chloro-*N*-(5-((4-fluorophenyl)­ethynyl)-3-methylpyridin-2-yl)-1*H*-pyrazole-5-carboxamide

To a suspension of compound **24** (17.56 g, 38.5 mmol) and pyridine (4.7 mL, 57.8 mmol) in MeCN (176 mL) was added acetic anhydride (4.37 mL, 46.2 mmol) at 15 to 20 °C, and the mixture was stirred at rt for 1 h. Saturated aqueous sodium hydrogen carbonate solution was added to the reaction mixture, and the mixture was stirred at room temperature for 15 min. Ethyl acetate, water, and 1 mol/L aqueous HCl solution were added, the mixture was shaken and separated. The organic layer was successively washed with dilute hydrochloric acid, saturated aqueous sodium bicarbonate solution and brine, and dried over anhydrous sodium sulfate. The mixture was concentrated under reduced pressure to obtain a yellow amorphous form. To this crude product, methanol (600 mL) and water (300 mL) were added, and the mixture was dissolved by heating at 70 °C. The temperature was gradually lowered to room temperature, and the precipitated solid was collected by filtration to obtain the target substance **11** (**ONO-7927846**) as a white solid (16.64 g, 87% yield). ES-MS [M + H]^+^: 498.3; LCMS Retention time: 1.17 min (method (ii)); ^1^H NMR (600 MHz, DMSO-*d*_6_) δ 11.02 (s, 1H), 8.44 (s, 1H), 7.94 (d, *J* = 1.2 Hz, 1H), 7.80 (s, 1H), 7.63–7.65 (m, 2H), 7.28–7.31 (m, 2H), 4.67–5.05 (m, 2.5H), 4.37–4.39 (m, 0.5H), 4.18–4.21 (m, 0.5H), 3.86–3.88 (m, 0.5H), 3.28–3.33 (m, 0.5H), 3.21–3.26 (m, 0.5H), 2.84–2.89 (m, 0.5H), 2.73–2.77 (m, 0.5H), 2.27 (s, 3H), 2.03–2.17 (m, 4.5H), 1.84–1.91 (m, 0.5H); ^13^C NMR (150 MHz, DMSO-*d*_6_) δ 168.78 (d, *J* = 10.4 Hz), 162.24 (d, *J* = 246.0 Hz), 157.34, 148.74, 148.06, 141.61, 137.57, 135.09, 133.84 (d, *J* = 8.1 Hz), 128.46, 118.24 (d, *J* = 3.5 Hz), 117.38, 116.14 (d, *J* = 21.8 Hz), 109.25, 91.13, 88.18 (d, *J* = 180.5 Hz), 87.73 (d, *J* = 180.5 Hz), 85.65, 61.15 (d, *J* = 17.8 Hz), 61.13 (d, *J* = 17.8 Hz), 47.75 (d, *J* = 28.2 Hz), 43.72, 42.92 (d, *J* = 28.2 Hz), 38.89, 30.49 (d, *J* = 5.7 Hz), 29.56 (d, *J* = 5.7 Hz), 21.20 and 21.09, 17.32; HRMS: Obs: 498.1509, Calcd: 498.1503 for C_25_H_23_ClF_2_N_5_O_2_ [M + H]^+^; mp 104–107 °C.

## Supplementary Material





## Notes

Additional experimental details, characterization of all compounds (^1^H NMR, ^13^C NMR, 2D NMR, HRMS), *in vitro* and *in vivo* DMPK protocols (PDF)
Molecular string formulas (CSV)

## Abbreviations Used

MED: minimum effective dose
NOAEL: no observed adverse effect level
NOR: novel object recognition
PBL: plasma:brain level study
PK: pharmacokinetics
TREK: TWIK RElated K^+^ channels
