# Supplementary material for: Optimization of Selective and CNS Penetrant Alkyne-Based TREK Inhibitors: The Discovery and Characterization of ONO-9517601 (VU6022856) and ONO-7927846 (VU6024391)
Source: J Med Chem. 2025 Oct 17;68(21):23554–72. doi: 10.1021/acs.jmedchem.5c02535 (PMC12621189; doi:10.1021/acs.jmedchem.5c02535)
Supplement: Supplementary file 1 [file jm5c02535_si_001.pdf]

**Supporting Information**

# Optimization of selective and CNS penetrant alkyne-based TREK inhibitors: the discovery and characterization of ONO-9517601 (VU6022856) and ONO-7927846 (VU6024391)

Motoyuki Tanaka,[1] Yoko Sekioka,[2] Gakuji Hashimoto,[2] Takahiro Mori,[2] Tomoyuki Shono,[3] Yuuki Isaji,[4] Katsuya Hisaichi,[1] Elizabeth S. Childress,[5,6] Sean Bollinger,[5,6] Joza A. Schmitt,[5,6] Trevor C. Chopko,[5,6] Aaron T. Garrison,[5,6] Charles K. Perry,[5,6] Keagan Chronister,[5,6] Meghan Kramer,[5,6] Sichen Chang,[5,6] Katherine J. Watson,[5,6] Jonathan W. Dickerson,[5,6] Michael Bubser,[5,6] Jerri M. Rook,[5,6] Carrie K. Jones,[5,6] Olivier Boutaud,[5,6] Thomas M. Bridges,[5,6] Jerod S. Denton,[5,6,7] Darren W. Engers,[5,6] Haruto Kurata*[1] and Craig W. Lindsley*[5,6]

**Affiliation:**

[1]Drug Discovery Chemistry, Ono Pharmaceutical Co., Ltd, 3-1-1 Sakurai, Shimamoto, Mishima, Osaka 618-8585, Japan

[2]Research Center of Neurology, Ono Pharmaceutical Co., Ltd, 3-1-1 Sakurai, Shimamoto, Mishima, Osaka 618-8585, Japan

[3]Pharmacokinetic Research, Ono Pharmaceutical Co., Ltd, 3-1-1 Sakurai, Shimamoto, Mishima, Osaka 618-8585, Japan

[4]Safety Research, Ono Pharmaceutical Co., Ltd, 3-1-1 Sakurai, Shimamoto, Mishima, Osaka 618-8585, Japan

[5]Warren Center for Neuroscience Drug Discovery, Vanderbilt University, Nashville, TN 37232, USA

[6]Department of Pharmacology, Vanderbilt University School of Medicine, Nashville, TN 37232, USA

[7]Department of Anesthesiology, Vanderbilt University Medical Center, Nashville, TN 37232, USA

*To whom correspondence should be addressed at craig.lindsley@vanderbilt.edu, h.kurata@ono-pharma.com

## Table of Contents

**Procedure for Biological Experiments……………………………………………………..S2**

**$^{1}$H NMR and $^{13}$C NMR spectra for ONO-9517601 and ONO-7927846…………………...S11**

## Procedures for Biological Experiments

**TREK-1 Thallium flux assay**

**Method 1**;

CHO-K1 cells stably expressing hTREK-1 were cultured in T225 flask. The cells were loaded with FluxOR dye and plated in 384 well plate at the experiment day. Test compounds or control compound (tert-butyl (3-((4-(benzyloxy)-2-methylphenyl)carbamoyl)-4-chlorophenyl)carbamate) or 0.3% DMSO (vehicle control) were added directly to the cell plates and incubated for 10 min, and then treated with thallium stimulus buffer to initiate thallium flux. To measure the efficacy and potency of test compounds, the change in fluorescence intensity (ΔRatio) and % inhibition were calculated using the following equations:

ΔRatio = (fluorescence intensity at 25 seconds after thallium addition)/(average of fluorescent intensity before thallium addition)

% inhibition = {1 - (ΔRatio of test compound - ΔRatio of 10 μM control compound)/(ΔRatio of 0.3% DMSO - ΔRatio of 10 μM control compound)}x100

% inhibition vs compound concentration were plotted in XLfit and calculated $IC_{50}$.

**Method 2**;

CHO-K1 cells stably expressing human TREK-1 (hTREK-1) were cultured in T225 flask. The cells were loaded with FluxOR dye and plated in 384 well plate at the experiment day. Test compounds or control compound (tert-butyl (3-((4-(benzyloxy)-2-methylphenyl)carbamoyl)-4-chlorophenyl)carbamate) or 0.3% DMSO (vehicle control) which were prepared on separate plates were added to the cell plates and incubated for 10 min, and then the cells with test compounds or control compound (tert-butyl (3-((4-(benzyloxy)-2-methylphenyl)carbamoyl)-4-chlorophenyl)carbamate) or 0.3% DMSO (vehicle control) were treated with thallium stimulus buffer to initiate thallium flux. To measure the efficacy and potency of test compounds, the change in fluorescence intensity (ΔRatio) and % inhibition were calculated using the following equations:

ΔRatio = (fluorescence intensity at 25 seconds after thallium addition)/(average of fluorescent intensity before thallium addition)

% inhibition = {1 - (ΔRatio of test compound - ΔRatio of 10 μM control compound)/(ΔRatio of 0.3% DMSO - ΔRatio of 10 μM control compound)}x100

% inhibition vs compound concentration were plotted in XLfit and calculated $IC_{50}$.

**TREK-2 Thallium flux assay**

HEK293 cells stably expressing human TREK-2 (hTREK-2) are plated in 384-well plates, cultured overnight, loaded with Thallos dye the following day. Test compounds or control compound (tert-butyl (3-((4-(benzyloxy)-2-methylphenyl)carbamoyl)-4-chlorophenyl)carbamate) or 0.3% DMSO (vehicle control) which are prepared on separate plates are added to the cell plates and incubated for 10 min, and then the cells were treated with thallium stimulus buffer to initiate thallium flux.

To measure the efficacy and potency of test compounds, the change in fluorescence intensity (ΔRatio) and % inhibition are calculated using the following equations:

ΔRatio = (fluorescence intensity at 25 seconds after thallium addition)/(average of fluorescent intensity before thallium addition)

% inhibition = {1 - (ΔRatio of test compound - ΔRatio of 10 μM control compound)/(ΔRatio of 0.3% DMSO - ΔRatio of 10 μM control compound)}x100

**Patch clamp technique**

**hTREK-1 and mTREK-1 patch clamp assay**

CHO-K1 cells stably expressing hTREK-1 or CHO-K1 cells transiently expressing mouse TREK-1 (mTREK-1) were plated on glass coverslips, and voltage clamped in the whole-cell configuration of the patch clamp technique. Cells were voltage clamped at a holding potential of -80 mV and the stepped to 0 mV for 500 msec. The voltage was subsequently ramped from -120mV to +80 mV over a 500 msec duration. This step-ramp protocol was repeated every 10 sec. The bathing solution contained the following: 135 mM NaCl, 5 mM KCl, 2 mM $CaCl_2$, 1 mM $MgCl_2$, 5 mM D-Glucose, 10 mM HEPES, 10 mM sucrose (adjusted to pH 7.4 with NaOH, 300 mosmol/kg $H_2O$). The pipette solution contained the following: 135 mM KCl, 2 mM $MgCl_2$, 1 mM EGTA, 10 mM HEPES, 2 mM $Na_2ATP$ (adjusted to pH 7.35 with KOH, 285 mosmol/kg $H_2O$). Test

compounds were dissolved into the bathing solution. The effects of test compound on the currents were calculated at 0 mV using the following equations:

% inhibition = (1-post current/pre current) x 100

% inhibition vs compound concentrations were plotted in GraphPad Prism and calculated $IC_{50}$.

It was found that each compound of present invention has a prominent TREK-1 inhibitory activity.

**hTREK-2 patch clamp assay**

HEK293 cells stably expressing human TREK-2 are plated on glass coverslips, and voltage clamped in the whole-cell configuration of the patch clamp technique. Cells are voltage clamped at a holding potential of -80 mV and the stepped to 0 mV for 500 msec. The voltage is subsequently ramped from -120mV to +80 mV over a 500 msec duration. This step-ramp protocol is repeated every 10 sec. The bathing solution contained the following: 135 mM NaCl, 5 mM KCl, 2 mM $CaCl_2$, 1 mM $MgCl_2$, 5 mM D-Glucose, 10 mM HEPES, 10 mM sucrose (adjusted to pH 7.4 with NaOH, 300 mosmol/kg $H_2O$). The pipette solution contained the following: 135 mM KCl, 2 mM $MgCl_2$, 1 mM EGTA, 10 mM HEPES, 2 mM $Na_2ATP$ (adjusted to pH 7.35 with KOH, 285 mosmol/kg $H_2O$). Test compounds are dissolved into the bathing solution. Experiments are terminated with the addition of the control compound (tert-butyl (3-((4-(benzyloxy)-2-methylphenyl)carbamoyl)-4-chlorophenyl)carbamate) so that maximal inhibition can be determined. The effects of test compound on the currents can be calculated at 0 mV using the following equations:

% inhibition = {1 - (post current - current in the presence of 10 μM control compound) / (pre current - current in the presence of 10 μM control compound)} x 100

**Drug Metabolism Methods:**

***In vitro***

**Plasma protein binding and Brain homogenate binding:** Determination of fraction unbound ($f_u$) in plasma was conducted in vitro via equilibrium dialysis using HTDialysis (HTD) membrane plates. The top half of the plate was filled with 100 uL of Dubelco's Phosphate Buffered Saline, pH 7.4 (DPBS). Compounds were diluted into plasma from each species (5 μM final

concentration), which was aliquoted in triplicate to the 'bottom half' of the prepared HTD plate wells. The HTD plate was sealed and incubated for 6 hours at 37 ˚C. Following incubation, each well (both top and bottom halves) were transferred (20 µL) to the corresponding wells of a 96-shallow-well (V-bottom) plate. The daughter plates were then matrix-matched (DPBS side wells received equal volume of plasma, and plasma side wells received equal volume of DPBS), and extraction solution (120 µL; acetonitrile containing 50 nM carbamazepine as IS) was added to all wells of both daughter plates to precipitate protein and extract test article. The plates were then sealed and centrifuged (3500 rcf) for 10 minutes at ambient temperature. Supernatant (60 µL) from each well of the daughter plates was then transferred to the corresponding wells of new daughter plates (96-shallow-well, V bottom) containing water (Milli-Q, 60 µL/well), and the plates were sealed in preparation for LC-MS/MS analysis (see LC-MS/MS analysis method below).

The unbound fraction ($f_u$) was calculated following the equation below, and mean values for each species were calculated from 3 replicates.

A similar approach was used to determine the degree of brain homogenate binding, which employed the same methodology and procedure with the following modifications: 1) a final compound concentration of 1 µM was used, 2) naïve rat brains were homogenized in DPBS (1:3 composition of brain: DPBS, w/w) using a Mini-Bead Beater™ machine in order to obtain brain homogenate, which was then treated in the same manner as the plasma samples in the previously described plasma protein binding assay. Fraction unbound for both plasma and brain samples was determined using Equation 4.

$$f_u = \frac{Conc_{buffer}}{Conc_{plasma}}$$

Equation 4 Determination of fraction unbound in plasma.

The diluted fraction unbound ($f_u2$) in brain was calculated in the same manner by using brain homogenate rather than plasma. Undiluted fraction unbound for the brain was calculated using Equation 5

$$f_u = \frac{1/4}{\left\{\left(\frac{1}{fu2}\right) - 1\right\} + 1/4}$$

Equation 5 Determination of fraction unbound in brain. $F_u2$ represents the diluted fraction unbound.

**Intrinsic clearance:** Human or rat hepatic microsomes (0.5 mg/mL) and 1 μM test compound were incubated in 100 mM potassium phosphate pH 7.4 buffer with 3 mM $MgCl_2$ at 37 °C with constant shaking. After a 5 min preincubation, the reaction was initiated by the addition of NADPH (1 mM). At selected time intervals (0, 3, 7, 15, 25, and 45 min), aliquots were taken and subsequently placed into a 96-well plate containing cold acetonitrile with internal standard (50 ng/mL carbamazepine). Plates were then centrifuged at 3000 rcf (4 °C) for 10 min, and the supernatant was transferred to a separate 96-well plate and diluted 1:1 with water for LC/MS/MS analysis. The *in vitro* half-life ($_{1/2}$, min, Eq. 1), intrinsic clearance ($CL_{int}$, mL/min/kg, Eq. 2), and subsequent predicted hepatic clearance ($CL_{hep}$, mL/min/kg, Eq. 3) was determined employing the following equations:

(1) $T_{1/2} = \frac{Ln(2)}{K}$

where k represents the slope from linear regression analysis of the natural log percent remaining of a test compound as a function of incubation time

(2) $CL_{int} = \frac{0.693}{in\ vitro T_{1/2}}\ x\ \frac{mL\ incubation}{mg\ microsomes}\ x\ \frac{45\ mg\ microsomes}{gram\ liver}\ x\ \frac{20^{a}\ gram\ liver}{kg\ body\ wt}$

[a]scale-up factors: of 20 (human) or 45 (rat)

(3) $CL_{hep} = \frac{Q_h \cdot CL_{int}}{Q_h + CL_{int}}$

where $Q_h$ (hepatic blood flow, mL/min/kg) is 21 (human) or 70 (rat).

**LC/MS/MS Bioanalysis of Samples from Plasma Protein Binding and Intrinsic Clearance Assays:**

Samples were analyzed on a Thermo Electron TSQ Quantum Ultra triple quad mass spectrometer (San Jose, CA) via electrospray ionization (ESI) with two Themo Electron Accella pumps (San Jose, CA), and a Leap Technologies CTC PAL autosampler (Carrboro, NC). Analytes were

separated by gradient elution on a dual column system with two Thermo Hypersil Gold (2.1 x 30 mm, 1.9 μm) columns (San Jose, CA) thermostated at 40 °C. HPLC mobile phase A was 0.1% formic acid in water and mobile phase B was 0.1% formic acid in acetonitrile. The gradient started at 10% B after a 0.2 min hold and was linearly increased to 95% B over 0.8 min; hold at 95% B for 0.2 min; returned to 10% B in 0.1 min. The total run time was 1.3 min and the HPLC flow rate was 0.8 mL/min. While pump 1 ran the gradient method, pump 2 equilibrated the alternate column isocratically at 10% B. Compound optimization, data collection, and processing was performed using Thermo Electron's QuickQuan software (v2.3) and Xcalibur (v2.0.7 SP1).

***In vivo* DMPK experimental:**

All animal experiments were performed following the protocols evaluated and approved by the [committee name and affiliation] (Ethics Approval Number: 102215 (rat), 102214 (dog), 4day tox for ONO-792784 (102320), 4day tox for ONO-951760 (102146), the embryo-fetal developmental tox study for ONO-792784 (102597) and the embryo-fetal developmental tox study for ONO-951760 (102575).

Determination of brain to plasma ratio:

*Animal care and use*

All animal study procedures were approved by the Institutional Animal Care and Use Committee and were conducted in accordance with the National Institutes of Health regulations of animal care covered in Principles of Laboratory Animal Care (National Institutes of Health). All rats were fasted overnight prior to testing.

*In-life phase*

For determination of the brain over plasma ratio ($K_p$), compounds were formulated in 8% ethanol, 32% PEG400 and 60% DMSO (*v/v/v*) and administered as a single 0.2 mg/kg IV dose (1 mL/kg) to male, Sprague Dawley rats (*n* = 1) via injection into a surgically-implanted jugular vein catheter. At 15 min post dosing, blood sample was collected into chilled, $K_2$EDTA anticoagulant-fortified tube and immediately placed on wet ice. The blood sample was then centrifuged (1700 rcf, 5 minutes, 4 ˚C) to obtain plasma sample. At the same post-administration time point, whole

brain sample was obtained by rapid dissection, rinsed with PBS, and immediately frozen in individual tissue collection box (dry ice). All brain and plasma samples were stored at -80 ˚C until analysis by LC-MS/MS.

*Sample Analysis:* Concentrations in plasma and brain homogenates were quantified by liquid chromatography tandem mass spectrometry (LC-MS/MS). Whole brains were homogenized in 3 mL of 70:30 IPA:water in a mini bead beater for 3 min, and centrifuged at 3,500 g for 5 min. 5 uL of the supernatant was diluted in 15 uL of blank plasma for quantification of the analytes. Plasma samples were centrifuged at 3,500 g for 5 min. A standard curve was generated by diluting the analytes DMSO stocks with blank plasma to obtain a final concentration of 10,000 ng/ml followed by a serial dilution down to 0.5 ng/ml. Quality controls were generated by a serial dilution of the 5,000 ng/ml standard curve solution in blank plasma to obtain 3 concentrations of 500, 50, and 5 ng/ml. 20 uL of brain diluted in plasma, plasma, blank plasma, standard curve and QC samples were loaded in a V-bottom 96-well plate. 120 uL of acetonitrile containing 0.05 uM carbamazepine (internal standard) was added to each well and the plate was centrifuged at 3,500 g for 5 min. 60 uL of the supernatant of each well (protein free) was transferred to a new 96-well plate containing 60 uL of water. The plates were sealed for analysis by LC-MS/MS.

Plasma and brain tissue samples originating from *in vivo* studies were analyzed by electrospray ionization using an AB Sciex Q-TRAP 5500 (Foster City, CA) that was coupled to a Shimadzu LC-20AD pump (Columbia, MD) and a Leap Technologies CTC PAL auto-sampler (Carrboro, NC). Analytes were separated by gradient elution using a C18 column (3 x 50 mm, 3 mm; Fortis Technologies Ltd, Cheshire, UK) that was thermostated at 40 °C. HPLC mobile phase A was 0.1% formic acid in water (pH unadjusted); mobile phase B was 0.1% formic acid in acetonitrile (pH unadjusted). A 10% B gradient was held for 0.2 min and was linearly increased to 90% B over 0.8 min, with an isocratic hold for 0.5 min, before transitioning to 10% B over 0.05 min. The column was re-equilibrated (1 min) before the next sample injection. The total run time was 2.55 min, and the HPLC flow rate was 0.5 ml/min. The source temperature was set at 500 °C, and mass spectral analyses were performed using a Turbo-Ion spray source in positive ionization mode (5.0-kV spray voltage) and using multiple-reaction monitoring of transitions specific for the analytes. All data were analyzed using AB Sciex Analyst 1.5.1 software.

Brain plasma concentration ratio ($K_p$) was calculated by dividing brain concentration by plasma concentration for each animal. Unbound brain to unbound plasma concentration ratio ($K_{p,uu}$) is calculated using the following formula: $K_{p,uu}$ = (Brain ng/g x brain fu)/(plasma ng/ml x plasma fu).

**In-vitro determination of blood-brain barrier penetration potential**

Blood-brain barrier penetration was determined using MDR1-MDCK cell monolayers by Absorption Systems, following their protocol. In short, compounds were incubated at 5 mM final concentration on one side of the cell monolayer for 2 hours. Compounds concentration on either side of the monolayer was determined by LC-MS/MS and apparent permeability and efflux ratio were determined as described in Wang, Q. et al.[1]

**MK-801 induced novel object recognition test**

**Drugs:** Test compounds formulated in 20% Kolliphor HS 15/propylene glycol (7:3) / 80% sterile water vehicle. Following vigorous vortexing, the formulated compound was placed in an ultrasonic water bath for 1 hour. The compound formulats at a concentration that allowed for an oral (p.o.) administration of 10 mL dosing solution/kg body weight. MK-801 hydrogen maleate was obtained from Sigma-Aldrich, dissolved in sterile saline, and dosed intraperitoneally in a volume of 1 mL/kg body weight (0.2 mg/kg).

**Animals:** Adult male C57BL/6J mice (Charles River Japan) at six-week-old were used. They were housed in an animal care facility certified by the Japan Health Sciences Foundation under a 12-hour light/dark cycle (lights on: 7 a.m.; lights off: 7 p.m.) and had free access to food and water. Animals were acclimated to the housing facility for a minimum of six days before being tested and the behavioral testing is performed during the light phase. All experiments were approved by the Institutional Animals Care and Use Committee of Ono Pharmaceutical Co., Ltd.

**Apparatus:** For the novel object recognition test an opaque Plastic chamber (35 cm x 40 cm x 18 cm) was used. At opposite ends of the chamber test objects could be placed. A video camera was mounted above the apparatus for recording the behavior.

**Procedure:** Habituation. At least one day prior to behavioral testing, animals were habituated to the empty testing chamber, i.e. in the absence of any objects, for ten minutes.

Training. One hour after administration of vehicle, the compound (p.o.) or clozapine (1 mg/kg, p.o.), and 30 minutes after administration of saline or MK-801, animals are placed into the testing chamber that contained two identical objects (LEGO (registered Trademark) blocks) for a session duration of 10 minutes. Afterwards, animals were returned to their home cage.

Recognition. Ninety minutes after the end of the training session, animals were reintroduced to the test chamber where one of the two identical objects have been replaced by a novel object (glass vial) for a total of 10 minutes.

**Behavioral analysis:** An observer blinds to treatment condition and novel object location used the video recordings to score the interaction of the animal with the two objects offline. The duration an animal explored each object was determined as the total time an animal is facing the object with its nose being ≦ 2 cm away from the object and some discernible whisker movement being present. From these data, a Discrimination Index is calculated as follows:

Discrimination Index = 100 x (time exploring Novel object - time exploring Familiar object)/ (time exploring Novel object + time exploring Familiar object).

**Data Analysis:** Statistical analyses can be performed using EXSUS ver 8.1 (CAC Croit Corporation). The vehicle and compound-treated groups were analyzed by one-way ANOVA followed by Dunnett's test. The vehicle and vehicle-MK-801, and vehicle-MK-801 and clozapine groups can be analyzed by Student t-test. A p-value ≦ 0.05 is considered to represent a significant difference.

**Characterization of ONO-9517601**

**$^1$H NMR Spectra for Compound 10**

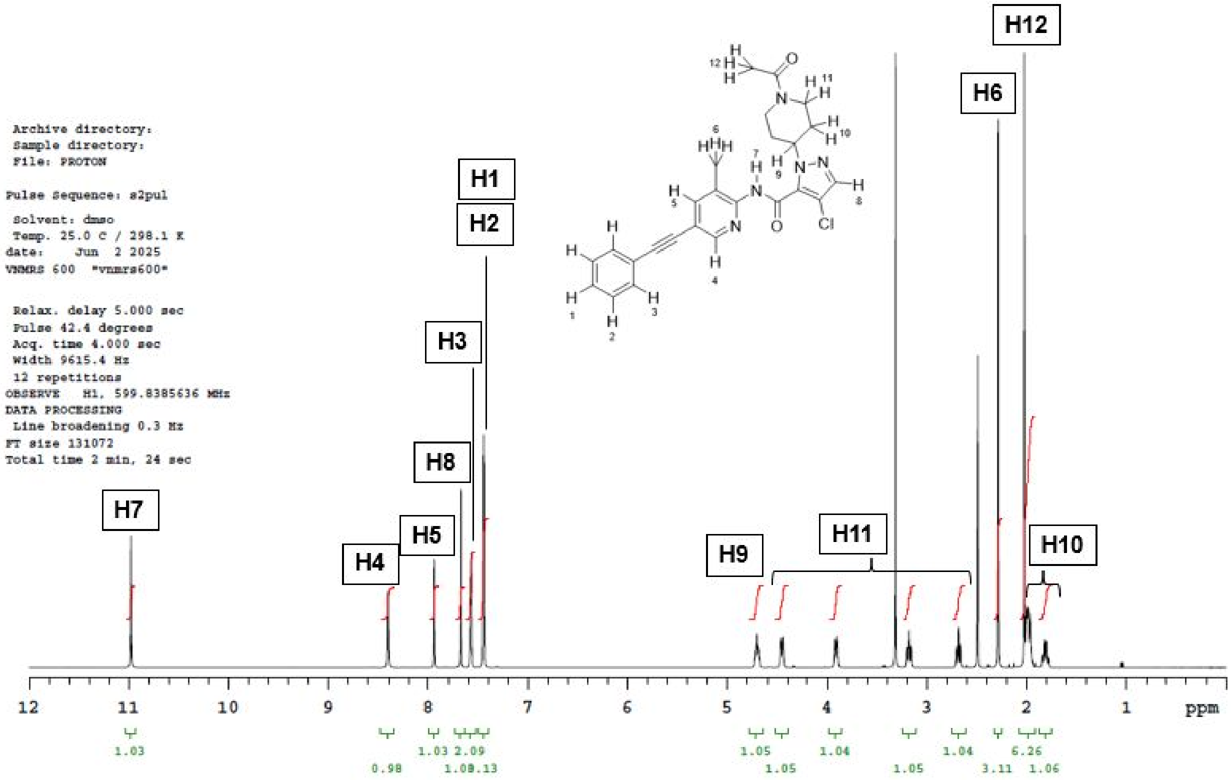

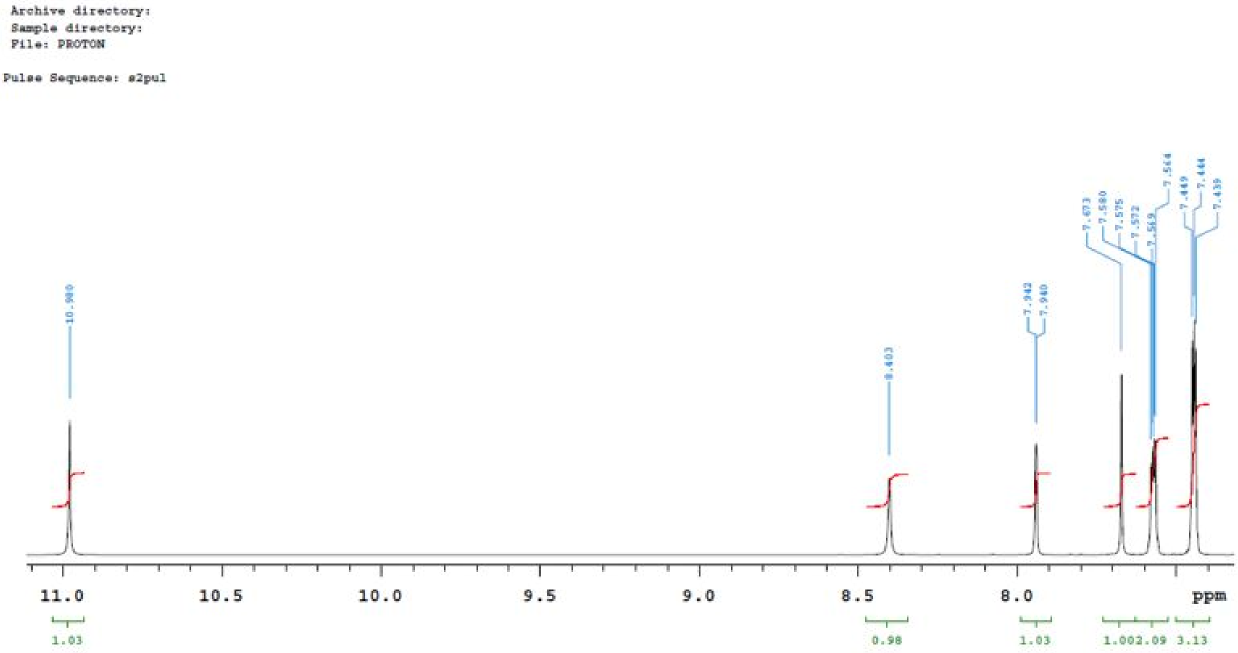
Archive directory:
Sample directory:
File: PROTON
Pulse Sequence: s2pul
ppm

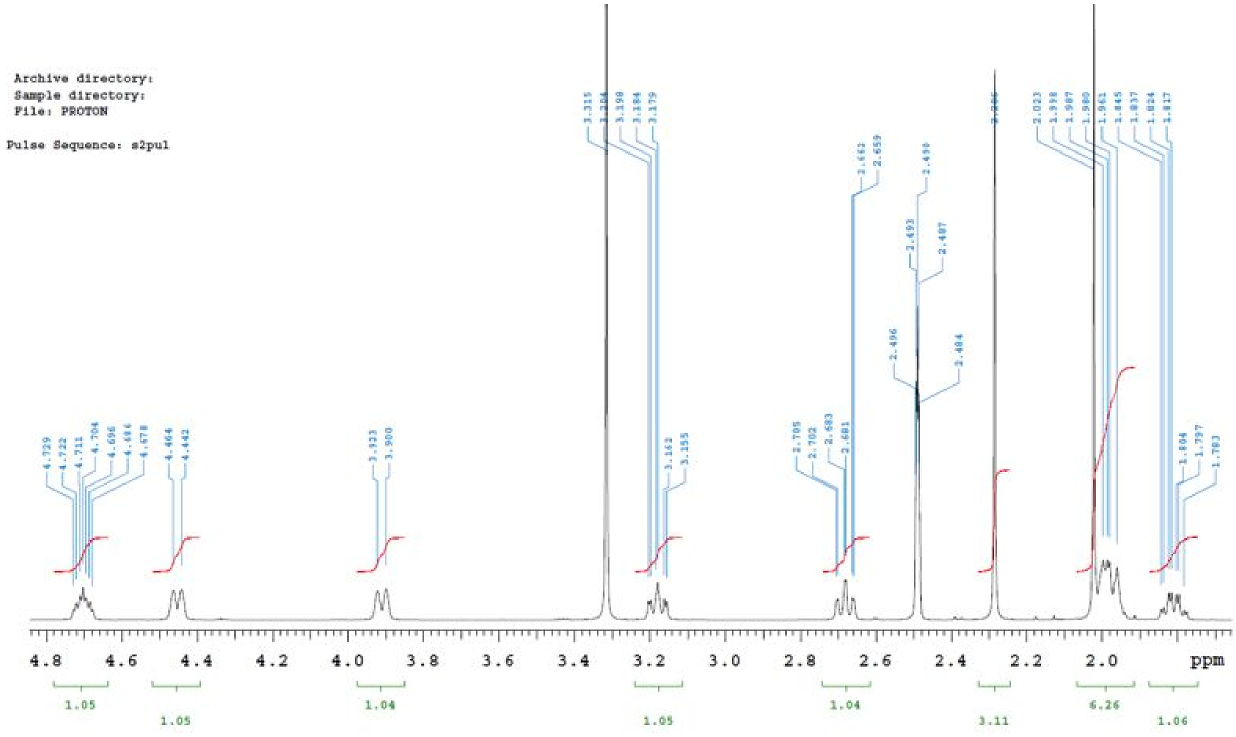
Archive directory:
Sample directory:
File: PROTON
Pulse Sequence: s2pul
ppm

## $^{13}$C NMR Spectra for Compound 10

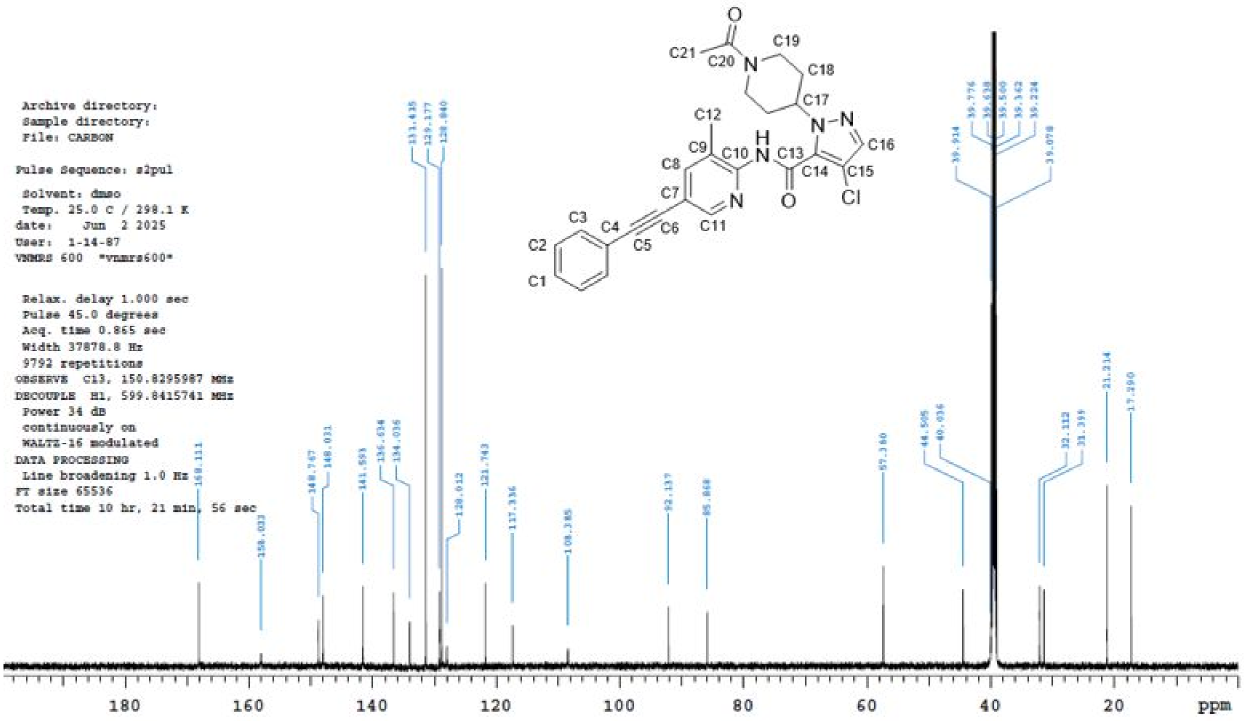

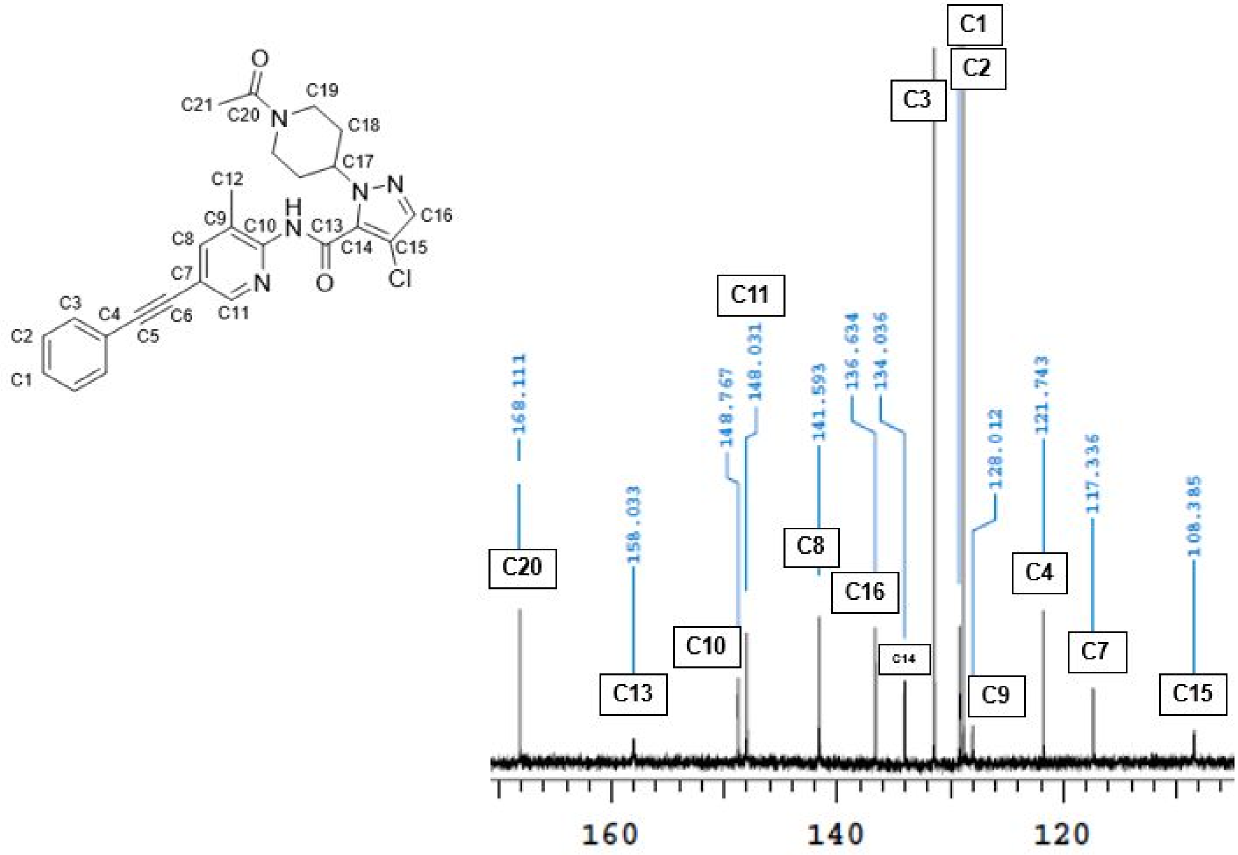
C1
C2
C3
C11
C20
C13
C10
C8
C16
C14
C9
C4
C7
C15
168.111
158.033
148.767
148.031
141.593
136.634
134.036
128.012
121.743
117.336
108.385
160
140
120
C21
C19
C18
C17
C12
C6
C5
O
N
NH
Cl

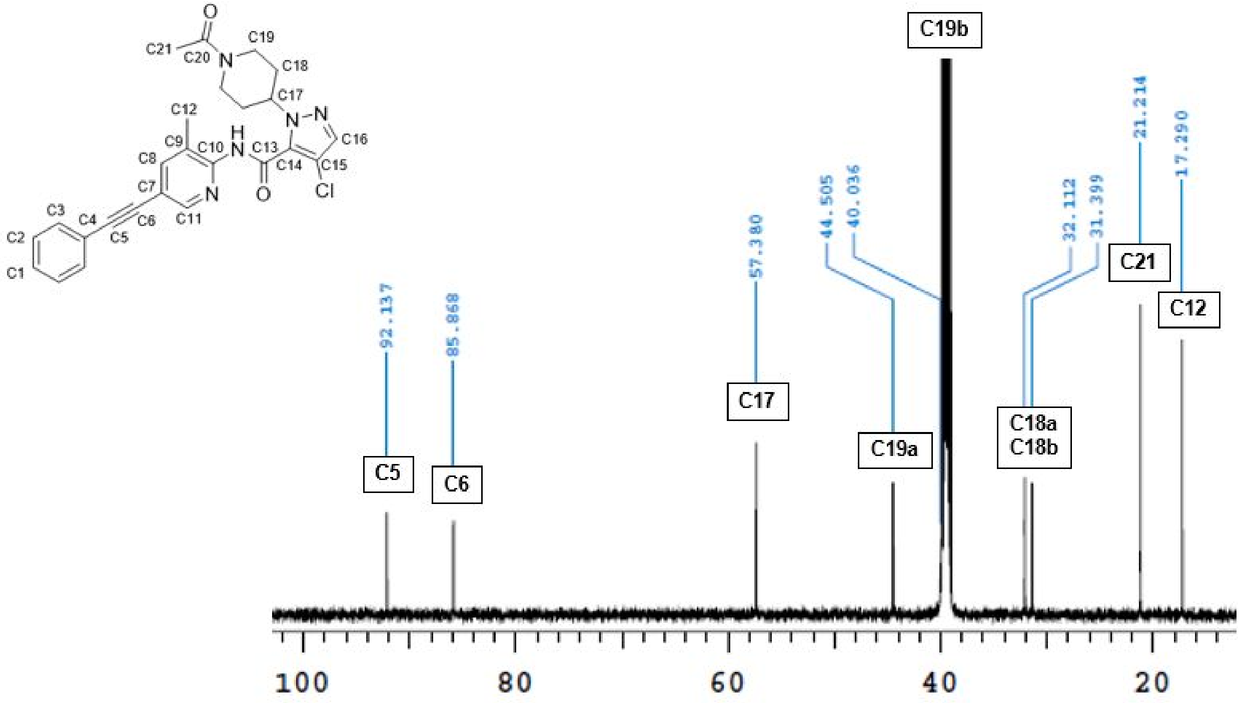
C19b
C21
C12
C17
C19a
C18a
C18b
C5
C6
92.137
85.868
57.380
44.505
40.036
32.112
31.399
21.214
17.290
100
80
60
40
20

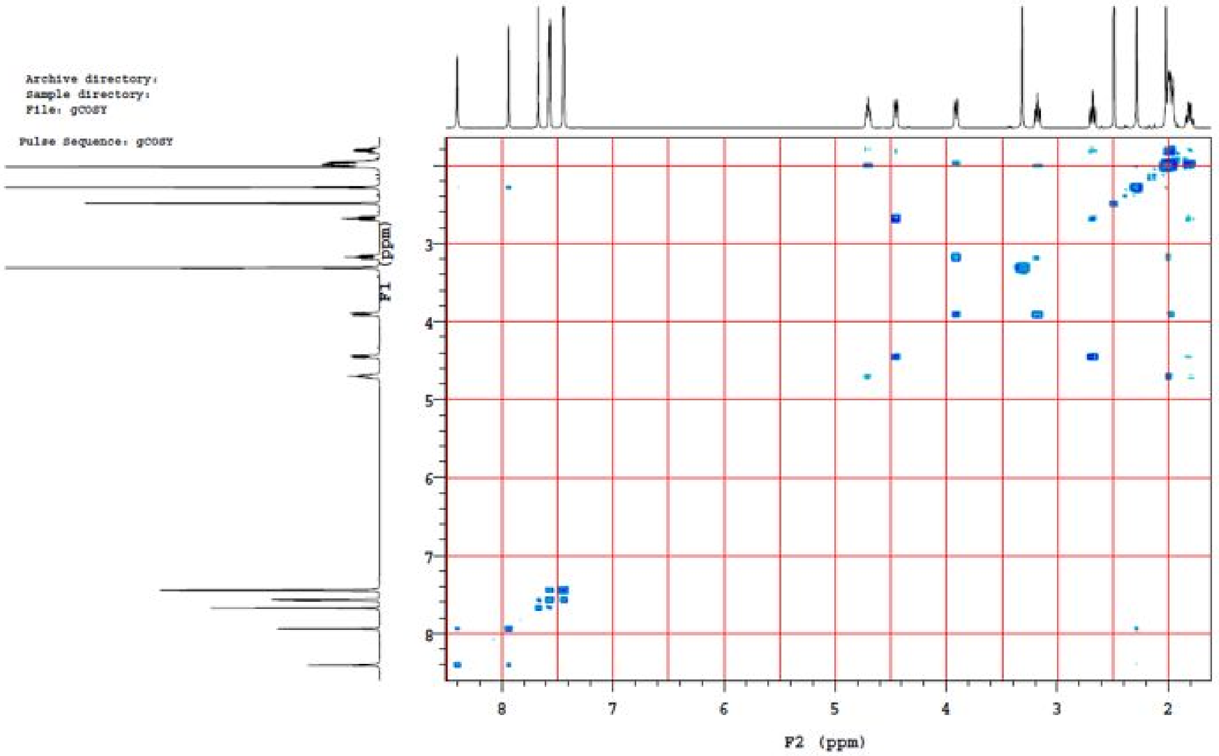
Archive directory:
Sample directory:
File: gCOSY
Pulse Sequence: gCOSY
F1 (ppm)
F2 (ppm)

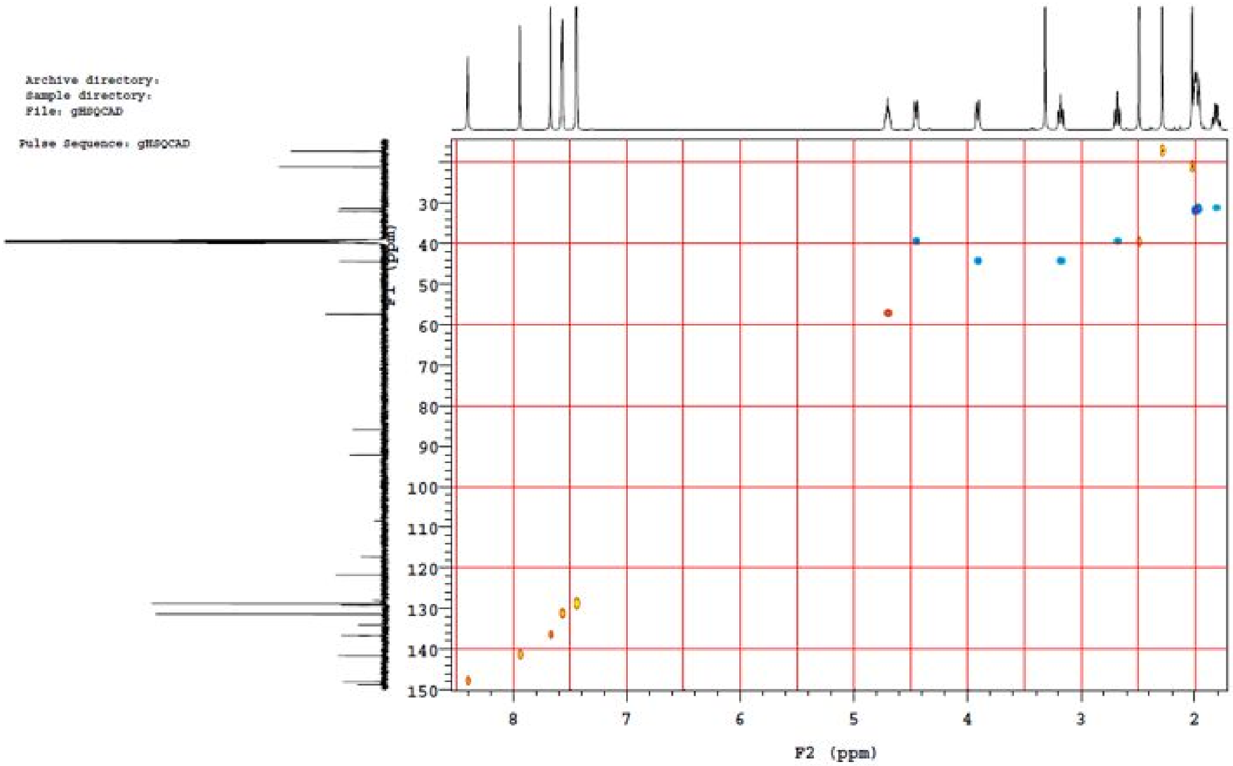
Archive directory:
Sample directory:
File: gHSQCAD
Pulse Sequence: gHSQCAD
F1 (ppm)
F2 (ppm)

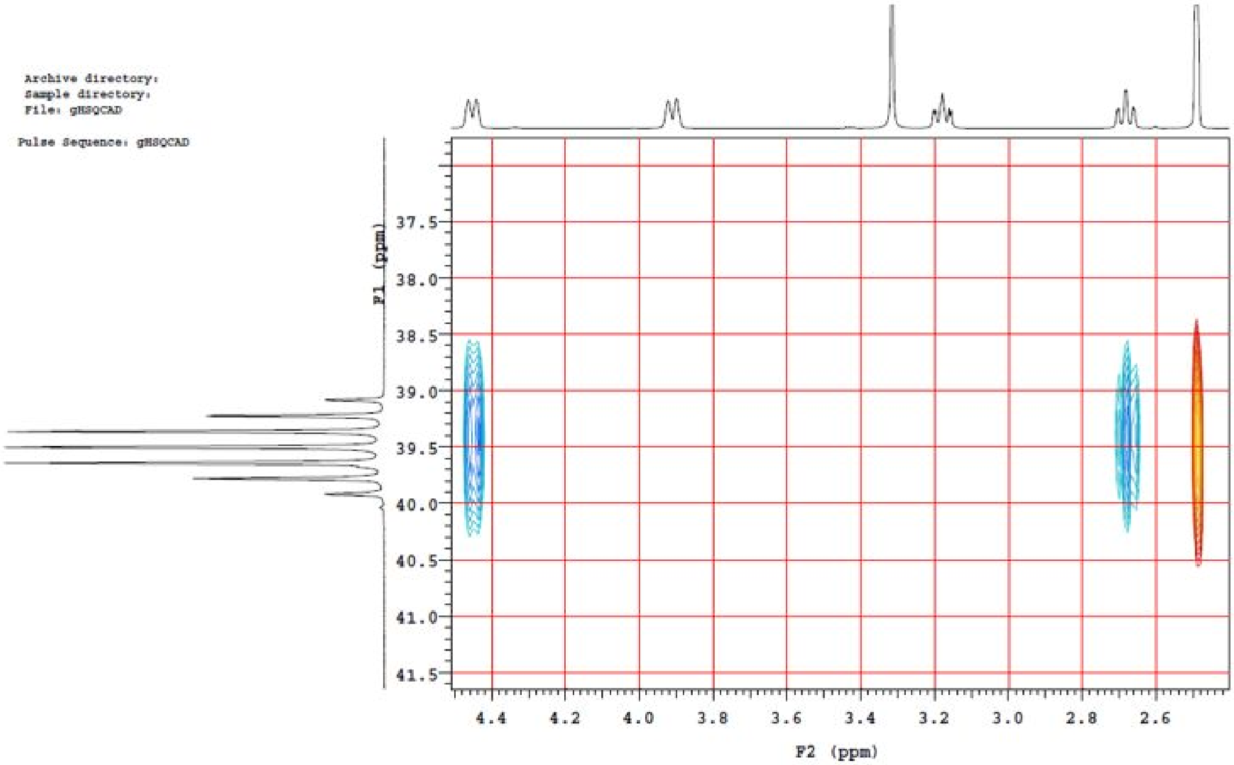
Archive directory:
Sample directory:
File: gHSQCAD
Pulse Sequence: gHSQCAD
F1 (ppm)
F2 (ppm)

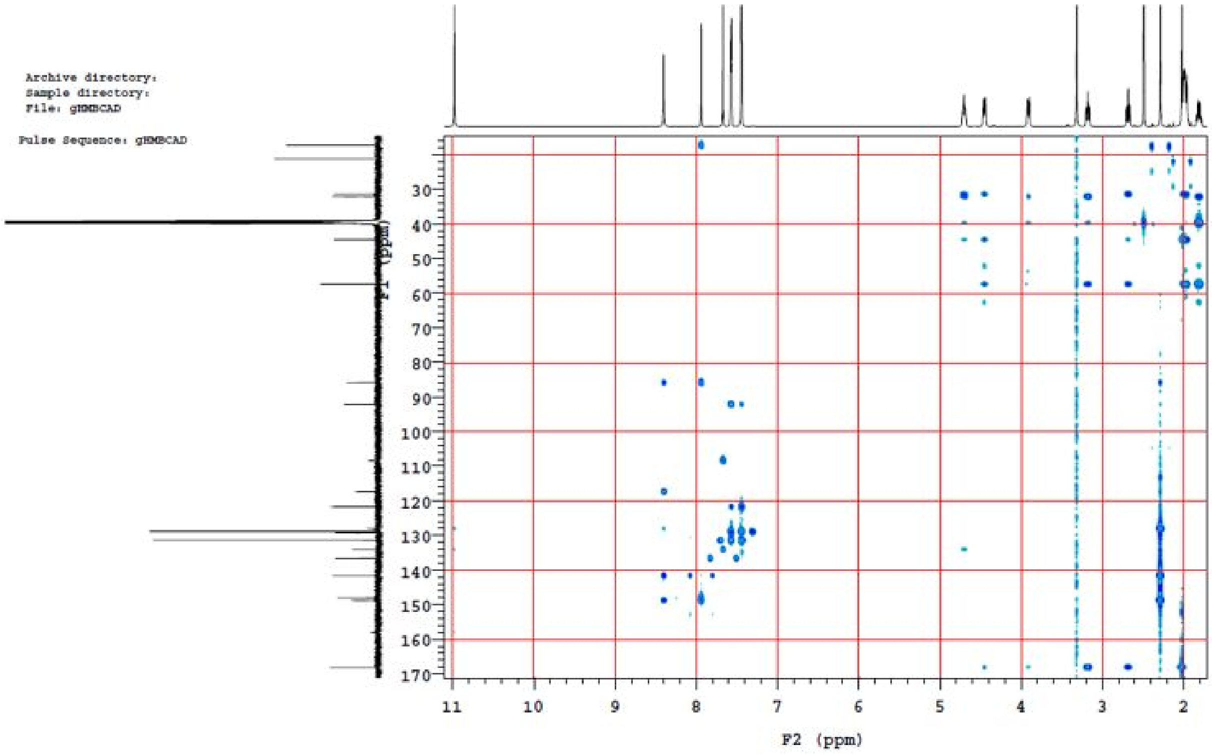
Archive directory:
Sample directory:
File: gHMBCAD
Pulse Sequence: gHMBCAD
F1 (ppm)
F2 (ppm)

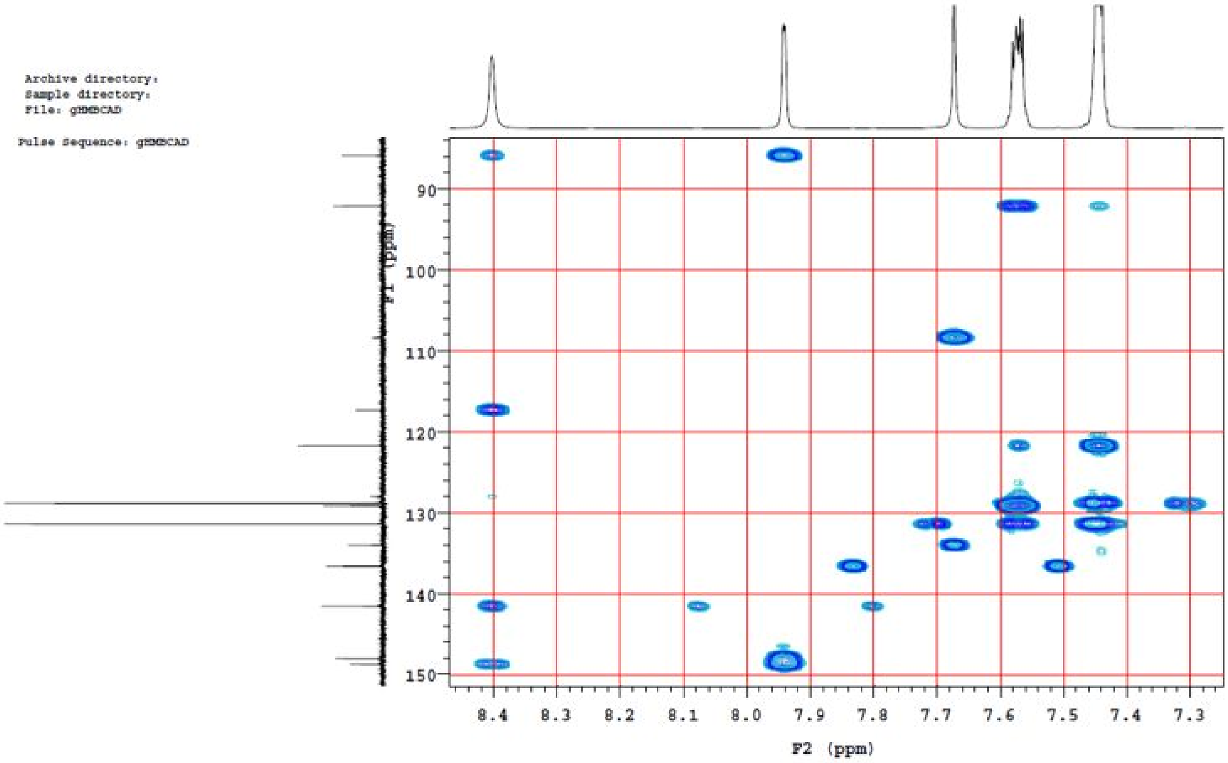
Archive directory:
Sample directory:
File: gHMBCAD
Pulse Sequence: gHMBCAD
F1 (ppm)
F2 (ppm)

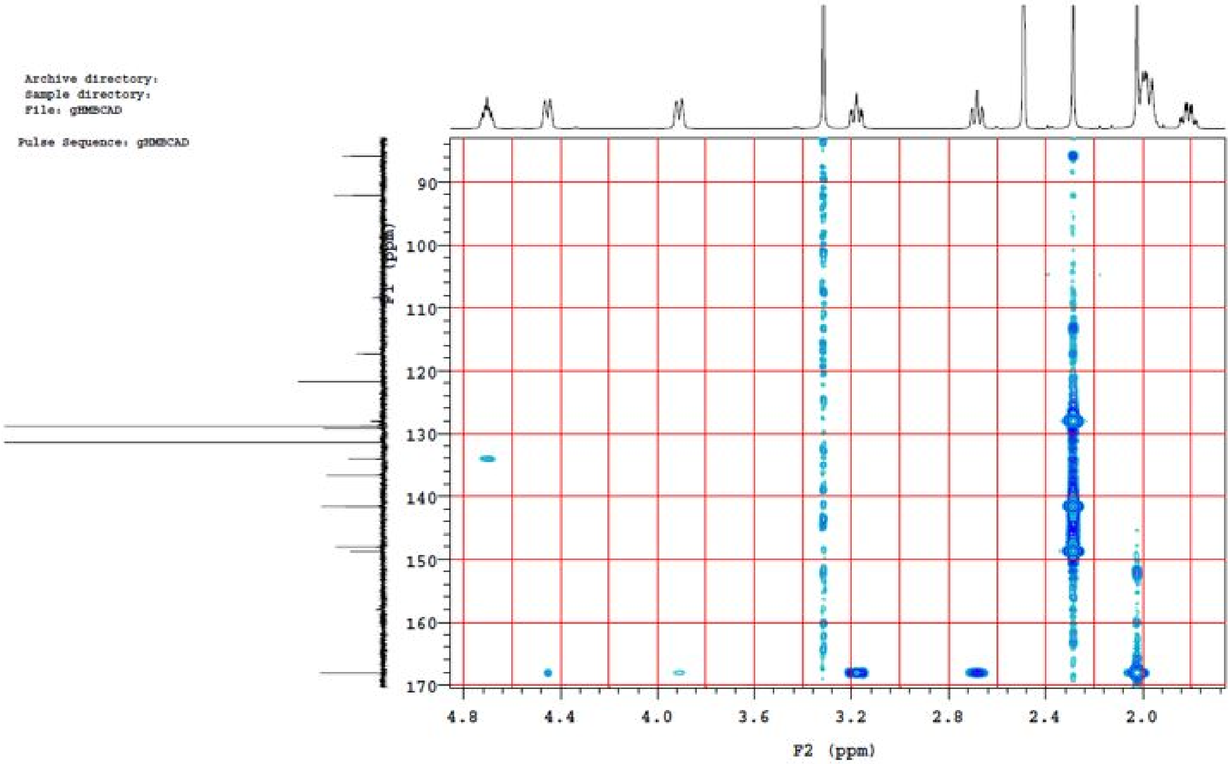
Archive directory:
Sample directory:
File: gHMBCAD
Pulse Sequence: gHMBCAD
F1 (ppm)
F2 (ppm)

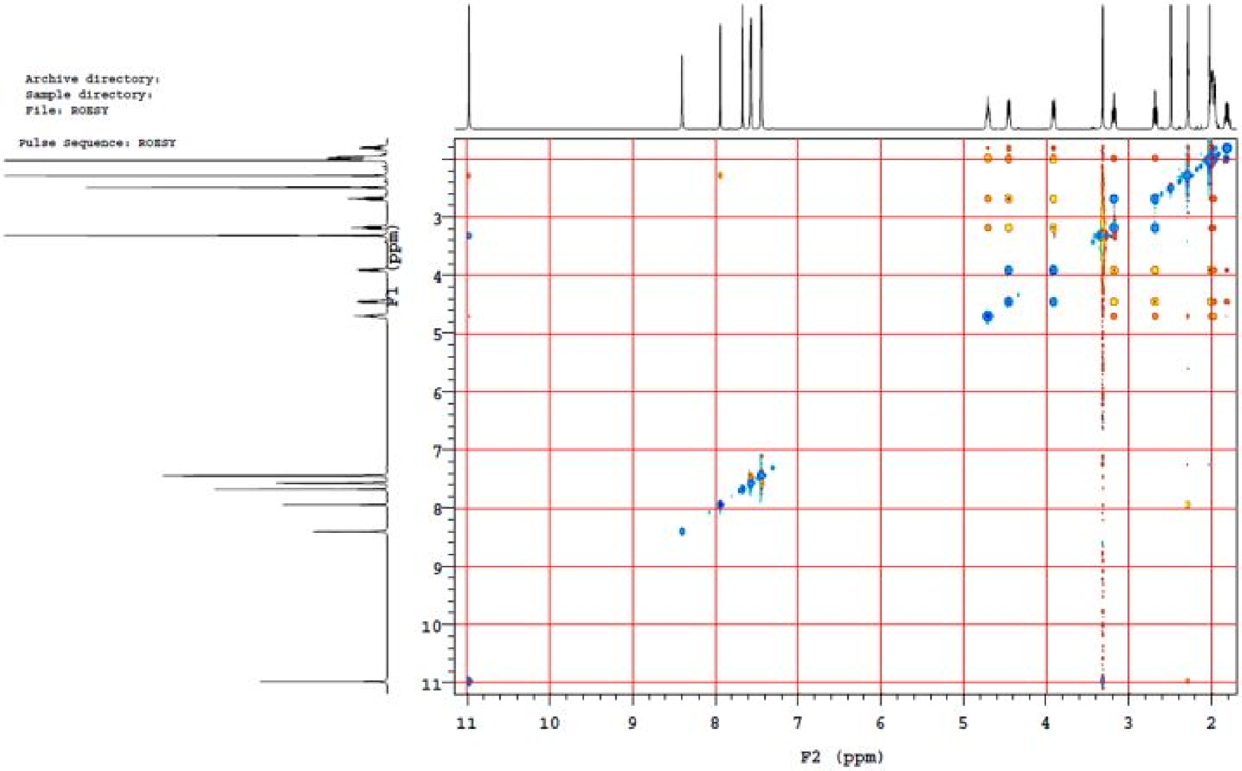


**Characterization of ONO-7927846**

**$^{1}$H NMR Spectra for Compound 11**

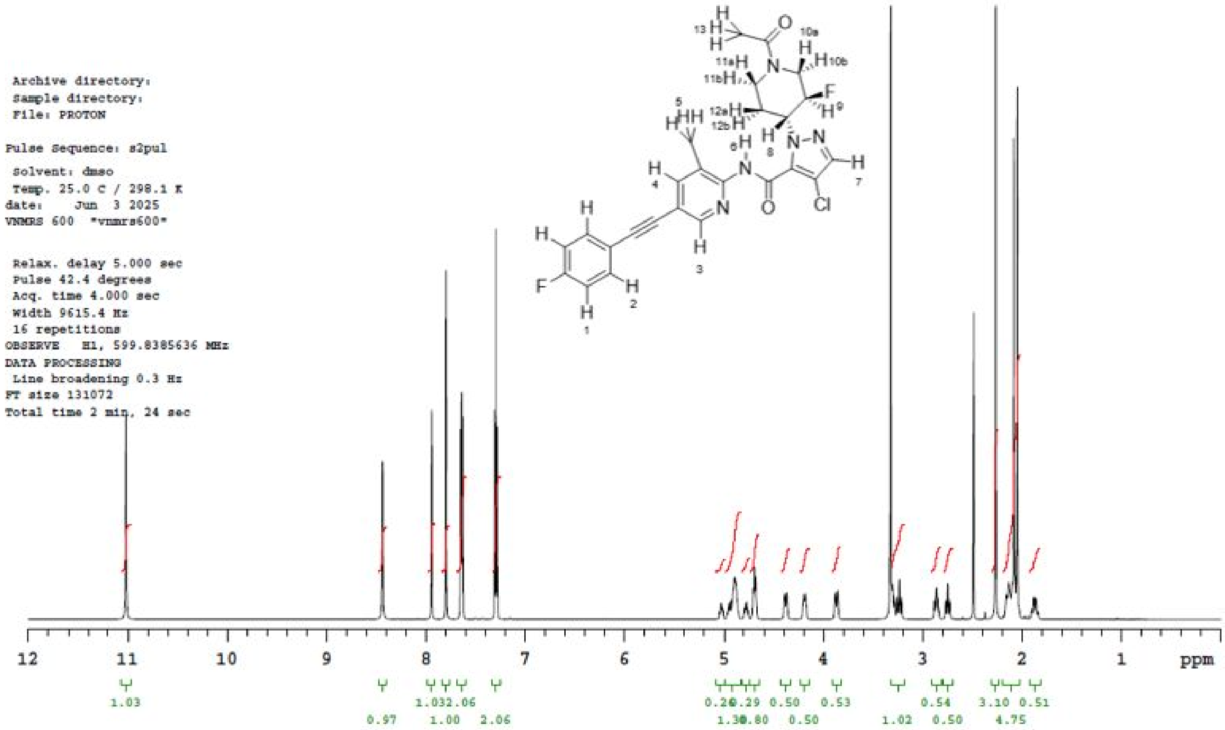
Archive directory:
Sample directory:
File: PROTON
Pulse Sequence: s2pul
Solvent: dmso
Temp. 25.0 C / 298.1 K
date: Jun 3 2025
VNMRS 600 "vnmrs600"
Relax. delay 5.000 sec
Pulse 42.4 degrees
Acq. time 4.000 sec
Width 9615.4 Hz
16 repetitions
OBSERVE H1, 599.8385636 MHz
DATA PROCESSING
Line broadening 0.3 Hz
FT size 131072
Total time 2 min, 24 sec
ppm
Cl
F

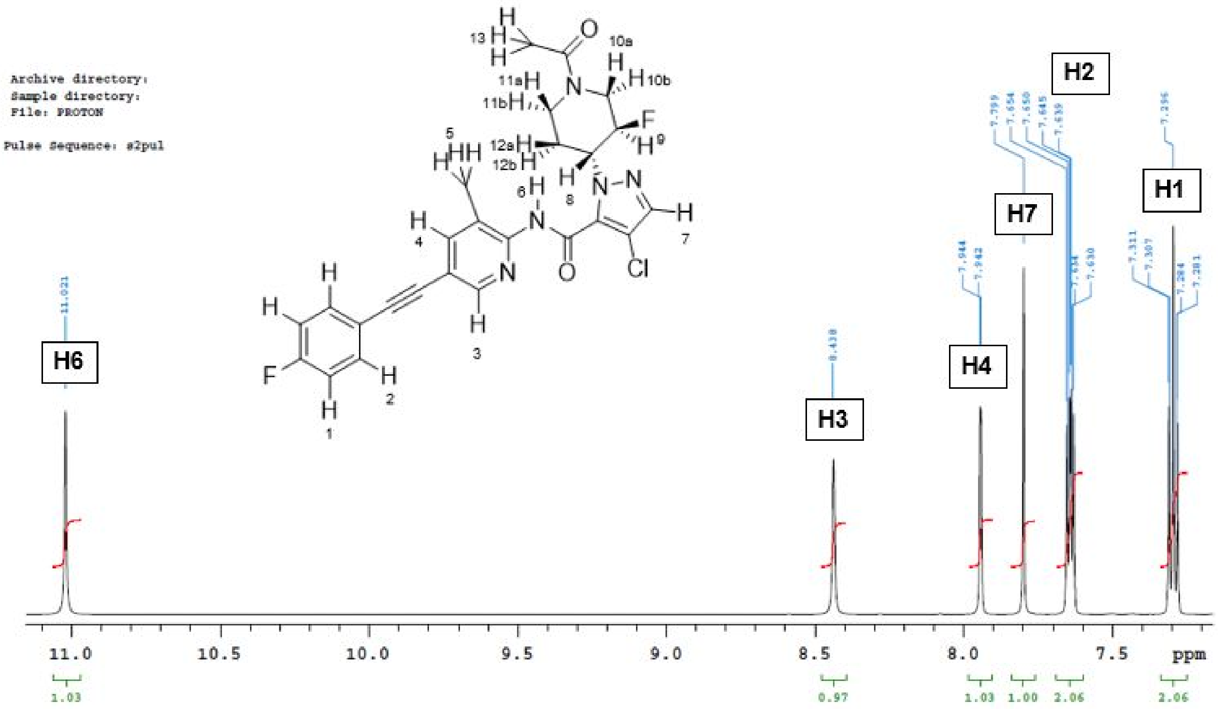
Archive directory:
Sample directory:
File: PROTON
Pulse Sequence: s2pul
H6
H3
H4
H7
H2
H1
ppm
Cl
F

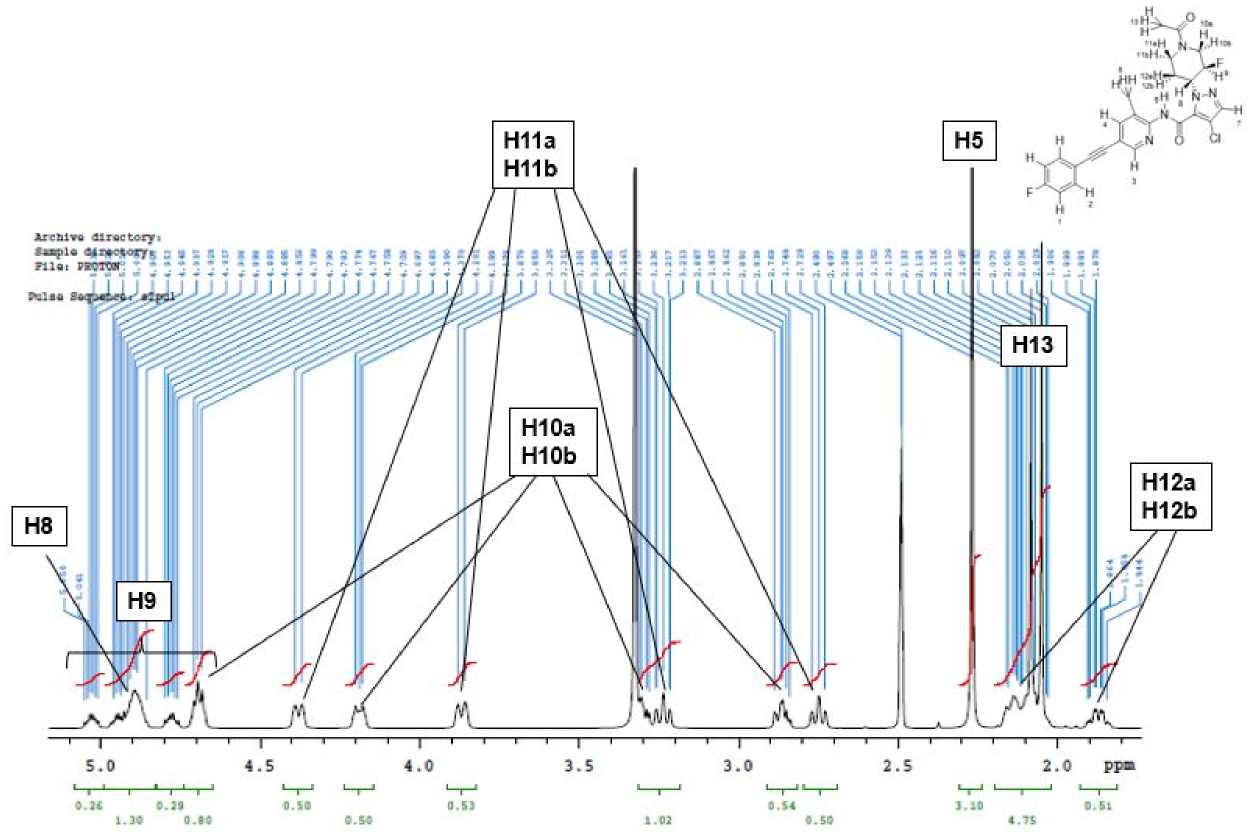


**$^{13}C$ NMR Spectra for Compound 11**

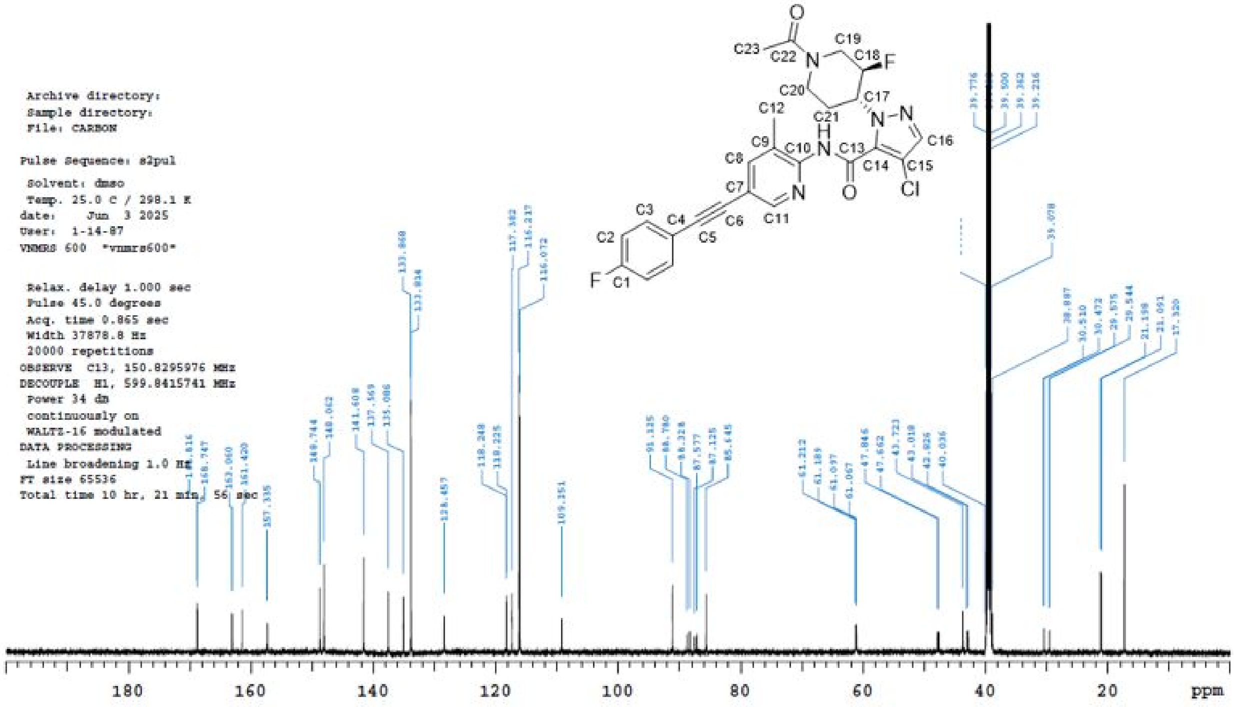
Archive directory:
Sample directory:
File: CARBON
Pulse Sequence: s2pul
Solvent: dmso
Temp. 25.0 C / 298.1 K
date: Jun 3 2025
User: 1-14-87
VNMRS 600 "vnmrs600"
Relax. delay 1.000 sec
Pulse 45.0 degrees
Acq. time 0.865 sec
Width 37878.8 Hz
20000 repetitions
OBSERVE C13, 150.8295976 MHz
DECOUPLE H1, 599.8415741 MHz
Power 34 dB
continuously on
WALTZ-16 modulated
DATA PROCESSING
Line broadening 1.0 Hz
FT size 65536
Total time 10 hr, 21 min, 56 sec
180
160
140
120
100
80
60
40
20
ppm

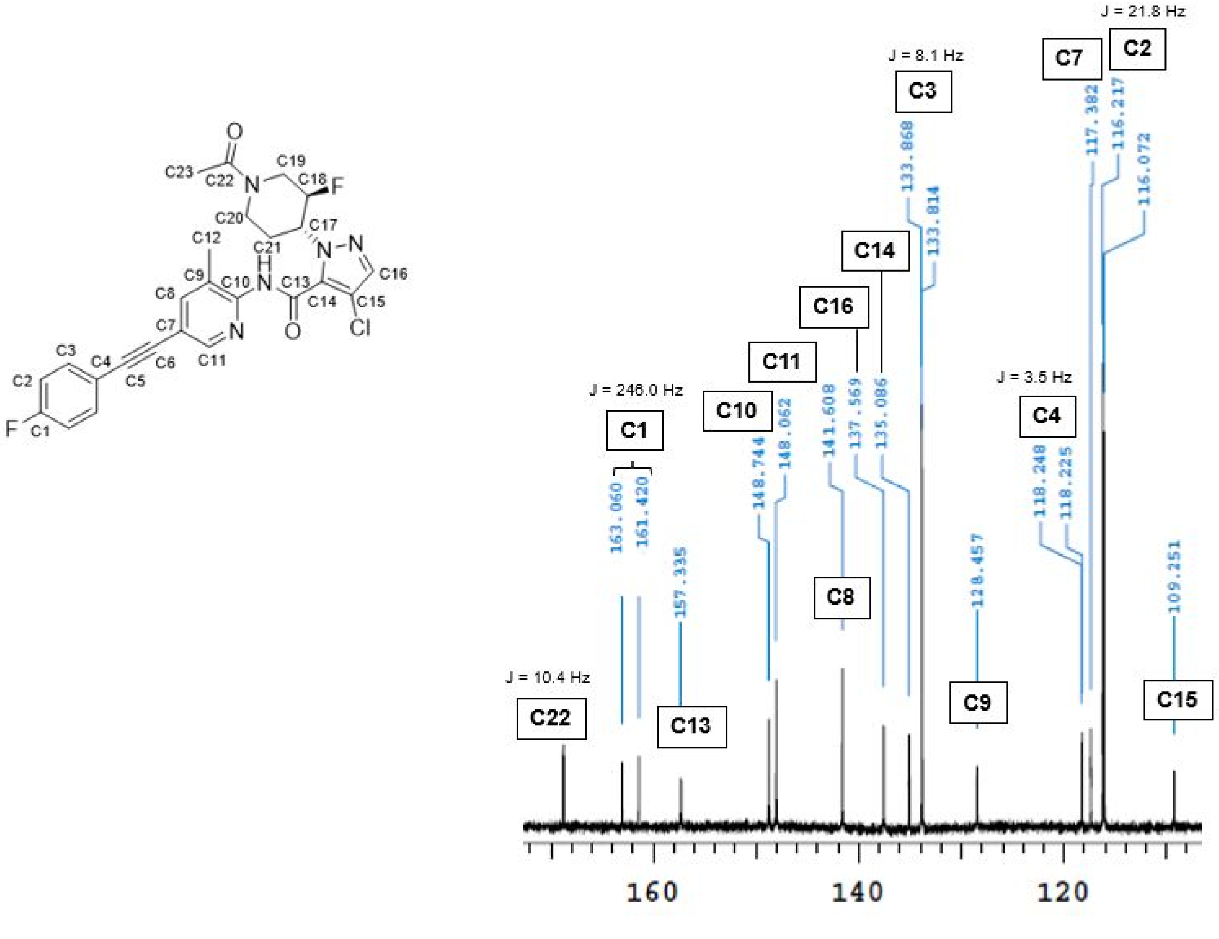
J = 10.4 Hz
C22
J = 248.0 Hz
C1
163.060
161.420
157.335
C13
C10
148.744
148.062
C11
141.608
C8
C16
137.569
C14
135.086
J = 8.1 Hz
C3
133.868
133.814
128.457
C9
J = 3.5 Hz
C4
118.248
118.225
C7
117.382
J = 21.8 Hz
C2
116.217
116.072
109.251
C15
160
140
120

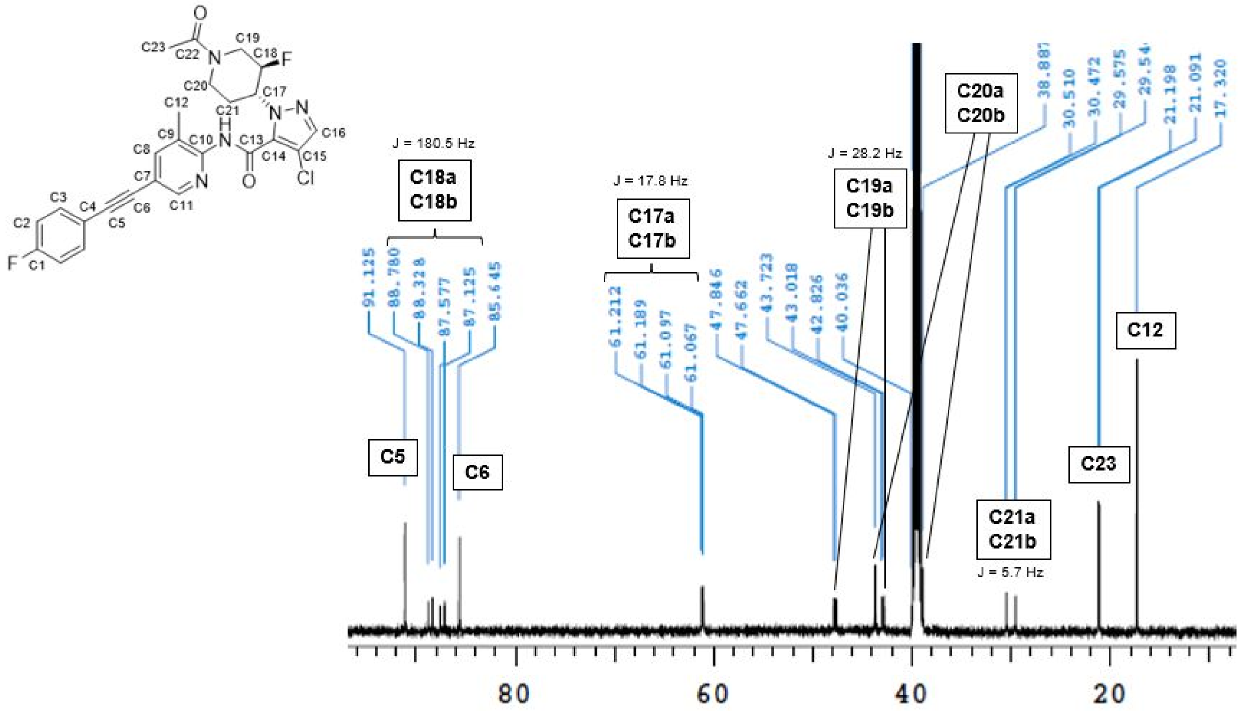
J = 180.5 Hz
C18a
C18b
J = 17.8 Hz
C17a
C17b
J = 28.2 Hz
C19a
C19b
C20a
C20b
91.125
88.780
88.328
87.577
87.125
85.645
61.212
61.189
61.097
61.067
47.846
47.662
43.723
43.018
42.826
40.036
38.887
30.510
30.472
29.575
29.54
21.198
21.091
17.320
C5
C6
C12
C23
C21a
C21b
J = 5.7 Hz
80
60
40
20

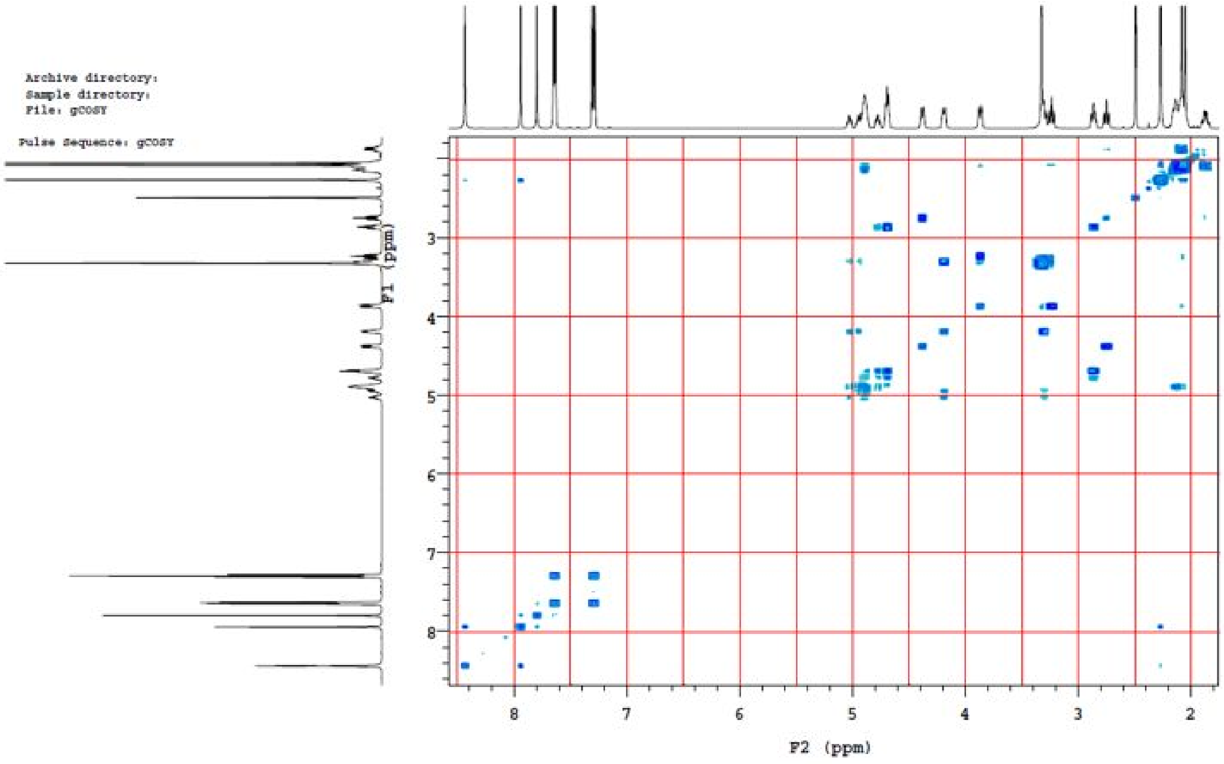
Archive directory:
Sample directory:
File: gCOSY
Pulse Sequence: gCOSY
F1 (ppm)
F2 (ppm)

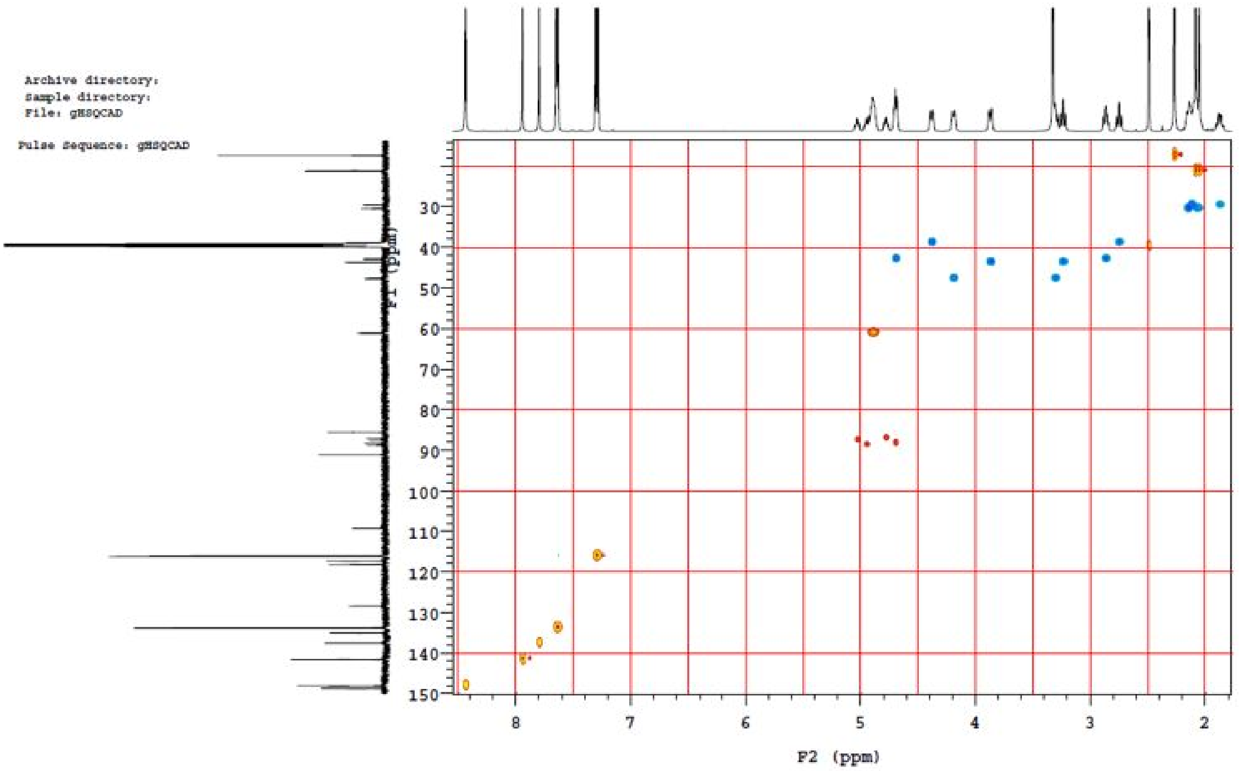
Archive directory:
Sample directory:
File: gHSQCAD
Pulse Sequence: gHSQCAD
F1 (ppm)
F2 (ppm)

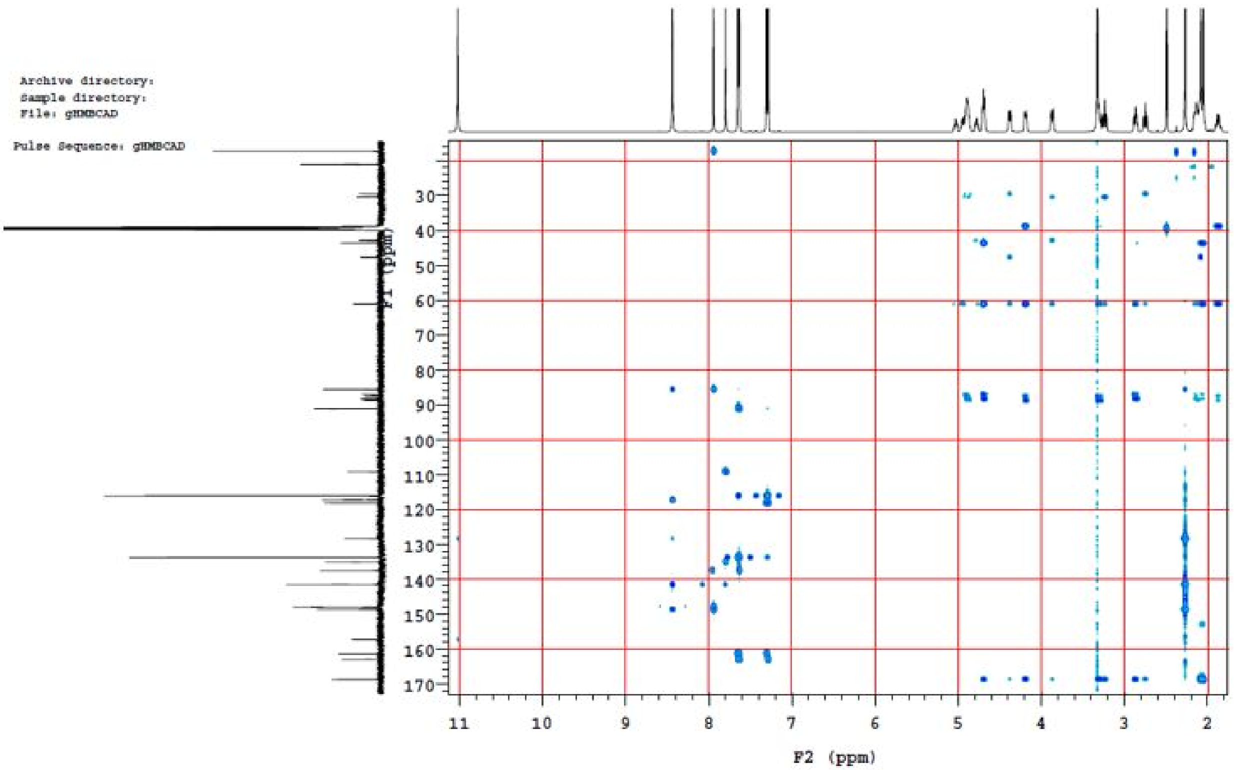
Archive directory:
Sample directory:
File: gHMBCAD
Pulse Sequence: gHMBCAD
F1 (ppm)
F2 (ppm)

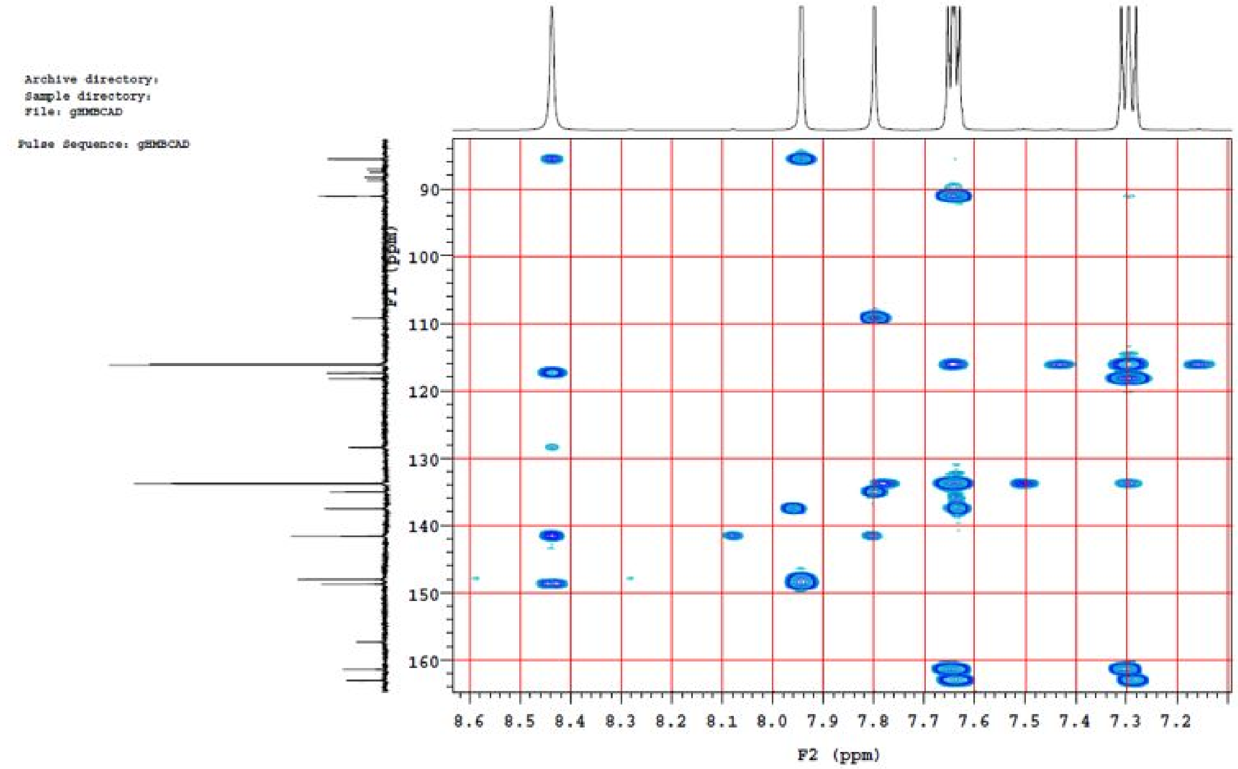
Archive directory:
Sample directory:
File: gHMBCAD
Pulse Sequence: gHMBCAD
F1 (ppm)
F2 (ppm)

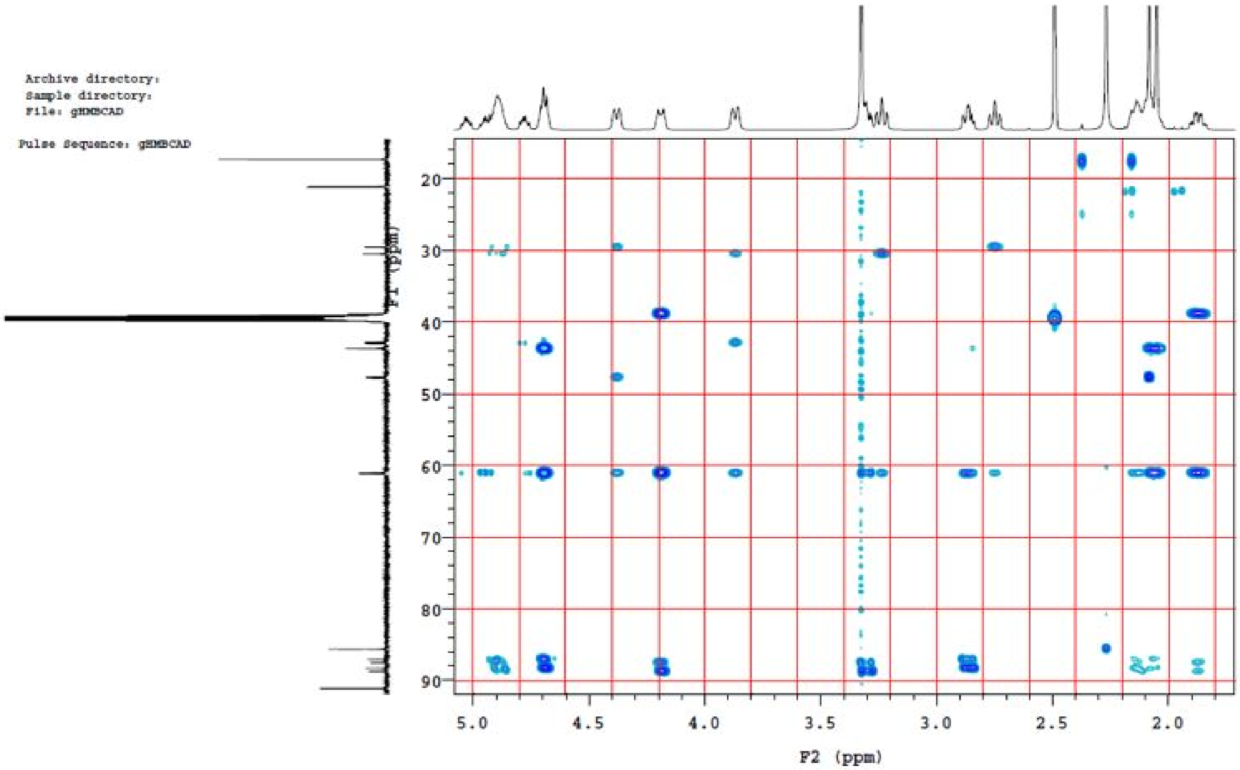
Archive directory:
Sample directory:
File: gHMBCAD
Pulse Sequence: gHMBCAD
F1 (ppm)
F2 (ppm)

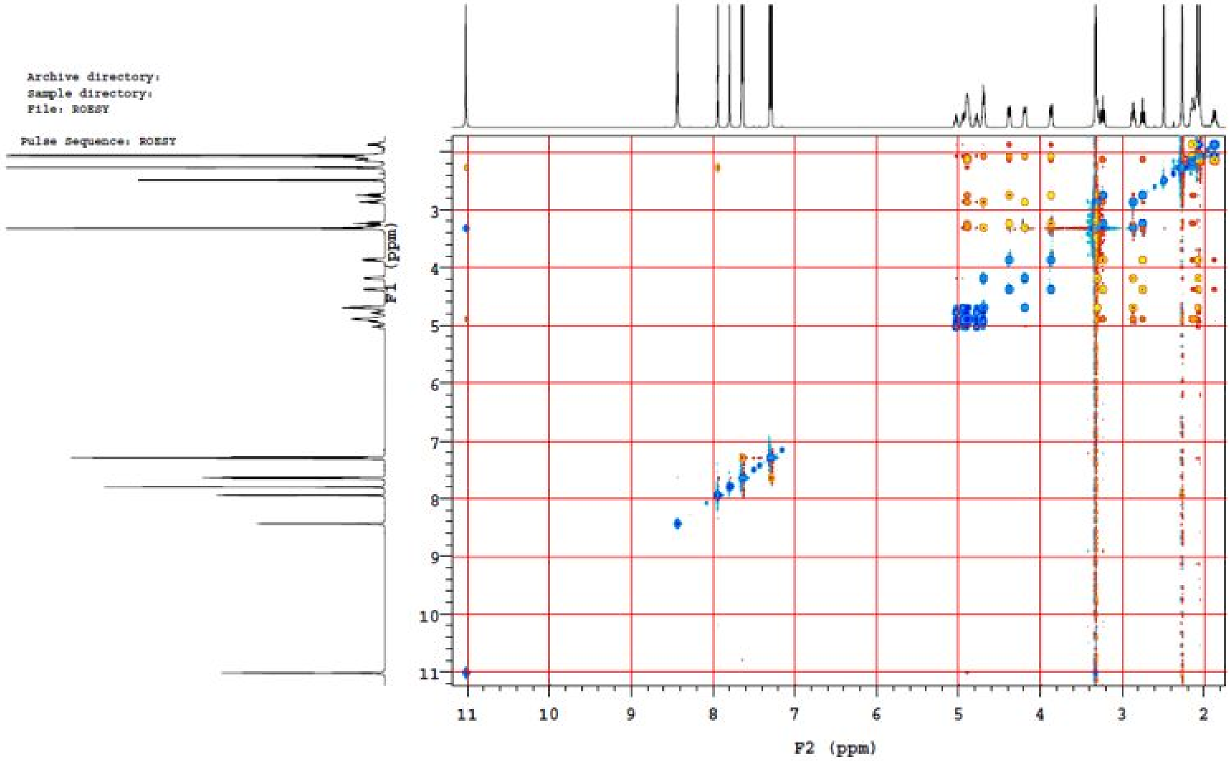


**HPLC trace for ONO-9517601 (Agilent 1200 Systems)**

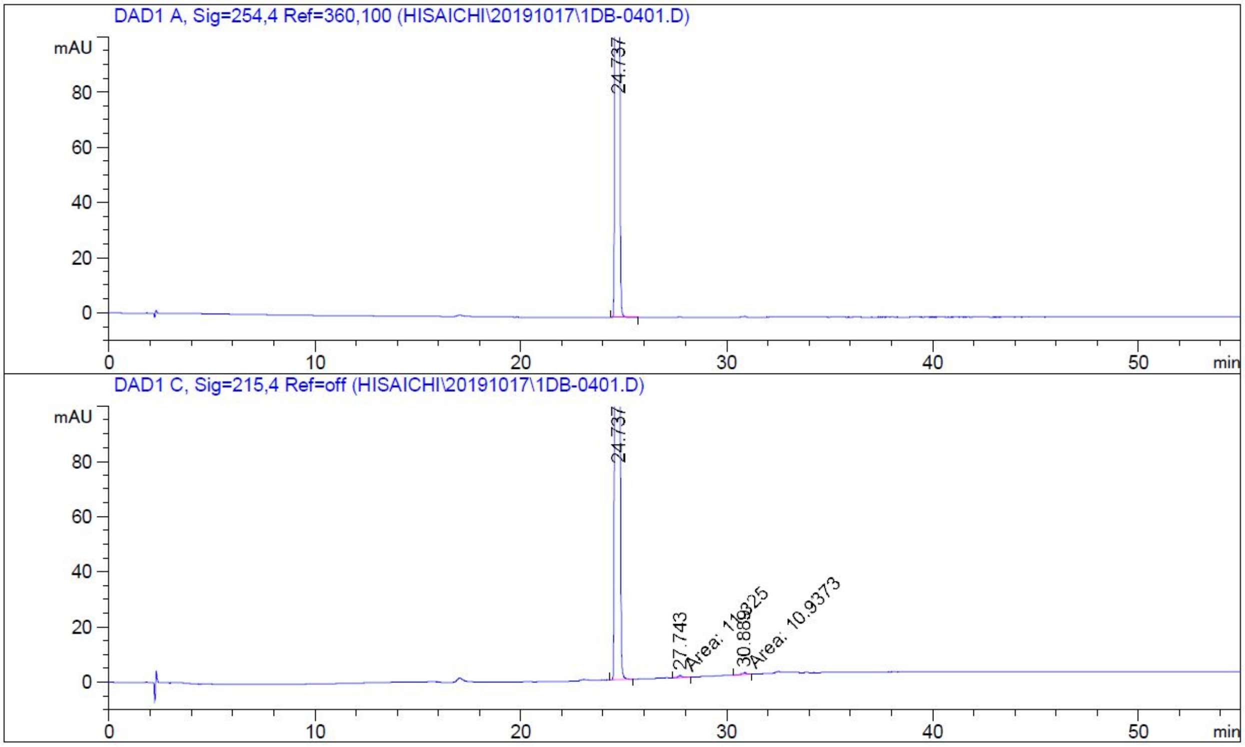

```
Method Info      : Column : Poroshell 120  EC-C18 , 2.7um , 4.6 x 150mm I.D. (Agilent)
                   column temp : 25 deg.
                   UV : 215 nm
                   Solvent A1 : 20mM KH2PO4 (pH 3.0)
                   Solvent B1 : MeCN
                   0.8mL / min
                   inj vol. : 5microL
                   Gradient: B% 10 (0-0.5 min), 80 (30.5 -55 min), 10 (55.1 -60 min)
                   stop time : 55 min
```

Signal 1: DAD1 A, Sig=254,4 Ref=360,100

| Peak # | RetTime [min] | Type | Width [min] | Area [mAU*s] | Height [mAU] | Area % |
|---|---|---|---|---|---|---|
| 1 | 24.737 | BB | 0.1268 | 5771.08105 | 630.15265 | 100.0000 |

Signal 2: DAD1 C, Sig=215,4 Ref=off

| Peak # | RetTime [min] | Type | Width [min] | Area [mAU*s] | Height [mAU] | Area % |
|---|---|---|---|---|---|---|
| 1 | 24.737 | BB | 0.1276 | 1.41105e4 | 1530.57068 | 99.8425 |
| 2 | 27.743 | MM | 0.2395 | 11.32502 | 7.88207e-1 | 0.0801 |
| 3 | 30.889 | MM | 0.2508 | 10.93728 | 7.26693e-1 | 0.0774 |

**HPLC trace for ONO-7927846 (Agilent 1200 Systems)**

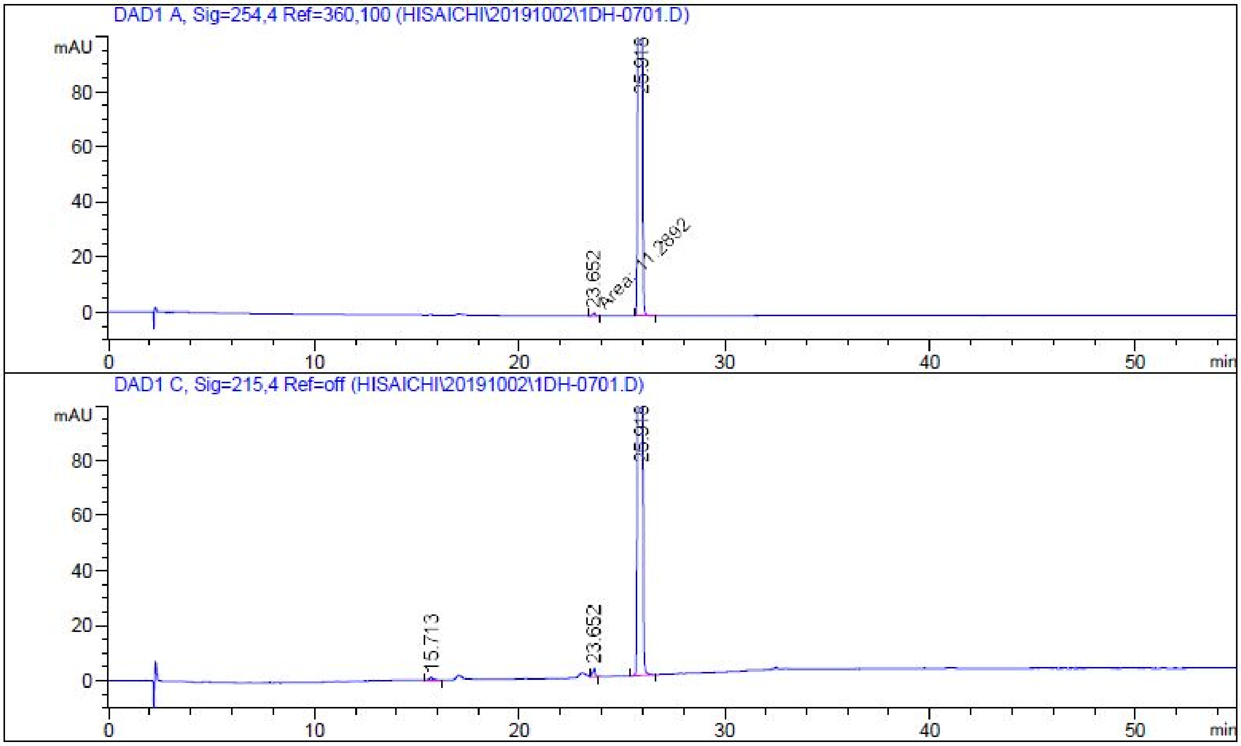

Method Info : Column : Poroshell 120 EC-C18 , 2.7um , 4.6 x 150mm I.D. (Agilent)
column temp : 25 deg.
UV : 215 nm
Solvent A1 : 20mM $KH_2PO_4$ (pH 3.0)
Solvent B1 : MeCN
0.8mL / min
inj vol. : 5microL
Gradient: B% 10 (0-0.5 min), 80 (30.5 -55 min), 10 (55.1 -60 min)
stop time : 55 min

Signal 1: DAD1 A, Sig=254,4 Ref=360,100

| Peak # | RetTime [min] | Type | Width [min] | Area [mAU*s] | Height [mAU] | Area % |
|---|---|---|---|---|---|---|
| 1 | 23.652 | MM | 0.1639 | 11.28922 | 1.14787 | 0.1901 |
| 2 | 25.918 | BB | 0.1223 | 5928.03516 | 675.93579 | 99.8099 |

Signal 2: DAD1 C, Sig=215,4 Ref=off

| Peak # | RetTime [min] | Type | Width [min] | Area [mAU*s] | Height [mAU] | Area % |
|---|---|---|---|---|---|---|
| 1 | 15.713 | BB | 0.1752 | 14.31713 | 1.07643 | 0.1105 |
| 2 | 23.652 | VB | 0.1267 | 28.11177 | 3.07275 | 0.2170 |
| 3 | 25.918 | BB | 0.1229 | 1.29112e4 | 1463.39380 | 99.6725 |
